# Guardians of the canopy: two new turtle ant species (Hymenoptera: Formicidae: *Cephalotes*), from Ecuador’s Chocó and Amazonia, revealed by morphology and DNA barcoding

**DOI:** 10.7717/peerj.21610

**Published:** 2026-09-29

**Authors:** Adrian Troya, Akayky Yumbay, Elian Ilguan, Alex Pazmiño-Palomino, Fernanda Salazar-Buenaño, David A. Donoso

**Affiliations:** 1 Departamento de Biología, Escuela Politécnica Nacional, Quito, Ecuador; 2 Facultad de Ciencias Biologicas, Universidad Central del Ecuador, Quito, Ecuador; 3 Museo de Entomología, Facultad de Recursos Naturales, Escuela Politécnica de Chimborazo, Riobamba, Ecuador; 4 División de Entomología, Instituto Nacional de Biodiversidad, Quito, Ecuador; 5 Instituto Nacional de Pesquisas da Amazônia, Manaus, Brazil; 6 Grupo de Biodiversidad, Museo QCAZ Invertebrados, Pontificia Universidad Católica del Ecuador, Quito, Ecuador

**Keywords:** CO1 gene, Biodiversity conservation, Fogging, Neotropics, Taxonomy

## Abstract

We describe two new species of arboreal ants of the genus *Cephalotes* Latreille, 1802, from Ecuador: *Cephalotes esthelae* Ilguan & Troya, **sp. nov.** and *Cephalotes sacha* Yumbay & Troya, **sp. nov.** The former, a member of the *pinelii* + *grandinosus*-group, was collected from lowland Chocó and Amazonian forests, while the latter, belonging to the *atratus*-group, is an inhabitant of the Amazonian region. Diagnoses and detailed morphological descriptions of workers and soldiers are provided, illustrated by high-resolution images. Species identification was further corroborated with mitochondrial cytochrome c oxidase (CO1) sequences, which confirmed their distinctiveness from morphologically similar congeners. Both species exhibit unique combinations of mesosomal and gastric characters. In addition, we provide the first description of the soldier of the rarely collected *C. dentidorsum* De Andrade, 1999. These discoveries expand the known diversity of the genus in Ecuador to 33 species, highlighting this region’s role as a hotspot for Neotropical myrmecofauna. A fully illustrated key and checklist of all *Cephalotes* species known from Ecuador is also presented. The new taxa occur in well-preserved but increasingly threatened forests, ecosystems subject to high rates of deforestation and mining activity, potentially endangering their populations. The integration of molecular and morphological evidence provides a robust framework for delimiting species such as in this morphologically complex and species-diverse genus. We highlight the need for further exploration in understudied regions such as the Chocó and Andean–Amazonian foothills. Continued morphology- and DNA-based research will be crucial to elucidate phylogenetic relationships, assess biogeographic barriers such as the Andes, and inform conservation strategies for Ecuador’s arboreal ant fauna.

## Introduction

*Cephalotes* Latreille, 1802, usually referred to as turtle ants due to their armor resembling a shell (Price et al., 2014), is currently comprised of 118 extant and 18 fossil, valid species distributed throughout the Neotropical realm, but reaching also the southern Nearctic: Arizona, Texas, and Florida (De Andrade & Baroni-Urbani, 1999; AntWeb, 2025). The countries with most species records of this genus are Brazil (64) and Colombia (45) (AntWeb, 2025), and the biomes with the highest species richness are Amazonia and the Atlantic Forest (AntWeb, 2025).

*Cephalotes* is polymorphic, both morphologically and behaviorally (Corn, 1980), and while species distinction based on workers is relatively straightforward, this is not necessarily the case for the other castes, perhaps because these are not as abundant in collections as are the workers. Recent revisions of the regional *Cephalotes* fauna of Colombia (Sandoval-Gómez & Sánchez-Restrepo, 2019), and Brazil (Oliveira, Powell & Feitosa, 2021) provide excellent tools for the identification of these ants. In general, *Cephalotes* workers are distinguishable from other similar genera, for example *Procryptocerus*, its sister genus, by the presence of strongly expanded frontal lobes, hiding the lateral portions of the clypeus, dorsoventrally slightly flattened bodies, and most species bearing spines or projections on the mesosoma, petiole and postpetiole (De Andrade & Baroni-Urbani, 1999; Fernández, 2003).

*Cephalotes* is adapted to the arboreal life (Wilson, 1976; Sandoval-Gómez & Sánchez-Restrepo, 2019; Butler et al., 2024) with all known species nesting in preexisting cavities in the stems of canopy or understory trees (Powell, 2021). In general, their diet is primarily composed of extrafloral nectar and hemipteran honeydew, but they also routinely consume pollen and nitrogenous vertebrate waste, such as bird droppings and mammalian urine that accumulate on plant surfaces (Powell, 2021). According to Powell (2008), their ecological specialization is based on the colonization of pre-existing wood cavities, which has been particularly important for the evolution of the soldier caste. The soldiers use their armored heads to block the nest entrances against competitors. In contrast, the flattened legs and body with lateral projections present in the workers of many species evolved possibly as a consequence of gliding (Yanoviak, Munk & Dudley, 2011). These integrated adaptations to arboreal life possibly influenced their diversification and evolutionary success, enabling them to occupy a wide range of microhabitats across the Neotropics (Price et al., 2022).

*Cephalotes* was first proposed by Latreille (1802) based on Linnaeus (1758) *Formica atrata* (currently *Cephalotes atratus*). The genus has a long taxonomic history with numerous names being synonymized under it (see Oliveira, Powell & Feitosa, 2021 and De Andrade & Baroni-Urbani, 1999, for a review). Although the monophyly of *Cephalotes* as sister to *Procryptocerus* has been demonstrated before (Moreau et al., 2006; Moreau & Bell, 2013; Ward et al., 2015), its internal classification in 24 species-groups, proposed by De Andrade & Baroni-Urbani (1999), were tested by Price et al. (2014), later confirmed in Price, Etienne & Powell (2016), who showed that some of these groups are not monophyletic. More recently, Price et al. (2022), based on the analysis of ultraconserved elements, proposed an internal conformation of 15 species-groups which need further taxonomic work to stabilize the internal nomenclature of the genus. The current knowledge about the taxonomy and species distribution of *Cephalotes* in northwestern South America is mainly covered by the works of Kempf (1951), De Andrade & Baroni-Urbani (1999), and Sandoval-Gómez & Sánchez-Restrepo (2019). For Ecuador, Salazar et al. (2015) provided the first list with distribution records for 20 species. Subsequently, Pazmiño-Palomino & Troya (2022) recorded six additional species in the country. Since then, some potential new taxa have been identified from several collections in this country.

The present study advances the knowledge of the *Cephalotes* fauna of Ecuador by describing two new species from the coastal and Amazonian regions using an integrative approach based on morphological and molecular evidence. These taxa are assigned to the *pinelii* + *grandinosus* and *atratus* groups (*sensu* Price et al., 2022). Furthermore, we provide the first description of the soldier caste for the rarely collected *C. dentidorsum* De Andrade, 1999. Finally, we build a comprehensive identification key and uptaded checklist for all 33 recorded species in Ecuador.

## Materials and Methods

Portions of the text of our manuscript were previously published as part of a preprint (see https://doi.org/10.64898/2025.12.02.691873).

The new names contained in this work are effectively published under the International Code of Zoological Nomenclature (ICZN). This published work and the nomenclatural acts it contains have been registered in ZooBank, the online registration system for the ICZN. The ZooBank LSIDs (Life Science Identifiers) can be resolved and the associated information viewed through any standard web browser by appending the LSID to the prefix http://zoobank.org/. The LSID for this publication is: urn:lsid:zoobank.org:pub:338E0683-AD9A-441A-80BF-6D38CC5474B1. The online version of this work is archived and available from the following digital repositories: PeerJ, PubMed Central and CLOCKSS.

### Descriptions and material examined

Taxonomic descriptions were generated using the DELTA computer package, version 1.02 (Atlas of Living Australia (ALA), Canberra, ACT, Australia) (Dallwitz, 1980), which converts coded character data into natural language descriptions in Rich Text Format (RTF) for manual editing. The morphometric measurements were organized in a spreadsheet, which also included the collection information, as presented in the ‘Material Examined’ section.

The format of the type material follows: # specimens examined and caste, country, first administrative region, second administrative region or, if known, specific locality, coordinates in decimal degrees, elevation in meters, method of collection (if known), habitat (if known), collection date in format dd-mm-yyyy, collector followed by the word “leg.”, and institution where the material is located, usually represented by a unique specimen identifier composed of capitalized letters and numbers.

We used De Andrade & Baroni-Urbani (1999), Sandoval-Gómez & Sánchez-Restrepo (2019), Oliveira, Powell & Feitosa (2021) as guides for species-level identification. Additionally, we examined previously identified material of the genus in the following collections: Instituto de Investigación de Recursos Biológicos Alexander von Humboldt (IAVH), Villa de Leyva, Colombia; Instituto de Ciencias Naturales, Universidad Nacional de Colombia (ICN), Bogotá, Colombia; Coleção Entomológica Padre Jesus Santiago Moure, Universidade Federal do Paraná (DZUP), Curitiba, Brazil; Colección de Invertebrados del Sur del Ecuador, Universidad Técnica Particular de Loja (CISEC), Loja, Ecuador; Coleção de Formicidae do Centro de Pesquisas do Cacau (CPDC), Ilhéus, Bahia, Brazil; Instituto Nacional de Biodiversidad (MECN), Quito, Ecuador; Laboratorio de Invertebrados, Escuela Politécnica Nacional (MEPN), Quito, Ecuador; Museo de Entomología, Universidad del Valle (MUSENUV), Cali, Colombia; Museo de Invertebrados, Pontificia Universidad Católica del Ecuador (QCAZ), Quito, Ecuador; and Universidad Nacional Mayor de San Marcos (UNMSM), Lima, Peru. Museum abbreviations follow Arnett, Samuelson & Nishida (2019).

We also examined images of type material available on AntWeb (https://www.antweb.org). The types of such material are vouchered in the following institutions: Alex L. Wild Collection (ALWC), Austin, TX, USA; California Academy of Sciences (CASC), San Francisco, CA, USA; John T. Longino Collection (JTLC), Salt Lake City, UT, USA; Philip S. Ward Collection (PSWC), University of California, Davis, CA, USA; Museo del Instituto de Zoología Agrícola (MIZA), Universidad Central de Venezuela, Maracay, Venezuela; Museum of Natural History (MHNG), Geneva, Switzerland; Museo Civico di Storia Naturale di Genova ‘Giacomo Doria’ (MSNG), Genoa, Italy; Natural History Museum (NHMB), Basel, Switzerland; Natural History Museum (NHMUK), London, UK; University of California, Davis (UCDC), Davis, CA, USA; and the National Museum of Natural History, Smithsonian Institution (USNM), Washington D.C., USA. Museum abbreviations follow Arnett, Samuelson & Nishida (2019).

The holotypes and a part of the paratypes are in MEPN (File S1). The other paratypes, whose information is shown in the corresponding type section of each species, will be sent to the following institutions: Museo de Invertebrados, Pontificia Universidad Católica del Ecuador (QCAZ), Quito, Ecuador; Colección de Invertebrados del Sur del Ecuador, Universidad Técnica Particular de Loja (CISEC), Loja, Ecuador; Coleção Entomológica Padre Jesus Santiago Moure, Universidade Federal do Paraná (DZUP), Paraná, Brazil; Instituto Alexander von Humboldt (IAVH), Villa de Leyva, Colombia; Instituto de Ciencias Naturales, Universidad Nacional de Colombia (ICN), Bogotá, Colombia; Museum of Comparative Zoology, Harvard University (MCZC), Massachusetts, USA; United States National Museum of Natural History (USNM), Washington D.C., USA.

The material of the other examined *Cephalotes* species, *i.e*., those we previously identified as valid species-records for Ecuador (File S1), are in the following collections (in order of number of specimen-records): Museo de Invertebrados, Pontificia Universidad Católica del Ecuador (QCAZ); Laboratorio de Invertebrados, Escuela Politécnica Nacional; Instituto Nacional de Biodiversidad (MECN); Colección de Invertebrados del Sur del Ecuador, Universidad Técnica Particular de Loja (CISEC); Museo de la Universidad de Guayaquil (MUGT).

### Imaging

We used a Canon^®^ EOS DSLR 70D camera equipped with a Laowa^®^ 25 mm F2.8, 2.5–5.0× macro lens, coupled to a Cognisys StackShot^®^ macro rail system with a stepper motor controlled by Helicon Remote software v. 3.9.9. The resulting images were stacked with Helicon Focus v. 7.5.6. We used a dome equipped with LED cold lights for illumination. The images of the holotypes and selected paratypes, for example, soldiers, and their respective labels, are available on Antweb.org.

### Measurements and indices

The measurements of the below morphological variables were taken using an ocular micrometer integrated into a KRÜSS MSZ5000-T-IL-TL stereo microscope (A. Krüss Optronic GmbH, Hamburg, Germany) with magnifications ranging from 0.7 to 4.5×. To facilitate visual examination, we used a pin-holding stage that enables rotations along the X, Y, and Z axes. Measurements are given in mm, with an accuracy of two decimals. Minimum and maximum values are provided for each morphometric variable. All measurements are in line with those of Oliveira, Powell & Feitosa (2021). A schematic illustration of such variables is represented in Fig. 1.

**Figure 1:**
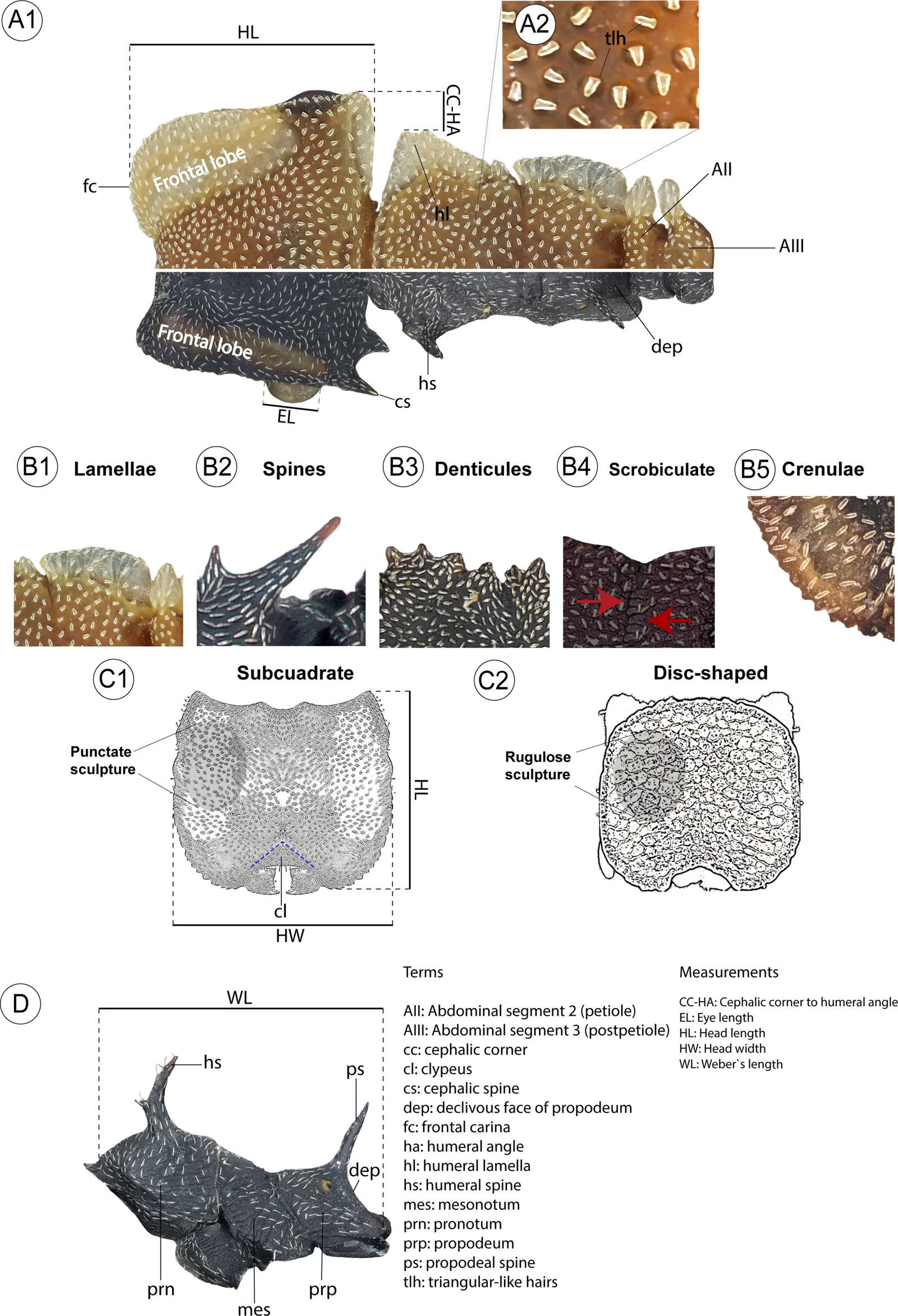
Common morphology structures and measurements cited in text. (A1) Body, except gaster, dorsal view. (A2) Close-up of body hairs. (B1–B5) Sculpture. (C1 and C2) Head shapes. (D) Mesosoma, lateral view. Red arrow-head: scrobiculate. Design by Elian Ilguan.

HW: Head width. In full-face view, maximum width of the cranium. In the workers, this is measured posterior to the eyes, including spines and/or lamellae.
HL: Head length. In full-face view, maximum distance from the posterior head margin, including spines and/or lamellae, to the frontal lobe’s anterior margin.
EL: Eye length. In lateral view, the maximum length of the eye.
PW: Pronotum width. In dorsal view, maximum pronotal width, including pronotal projections and/or lamellae.
WL: Weber’s length. In lateral view, the diagonal length of the mesosoma, from the anterior pronotal margin to the posterior margin of the metapleura.
PTW: Petiolar width. In dorsal view, maximum width of the petiolar node, including petiolar projections and/or lamellae.
PTL: Petiolar length. In dorsal view, the distance between the petiolar anterior most margin to its posterior margin, excluding the posteroventral ridge.
PPW: Postpetiolar width. In dorsal view, maximum width of the postpetiole, including the postpetiolar projections and/or lamellae.
PPL: Postpetiolar length. In dorsal view, distance between the postpetiolar anterior most margin to its posterior margin, excluding the posteroventral ridge.
GW: Gaster width. In dorsal view, maximum width of the first gastral tergite.
GL: Gaster length. In dorsal view, maximum length of the first gastral tergite, including the anterior lamellar margins.
HBW: Hind basitarsus width. Maximum width of the hind basitarsus.
HBL: Hind basitarsus length. Maximum length of the hind basitarsus.
CCHA: Cephalic corner to humeral angle. In dorsal view, horizontal distance between the posterolateral cephalic margin to the lateral humeral margin.
ID: Interspinal distance. In frontal view, distance between the apices of the pronotal spines.
TL: Total length. The summed length of HL + WL + PTL + PPL + GL.

The following ratios were multiplied by 100:

CI: Cephalic index: HW/HL
OI: Ocular index: EL/HW
HBI: Hind basitarsal index. HBW/HBL.
PI: Petiolar index. PTL/PTW

### DNA sequencing and phylogenetic reconstruction

We used the subunit one of the cytochrome c oxidase (CO1) mitochondrial gene complex to infer the possible phylogenetic placement of our new species in the *Cephalotes* CO1 tree, but also their placement within their respective species-groups *sensu* Price et al. (2022): *atratus*-group for *C. sacha* sp. nov., and *pinelii* + *grandinosus*-group for *C. esthelae* sp. nov.

Except for *C. esthelae*, the sequences of all species, including the outgroups, were downloaded from Bold Systems, version 4 (boldsystems.org). Of the 2,300 available sequences we selected only 294 (Files S2 and S3) based on the following criteria: sequences without contamination and stop codons; sequences with more than 300 base pairs; in case of species or morphospecies with numerous sequenced specimens from a single site, we selected only two representatives per site, per country; sequences lacking voucher images were not selected unless these were linked to previously published studies related to the systematics of *Cephalotes*, *e.g*., Price et al. (2014), Oliveira, Powell & Feitosa (2021), Price et al. (2022); and finally, we did not select sequences of species clearly misplaced in the topology when performing prior phylogenetic tests. Of the 294 analyzed sequences, 85 were generated in the present study, and are labeled with “MEPN” in the column “Collection Code” of the File S2.

Genomic DNA of *C. esthelae* sp. nov. was extracted from two legs per specimen using a DNeasy Blood and Tissue Kit (Qiagen Sciences, Germantown, MD, USA), with the following protocol: (1) Legs immersion in 100 µL of lysis solution in a 1.5 mL tube; (2) Legs grinding; (3) Addition of 0.5 µL of proteinase K and vortex for 2 s; (4) Incubation in water bath overnight at 55 °C; (5) Addition of 0.5 µL RNAase and water bath for 30 min/37 °C; (6) Addition of 35 µL of protein precipitation and centrifuge for 3 min/13,000 rpm; (7) Extraction of supernatant and transfer to a sterilized 1.5 mL tube; (8) Addition of 150 µL of isopropanol for DNA precipitation, and centrifuge for 3 min/13,000 rpm; (9) Isopropanol extraction; (10) DNA washing through addition of 150 µL of 70% ethanol, and centrifuge for 3 min/13,000 rpm; (11) Ethanol extraction and drying of ethanol remnants at room temperature for 3–5 min; (12) DNA elution in 20 µL of ultrapure water. The gene region was amplified following a two-step polymerase chain reaction (PCR): first step: 1 min/94 °C; five cycles (30 s/94 °C; 40 s/47 °C; 10 min/72 °C); second step: 30 s/94 °C; 30 cycles (40 s/52 °C; 1 min/72 °C; 10 min/72 °C). Forward primer LCO1490: 5′-GGT CAACAA ATC ATA AAG ATA TTGG-3′ and reverse primer HC02198: 5′-TAA ACT TCA GGG TGACCA AAA AAT CA-3′ from Folmer et al. (1994). The PCRs and DNA isolations were carried out at the Zoologische Staatssammlung München, Germany. The amplicons were sent to the University of Munich’s Biozentrum facility for Sanger sequencing.

The sequences were aligned using MAFFT version 7 (Katoh & Standley, 2013) and the resulting block was visually inspected for unaligned sites which were removed. The length of the final block consisted of 654 base pairs.

We built three trees based on the following sets of sequences: 1. ‘*Cephalotes*_total’ represented by the whole set of 294 sequences (Fig. 2); 2. ‘*Cephalotes*_*atratus*-group’ represented by the available sequenced species in the *atratus* species-group (Fig. 3A); and 3. ‘*Cephalotes*_*pinelii*-*grandinosus*-group’ represented by the available sequenced species in the *pinelii* + *grandinosus* species-group (Fig. 4A). The aligned sequences were submitted to the Cyberinfrastructure for Phylogenetic Research—CIPRES (Miller, Pfeiffer & Schwartz, 2010), and the dataset was analyzed with IQ-TREE (Nguyen et al., 2015; Minh et al., 2020), a maximum likelihood-based tree inference package, using the following parameters: model of molecular evolution TIM2 + F + I + G4, based in the Bayesian information criterium—BIC, as implemented in ModelFinder (Kalyaanamoorthy et al., 2017). The node supports were calculated using 1,000 rounds of ultrafast bootstrap (Hoang et al., 2018) replicates (as recommended in Nguyen et al., 2015), together with the Shimodaira–Hasegawa approximate likelihood ratio test- SH-aLRT, and the Bayesian-like transformation of the aLRT–aBayes (Anisimova et al., 2011) branch support methods. The trees were rooted with species of *Pheidole* (*P. zeteki* Smith, *P. biconstricta* Mayr) and *Procryptocerus* (*P. adlerzi* Mayr, *P. regularis* Emery), which according to Ward et al. (2015) are the closest lineages to *Cephalotes*. The topologies were visualized and edited in FigTree (Rambaut et al., 2018) and Adobe Illustrator^®^ (Adobe Inc., Park Avenue, San Jose, CA, USA).

**Figure 2:**
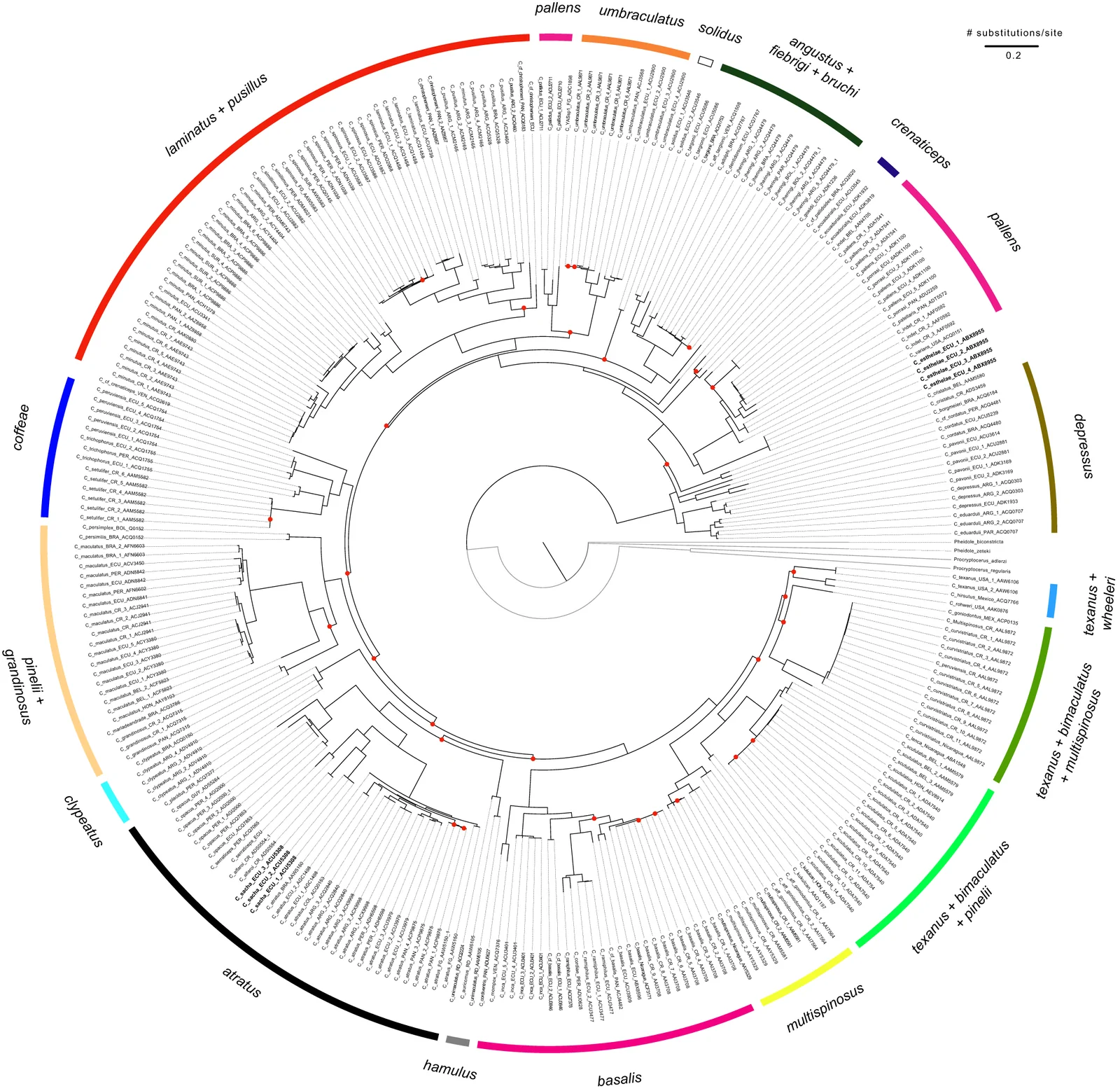
Maximum likelihood CO1 phylogeny of the genus *Cephalotes*. Species-groups are shown on the outer margin. Gray branches are outgroups. Newly described species are bolded. Nodes with <70% support are shown with red dots. The tree tips contain: species name, country abbreviated, BIN (barcode index number).

**Figure 3:**
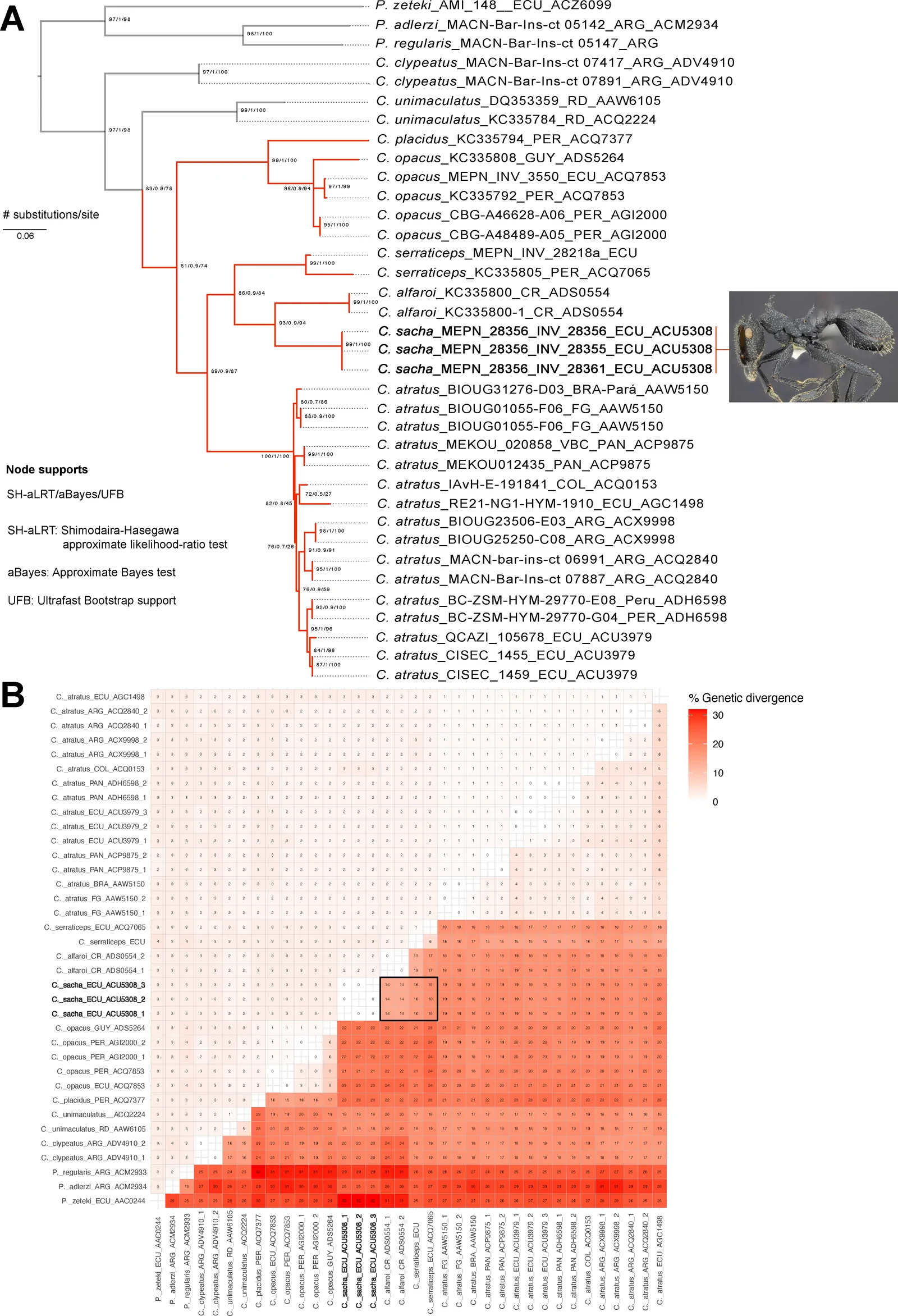
Maximum likelihood CO1 phylogram and genetic divergence of species of the *Cephalotes atratus* species-group. (A) Phylogram. Gray branches: outgroups, red branches: *atratus* group. Tree tips: species name, collection code, country, BIN (barcode index number). Newly described species are bolded. (B) Genetic divergence. Below the diagonal: estimates (in %) of evolutionary divergence between sequences; above the diagonal: standard error estimates. The genetic divergence between *C. sacha* and its closest species (*C. alfaroi* and *C. serraticeps*) is denoted inside a black square.

**Figure 4:**
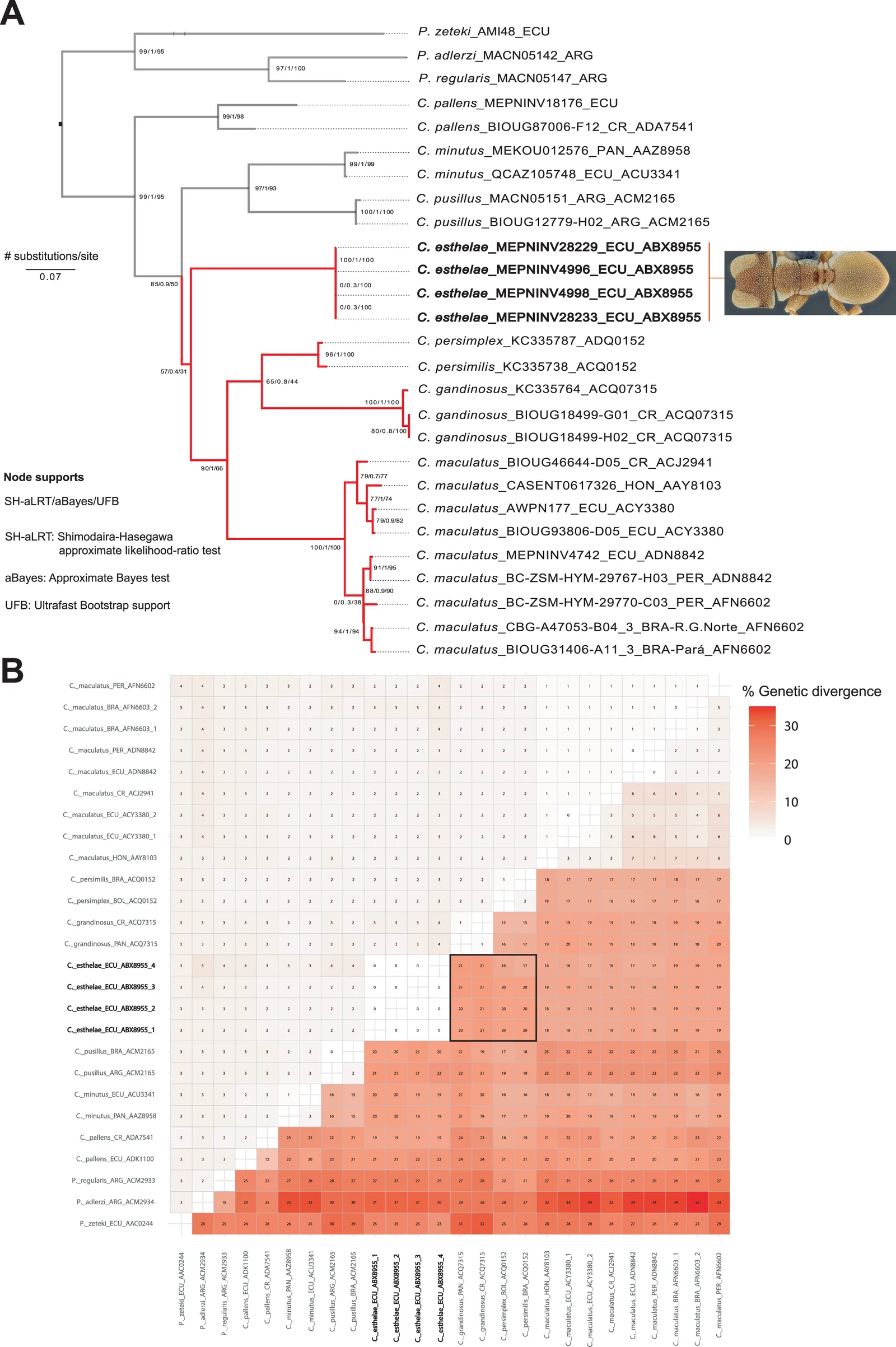
Maximum likelihood CO1 phylogram and genetic divergence of species of the *Cephalotes pinelii + grandinosus* species-group. (A) Phylogram. Gray branches: outgroups, red branches: *pinelii + grandinosus* group. Tree tips: species name, collection code, country, BIN (barcode index number). Newly described species are bolded. (B) Genetic divergence. Below the diagonal: estimates (in %) of evolutionary divergence between sequences; above the diagonal: standard error estimates. The genetic divergence between *C. esthelae* and its closest species (*C. grandinosus*, *C. persimilis*, *C. persimplex*) is denoted inside a black square.

### Distribution of examined records

We used ArcGIS online (https://esri.com) to generate maps of distribution of the examined records based on coordinates written on specimen labels as well as on our own personal field notes. When coordinates were not available, we used Google Earth (Google 2025: https://earth.google.com/web/) to obtain them based on specific localities below the circumscription of province. The coordinates and their associated geographic collection data are shown in File S1.

### Morphology terms

General morphological and caste terminology follows mainly De Andrade & Baroni-Urbani (1999), Oliveira, Powell & Feitosa (2021) and Fernández, Guerrero & Delsinne (2019). Surface sculpture is based on Harris (1979). Pilosity follows Serna & Mackay (2010) for appressed, erect and suberect hairs. Some common morphology terms cited in the text are depicted in Fig. 1.

### Species concept

We follow Mayr’s (1942) concept, which defines species as groups of interbreeding natural populations which are reproductively isolated from others. Since reproductive isolation cannot be directly assessed in the examined material, species boundaries were inferred from morphology. We interpret the examined specimens as being members of new species based on their morphological similarity, expressed in unique sets of external traits differentiating them from all other known closely related species, thus suggesting reproductive isolation.

## Results

### CO1 *Cephalotes* tree and molecular discrimination of the new species

Our CO1 *Cephalotes* tree contains 58 valid species and ten morphospecies (Fig. 2). Overall, within group species composition is in agreement with 11 of the 15 species-groups proposed in Price et al. (2022). These four conflicting groups are the *pallens*, *pinelii* (South American) + *grandinosus*, *texanus* + *bimaculatus* + *pinelii* (Central American), and *wheeleri*. Besides this, the *pallens* and the *pinelii* + *grandinosus* groups came out as paraphyletic in our analysis: *C. pallidus*, a member of the *pallens* group, is sister to *C. umbraculatus* (*umbraculatus* group), whereas the newly described species, *C. esthelae*, came out as sister to most species of the *pallens* group, although from a morphological perspective we suspect it might belong to the *pinelii* + *grandinosus* group (see the diagnosis of this group under the taxonomic account). For this reason, we suggest a provisional placement of *C. esthelae* under such group. Another major disagreement from our analysis with respect to that of Price et al. (2022) consists in the internal arrangement of species-groups. For example, in our phylogeny the *atratus* group is not at the base of the tree and is sister to the *basalis* group. In contrast, in Price et al. (2022), the *atratus* group is recovered as sister to all other species-groups (Fig. 1 of their phylogeny). There are some potential causes for these inconsistencies, among them, the lower phylogenetic resolution of the CO1 above species level. We will expand on this topic in the discussion.

As to the placement of species within each species-group we noted some variations with respect to Price et al. (2022) phylogeny. Price et al. (2022) however, include in their work 24 species and various other morphospecies which we do not include here because there are no available CO1 sequences for them yet. Possibly, the most striking difference in within-group species relationships between our study and that of Price et al. (2022), is in the *pallens* group, where the included species are split in two separate groups: the first consisting of *C. pallens*, *C. porrasi*, *C. patellaris*, and two unidentified species from Belize and Costa Rica, and a second group consisting of *C. pallidus* and an unidentified, possibly new species, *C*. YASsp1, from French Guiana (Fig. 2). The first *pallens* group comes out as sister to the new species, *C. esthelae*, while the second *pallens* group comes out as sister to *C. umbraculatus*, a monotypic species-group.

The new Amazonian species, *C. sacha*, which belongs to the *atratus* species-group, comes out as sister to the Central American *C. alfaroi* in our phylogeny. Both are genetically closer to *C. serraticeps*, the nodal support values for these relationships are relatively high (Fig. 3A). *Cephalotes alfaroi* shows about 14% of genetic divergence from *C. sacha*, as measured by the number of substitutions per site (Fig. 3B). *Cephalotes serraticeps*, on the other hand, which is also morphologically similar to *C. sacha*, has about 16% genetic divergence with it. These three species are closer to *C. atratus* than they are to *C. opacus* and *C. placidus*. *Cephalotes oculatus* is the only member of the *atratus* group not represented in the *atratus* species-group.

*Cephalotes esthelae* is morphologically most similar to *C. persimilis* De Andrade, 1999 and *C. persimplex* De Andrade, 1999 (see further details under the taxonomic treatment of *C. esthelae*), but as said before, it does not come out as sister, not even closer to them in the *Cephalotes* CO1 tree (Fig. 2). *Cephalotes esthelae* shows about 17–20% of genetic divergence from *C. persimilis* and *C. persimplex*, and about 20% from *C. grandinosus* (Fig. 4B), which is also morphologically similar to *C. esthelae*. Despite the morphological resemblance among these four species, *C. esthelae* exhibits a pronounced genetic divergence from them. However, the node subtending this specific lineage shows low support values, indicating that its phylogenetic placement between the *pallens* and *pinelii + grandinosus* groups remains unstable in this topology (Fig. 4A).

**Taxonomic account**

Cephalotes species known to occur in Ecuador, figure citations refer to their distribution. New country records with *

*Cephalotes atratus* (Linnaeus, 1758) (Fig. 5B)

**Figure 5:**
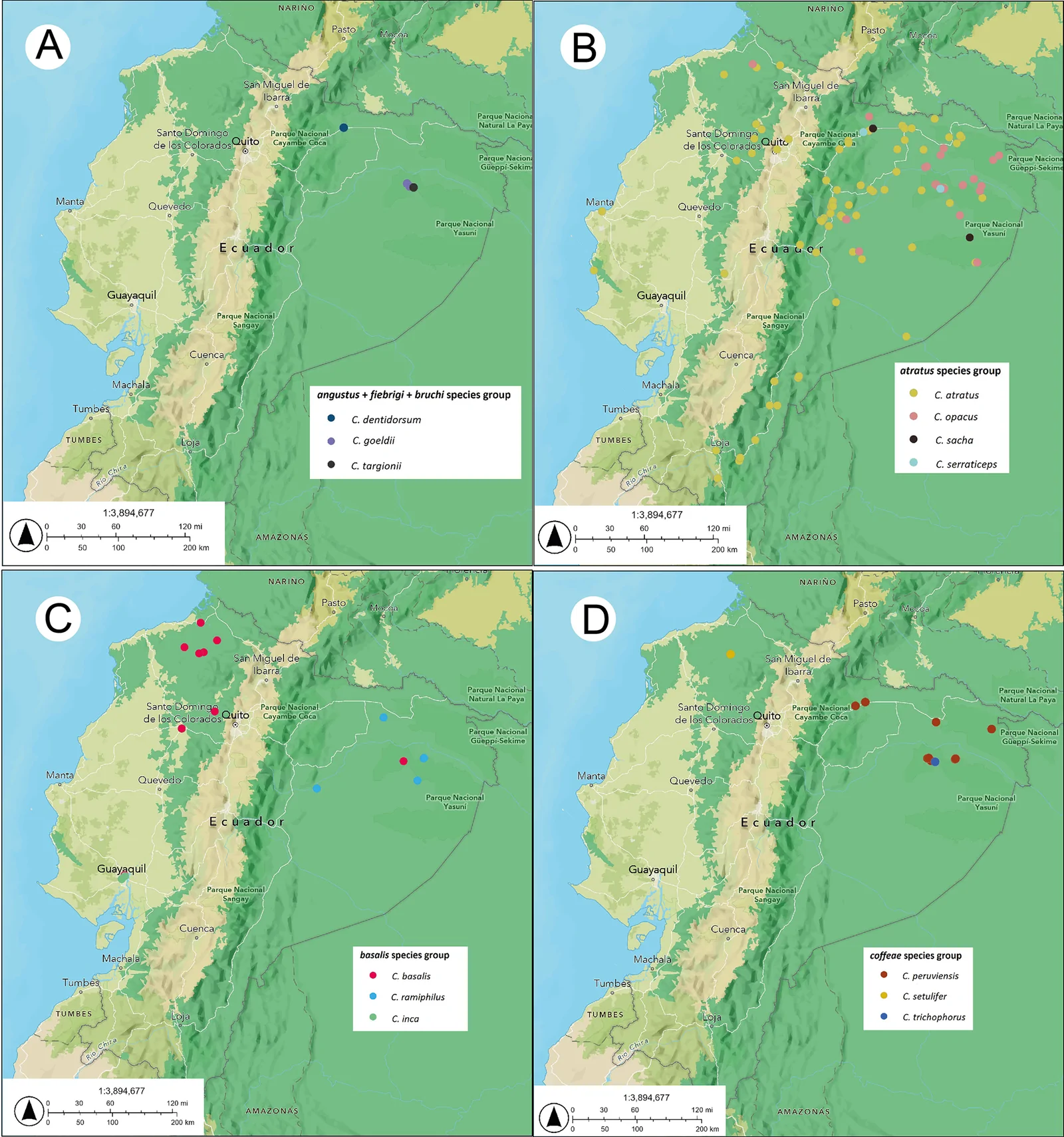
Distribution of examined species records in each species-group. (A) *angustus + fiebrigi + bruchi*. (B) *atratus*. (C) *basalis*. (D) *coffeae*.

*Cephalotes basalis* (Smith, 1876) (Fig. 5C)

*Cephalotes cordatus* (Smith, 1853) (Fig. 6B)

**Figure 6:**
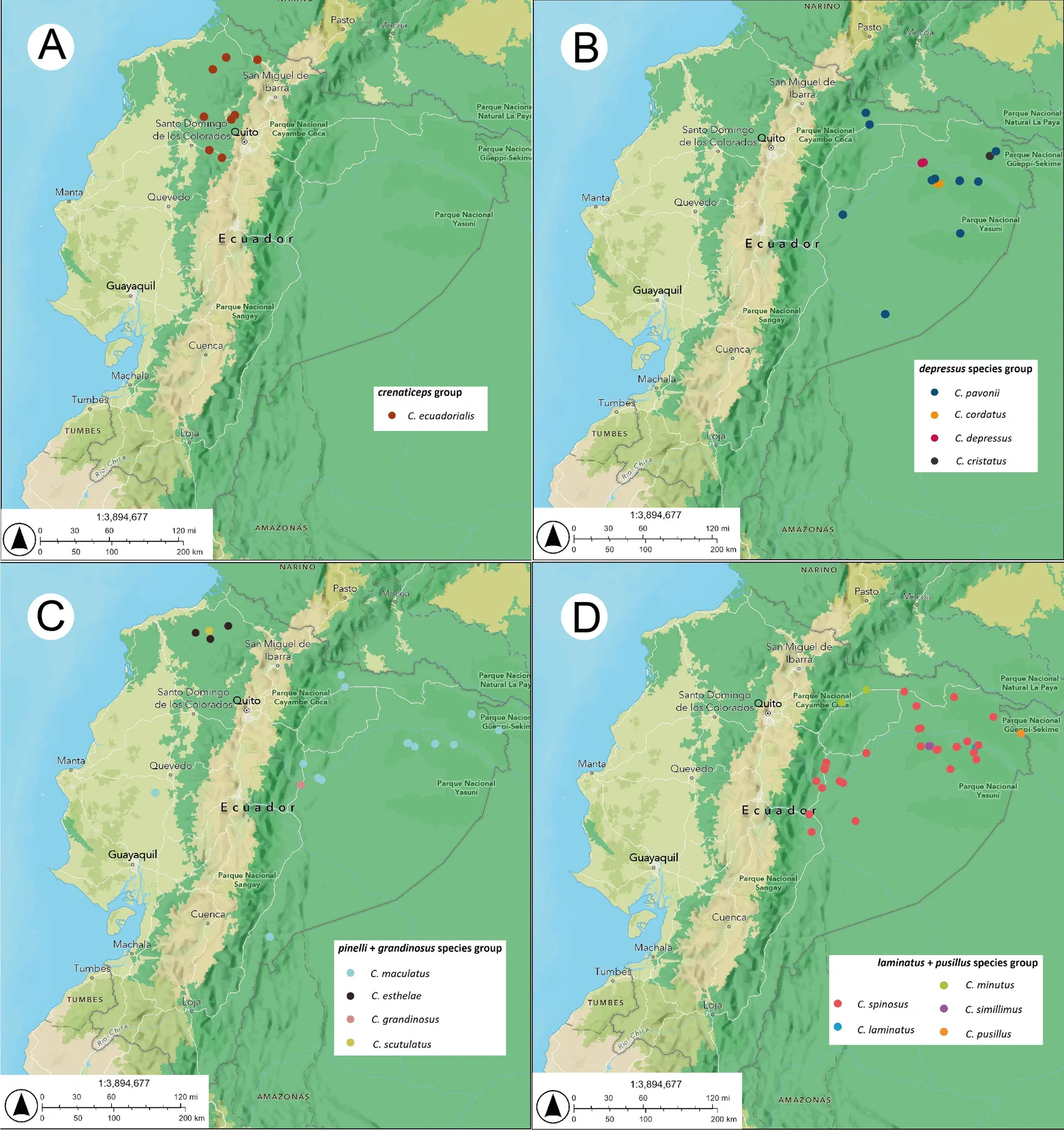
Distribution of examined species records in each species-group. (A) *crenaticeps*. (B) *depressus*. (C) *pinelii + gandinosus*. (D) *laminatus + pusillus*.

*Cephalotes dentidorsum* De Andrade, 1999 (Fig. 5A)

*Cephalotes depressus* (Klug, 1824) (Fig. 6B)

*Cephalotes ecuadorialis* De Andrade, 1999 (Fig. 6A)

*Cephalotes esthelae* **sp. nov.** (Fig. 6C)

*Cephalotes goeldii* (Forel, 1912) (Fig. 5A)

*Cephalotes grandinosus* (Smith, 1860) (Fig. 6C)

*Cephalotes inca* (Santschi, 1911) (Fig. 5C)

*Cephalotes laminatus* (Smith, 1860) (Fig. 6D)

*Cephalotes maculatus* (Smith, 1876) (Fig. 6C)

*Cephalotes manni* (Kempf, 1951) (Fig. 7A)

**Figure 7:**
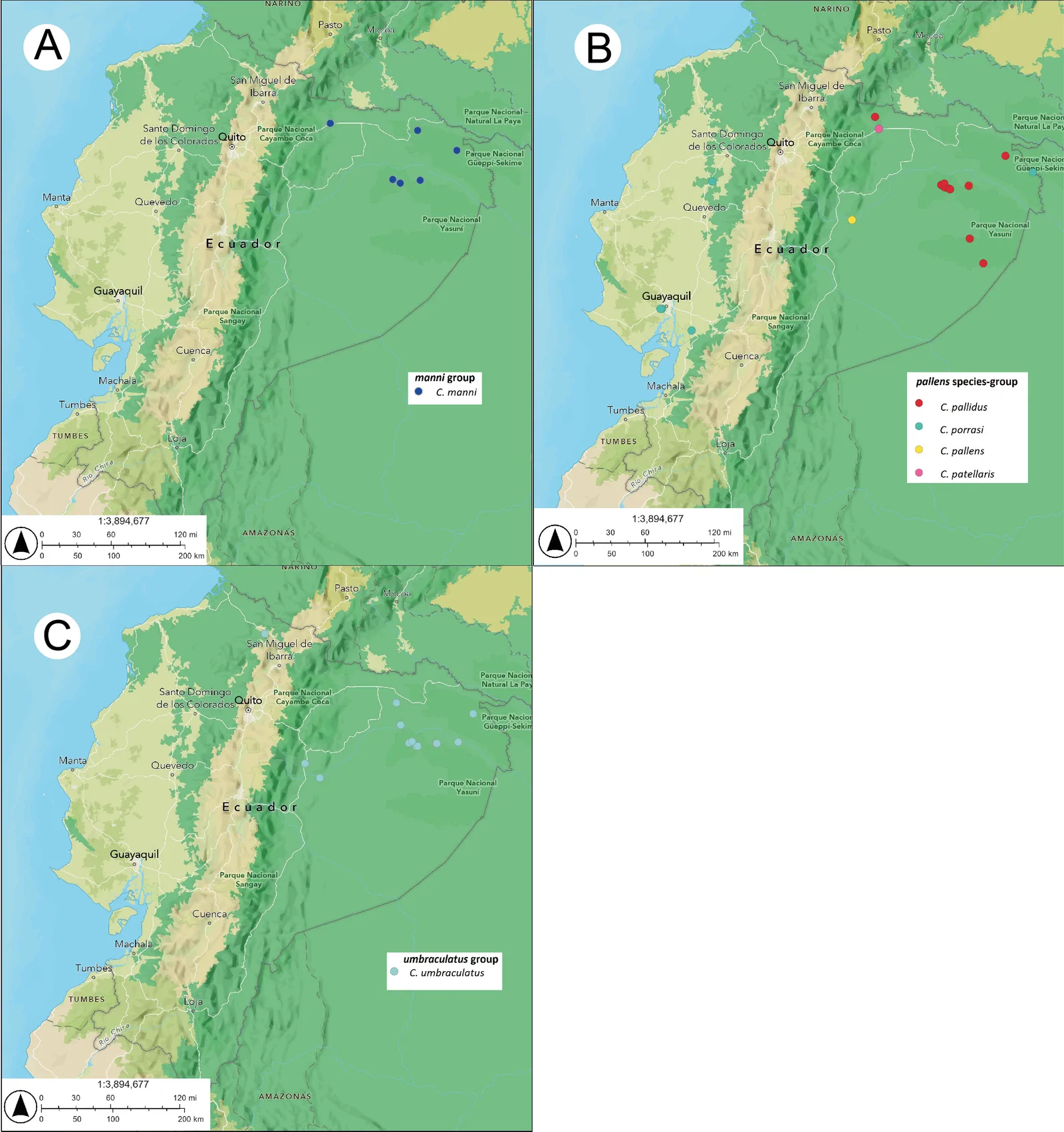
Distribution of examined species records in each species-group. (A) *manni*. (B) *pallens*. (C) *umbraculatus*.

*Cephalotes minutus* (Fabricius, 1804) (Fig. 6D)

*Cephalotes opacus* Santschi, 1920 (Fig. 5B)

*Cephalotes pallens* (Klug, 1824) (Fig. 7B)

*Cephalotes pallidus* De Andrade, 1999 (Fig. 7B)

*Cephalotes patellaris* (Mayr, 1866)* (Fig. 7B)

*Cephalotes pavonii* (Latreille, 1809) (Fig. 6B)

*Cephalotes peruviensis* De Andrade, 1999 (Fig. 5D)

*Cephalotes porrasi* (Wheeler, 1942) (Fig. 7B)

*Cephalotes pusillus* (Klug, 1824) (Fig. 6D)

*Cephalotes ramiphilus* (Forel, 1904) (Fig. 5C)

*Cephalotes sacha* **sp. nov.** (Fig. 5B)

*Cephalotes scutulatus* (Smith, 1867) (Fig. 6C)

*Cephalotes serraticeps* (Smith, 1858) (Fig. 5B)

*Cephalotes setulifer* (Emery, 1894) (Fig. 5D)

*Cephalotes simillimus* (Kempf, 1951) (Fig.6D)

*Cephalotes solidus* (Kempf, 1974) (Fig. 8)

**Figure 8:**
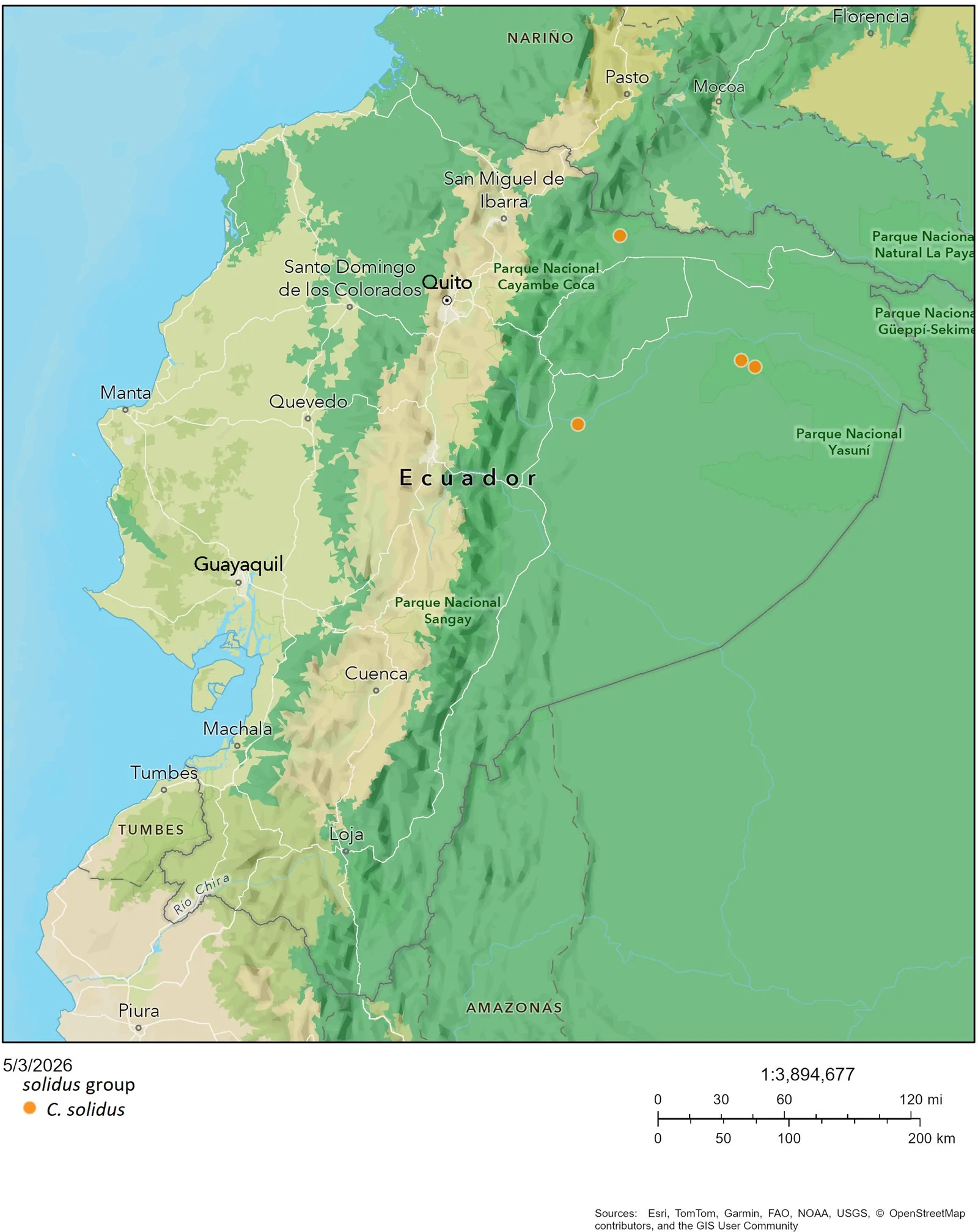
Distribution of examined species records of the *solidus* species-group (monotypic).

*Cephalotes spinosus* (Mayr, 1862) (Fig. 6D)

*Cephalotes targionii* (Emery, 1894)* (Fig. 5A)

*Cephalotes trichophorus* De Andrade, 1999 (Fig. 5D)

*Cephalotes umbraculatus* (Fabricius, 1804) (Fig. 7C)

**Worker-based key to *Cephalotes* species known to occur in Ecuador**
  Next to each species name the species-group (*sensu* Price et al., 2022) is shown in parentheses.
**1**. In full-face view, frontal carina with ocular incision (Fig. 9A) ……………………… **2**
- In full-face view, frontal carina without ocular incision (Fig. 9B)  ……………………… **5**
**2(1).** Head surface rugulose ventrally (Fig. 10A)  ……………………… ***C. patellaris* (Mayr)** **(*pallens* group)**.
- Head surface punctate ventrally (Fig. 10B) ……………………… **3**
**3(2).** In full-face view, frontal lobe with longitudinal striae clearly visible (Fig. 11A); lateral propodeal margin continuous (without teeth), crenulate and membranaceous (Fig. 11C)  ……………………… ***C. pallidus* De Andrade (*pallens* group)**.
- In full-face view, frontal lobe with longitudinal striae barely visible (Fig. 11B); lateral propodeal margin with two or three distinctive membranaceous denticles (Fig. 11D)  ……………………… **4**
**4(3).** In dorsal view, scrobiculate propodeal groove well-marked laterally, absent medially (Fig. 12A)  ……………………… ***C. porrasi* (Wheeler)** **(*pallens* group)**.
- In dorsal view, scrobiculate propodeal groove well-marked along the entire surface (Fig. 12B)  ……………………… ***C. pallens* (Klug)** **(*pallens* group)**.
**5(1).** In full-face view, posterolateral margin of head with squared (Fig. 13A) or sharply bicrested (Figs. 13B and 13C) lamella, either opaque or subhyaline, never with spines ……………………… **16**
- In full-face view, posterolateral margin of head either: continuous or feebly crested (Fig. 13D), sharply crested (Fig. 13E), or with spines (Fig. 13F), never with opaque or subhyaline lamella  ……………………… **6**
**6(5).** In full-face view, posterolateral margin of head with two spines (Fig. 14A)  ……………………… **7**
- In full-face view, posterolateral margin of head without spines, although it can be angulate or carinate (Fig. 14B)  ……………………… **10**
**7(6).** In lateral view, eye placed behind scrobe (Fig. 15A)  ……………………… ***C. opacus* Santschi** **(*atratus* group)**.
- In lateral view, eye placed under scrobe (Fig. 15B)  ……………………… **8**
**8(7).** In dorsal view, pronotum with inverted Y-shaped carina placed anterior to promesonotal groove (Fig. 16A) ……………………… ***C. serraticeps* (Smith)** **(*atratus* group)**.
- In dorsal view, pronotum without inverted Y-shaped carina (Fig. 16B)  ……………………… **9**
**9(8).** Integument opaque; in lateral view, subpostpetiolar process shorter than postpetiolar dorsal spines (Fig. 17A)  ……………………… ***C. sacha* sp. nov. (*atratus* group)**.
- Integument shiny; in lateral view, subpostpetiolar process longer than postpetiolar dorsal denticles (Fig. 17B)  ……………………… ***C. atratus* (*atratus* group)**.
**10(6).** In full-face view, frontal carina crenulate (Fig. 18A)  ……………………… **11**
In full-face view, frontal carina without crenulae, smooth (Fig. 18B)  ……………………… **12**
**11(10).** In dorsal view, humerus with narrow, acute lamella (Fig. 19A); gaster relatively shiny (Fig. 19C); in lateral view, subpostpetiolar process elongated (Fig. 19E)  ……………………… ***C. trichophorus* De Andrade** **(*coffeae* group)**.
- In dorsal view, humerus denticulate (Fig. 19B); gaster opaque (Fig. 19D); in lateral view, subpostpetiolar process short (Fig. 19F)  ……………………… ***C. setulifer* (Emery)** **(*coffeae* group)**.
**12(10).** In dorsal view, mesonotum and propodeum with denticles (Fig. 20A) or spines (Fig. 20B)  ……………………… **13**
- In dorsal view, mesonotum and propodeum without projections (Fig. 20C)  ……………………… ***C. solidus* (Kempf)** **(*solidus* group)**.
**13(12).** In dorsal view, pronotum and propodeum tridenticulate (Fig. 21A)  ……………………… ***C. manni* (*Kempf*) (*manni* group)**.
- In dorsal view, pronotum with lamella, either angulate or rounded anteriorly and posteriorly; propodeum with two spines (Fig. 21B)  ……………………… **14**
**14(13).** In dorsal view, humerus subacute to rounded anteriorly, and rounded posteriorly (Fig. 22A)  ……………………… ***C. ramiphilus* (Forel) (*basalis* group)**.
- In dorsal view, humerus with spine anteriorly, and acute posteriorly (Fig. 22B) ……………………… **15**
**15(14).** In dorsal view, petiolar spine much longer than postpetiolar spine: distance from petiolar spinal tip to postpetiolar spinal tip about as long as mid postpetiolar length (Fig. 23A)  ……………………… ***C. inca* (Santschi) (*basalis* group)**.
- In dorsal view, petiolar spine subequal to- or at most slightly longer than postpetiolar spine: distance from petiolar spinal tip to postpetiolar spinal tip clearly shorter than mid postpetiolar length (Fig. 23B)  ……………………… ***C. basalis* (Smith)** **(*basalis* group)**.
**16(5).** In dorsal view, propodeum with single, broad lamella (Fig. 24A)  ……………………… **17**
In dorsal view, propodeum with one or more denticles (Fig. 24B) or spines (Fig. 24C)  ……………………… **20**
**17(16).** In dorsal view, pronotal lamella truncate or with incision anterolaterally (Fig. 25A)  ……………………… **18**
- In dorsal view, pronotal lamella continuous, without incision anterolaterally (Fig. 25B)  ……………………… **19**
**18(17).** In dorsal view, mesonotum with triangular, subhyaline lamella; petiole and postpetiole with subhyaline, rhomboid-shaped lamella (Fig. 26A)  ……………………… ***C. esthelae* sp. nov.** **(*pinelii* + *grandinosus* group, provisional placement)**.
- In dorsal view, mesonotum with denticle; petiole and postpetiole with spines (Fig. 26B)  ……………………… ***C. scutulatus* (Smith)** **(*texanus* + *bimaculatus* + *pinelii* group)**.
**19(17).** In dorsal view, mesonotum with two lamellae, the anterior is bigger than the posterior (Fig. 27A); hind femur with crenulate lamella at anterior and posterior margins (Fig. 27C)  ……………………… ***C. grandinosus* (Smith)** **(*pinelii* + *grandinosus* group)**.
- In dorsal view, mesonotum with single denticle or lamella (Fig. 26B); hind femur without crenulate lamellae (Fig. 27D)  ……………………… ***C. maculatus* (Smith)** **(*pinelii* + *grandinosus* group)**.
**20(16).** In dorsal view, gaster with two dark linear spots, connected medially by dark spot (Fig. 28A)  ……………………… ***C. umbraculatus* (Fabricius) (*umbraculatus* group)**.
- In dorsal view, gaster concolored (Fig. 28B) or heterocolored (Fig. 28C), without two dark linear spots  ……………………… **21**
**21(20).** In dorsal view, propodeum with single denticle (Fig. 29A)  ……………………… ***C. ecuadorialis* De Andrade** **(*crenaticeps* group)**.
- In dorsal view, propodeum with two or more denticles (Fig. 29B) or spines (Fig. 29C)  ……………………… **22**
**22(21).** In dorsal view, propodeum with two spines (Fig. 30A)  ……………………… **23**
- In dorsal view, propodeum with denticles: the median denticle usually longer than the anterior and posterior denticles (Fig. 30B)  ……………………… **30**
**23(22).** In dorsal view, posterior propodeal spine longer than anterior spine (Fig. 31A)  ……………………… **24**
- In dorsal view, posterior propodeal spine shorter than anterior spine (Fig. 31B)  ……………………… **28**
**24(23).** In dorsal view, mesonotum without denticle (Fig. 32A) ……………………… ***C. spinosus* (Mayr)** **(*laminatus* + *pusillus* group)**.
- In dorsal view, mesonotum with denticle or spine (Fig. 32B)  ……………………… **25**
**25(24).** In dorsal view, declivous face of propodeum with marked longitudinal striae (Fig. 33A)  ……………………… ***C. minutus* (Fabricius)** (***laminatus* + *pusillus* group)**.
- In dorsal view, declivous face of propodeum without striae, but it can have minute puncta (Fig. 33B)  ……………………… **26**
**26(25).** Petiole with tiny denticle laterally (Fig. 34A)  ……………………… ***C. pusillus*** (**Klug)** (***laminatus* + *pusillus* group)**.
- Petiole with distinctive spine laterally (Fig. 34B)  ……………………… **27**
**27(26).** In dorsal view, posterior margin of head and first tergite of gaster with subhyaline lamellae (Fig. 35A) ……………………… ***C. laminatus* (Smith)** **(*laminatus* + *pusillus* group)**.
- In dorsal view, posterior margin of head and first tergite of gaster without subhyaline lamellae, this is, lamellae are concolorous with rest of body or at most, with light brown shades on lamellar margins (Fig. 35B)  ……………………… ***C. simillimus* (Kempf)** **(*laminatus* + *pusillus* group)**.
**28(23).** In posterior view, vertex with two denticles (Fig. 36A); pronotal lamellar margin mostly plain (Fig. 36C)  ……………………… ***C. cordatus* (Smith)** **(*depressus* group)**.
- In posterior view, vertex without denticles (Fig. 36B); pronotal lamellar margin bidentate (Fig. 36D) or crenulate (Fig. 36E)  ……………………… **29**
**29(28).** Pronotum with bidenticulate lamella; a deep notch is present between lamella and posterior pronotal teeth (Fig. 37A)  ……………………… ***C. depressus* (Klug) (*depressus* group)**.
- Pronotum with broad, crenulate lamella; a shallow notch is present between lamella and posterior pronotal teeth (Fig. 37B)  ……………………… ***C. pavonii* (Latreille)** **(*depressus* group)**.
**30(22).** In dorsal view, propodeum with four to five well-developed denticles, with the second and fourth longer than the rest (Fig. 38A)  ……………………… **31**
- In dorsal view, propodeum with three or less well-developed denticles, the median is always longer than the rest (Fig. 38B) Note: Denticles not always well-visible  ……………………… ***C. peruviensis* De Andrade (*coffeae* group)**.
**31 (30).** In dorsal view, pronotum with spine anteriorly, followed by broad, crenulate lobe upturned (Fig. 38A), more evident in lateral view (Fig. 39C)  ……………………… ***C. dentidorsum* De Andrade (*angustus* + *fiebrigi* + *bruchi* group)**.
- In dorsal view, pronotum with three membranaceous denticles (Fig. 39B), without upturned lobe (Fig. 35D)  ……………………… **32**
**32(31).** In dorsal view, postpetiole with wing-shaped, lateral lamella, recurved and broad distally (Fig. 40A)  ……………………… ***C. goeldii* (Forel)** **(*angustus* + *fiebrigi* + *bruchi* group)**.
- In dorsal view, postpetiole with thinner, lateral spine, not wing-shaped (Fig. 40B)  ……………………… ***C. targionii* (Emery)** **(*angustus* + *fiebrigi* + *bruchi* group)**.

**Figure 9:**
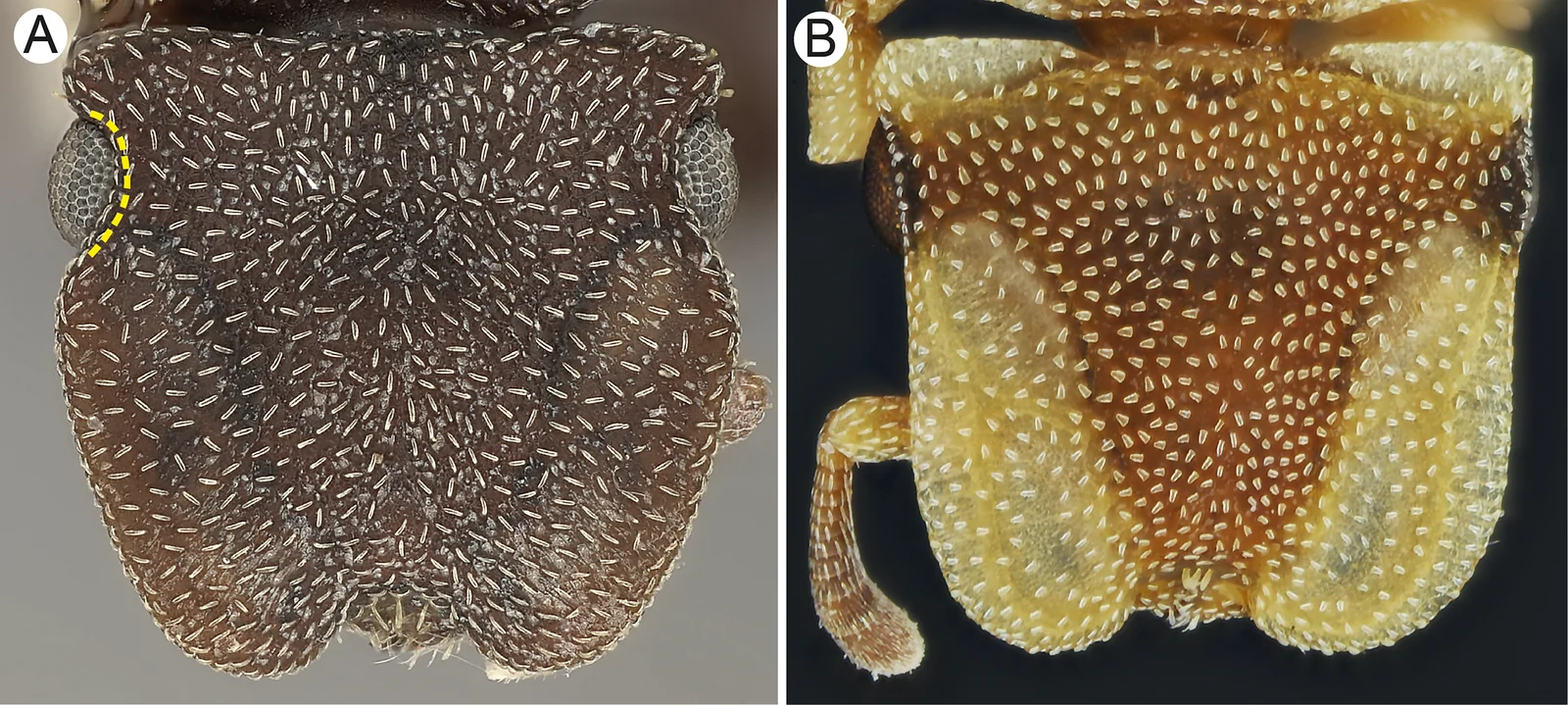
Frontal carina (full-face view). (A) Notched (yellow dotted line). *Cephalotes pallens*. Image by Shannon Hartman. (B) Plain. *Cephalotes esthelae*. Image by Elian Ilguan.

**Figure 10:**
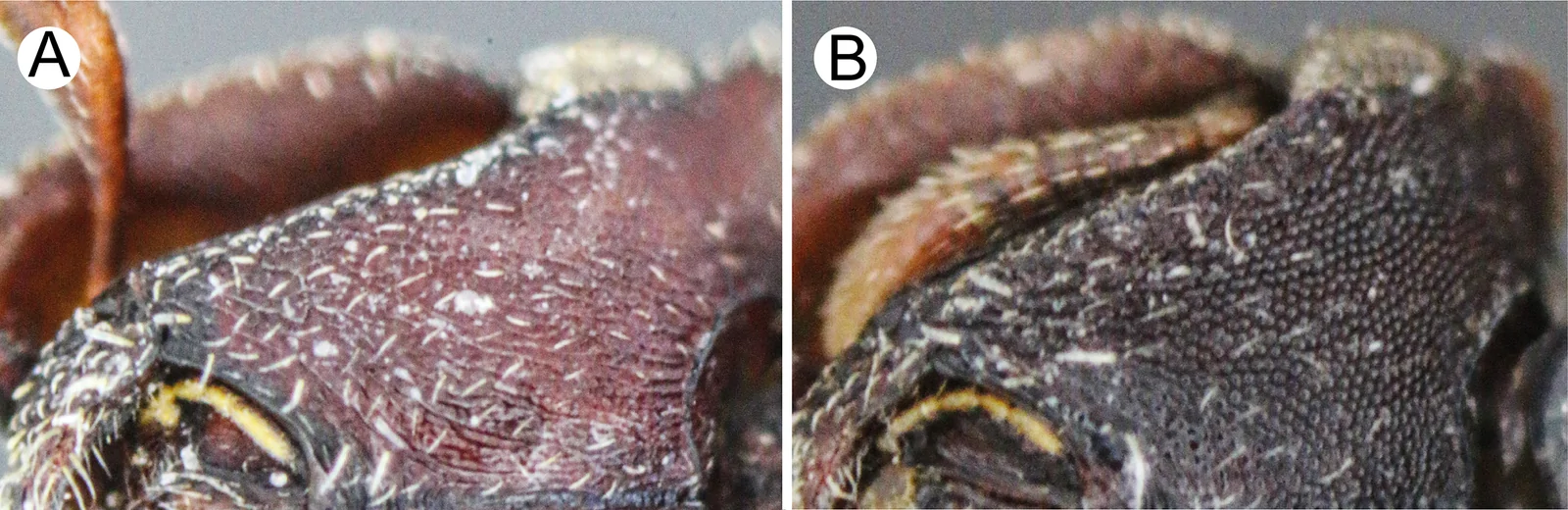
Head surface (ventral view). (A) Rugulose. *Cephalotes patellaris*. (B) Punctate. *Cephalotes pallidus*. Images by Adrian Troya.

**Figure 11:**
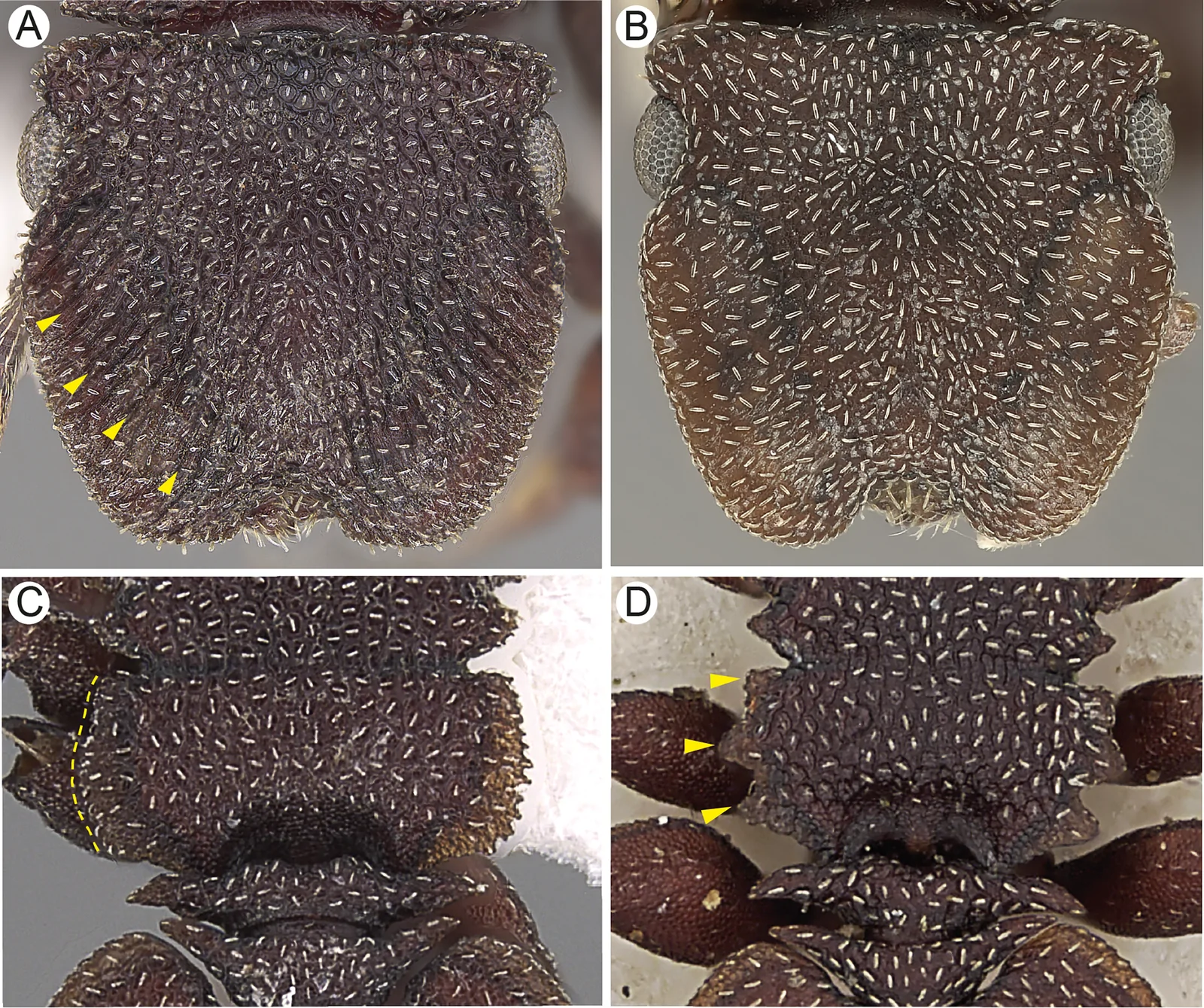
Frontal lobe (full-face view). (A) Longitudinally striate. *Cephalotes pallidus*. Image by Wade Lee. (B) Barely striate. *Cephalotes pallens*. Image by Shannon Hartman. Lateral pronotal margin (dorsal view). (C) Membranaceous, crenulate. *Cephalotes pallidus*. Image by Wade Lee. (D) Denticulate. *Cephalotes pallens*. Image by Ryan Perry.

**Figure 12:**
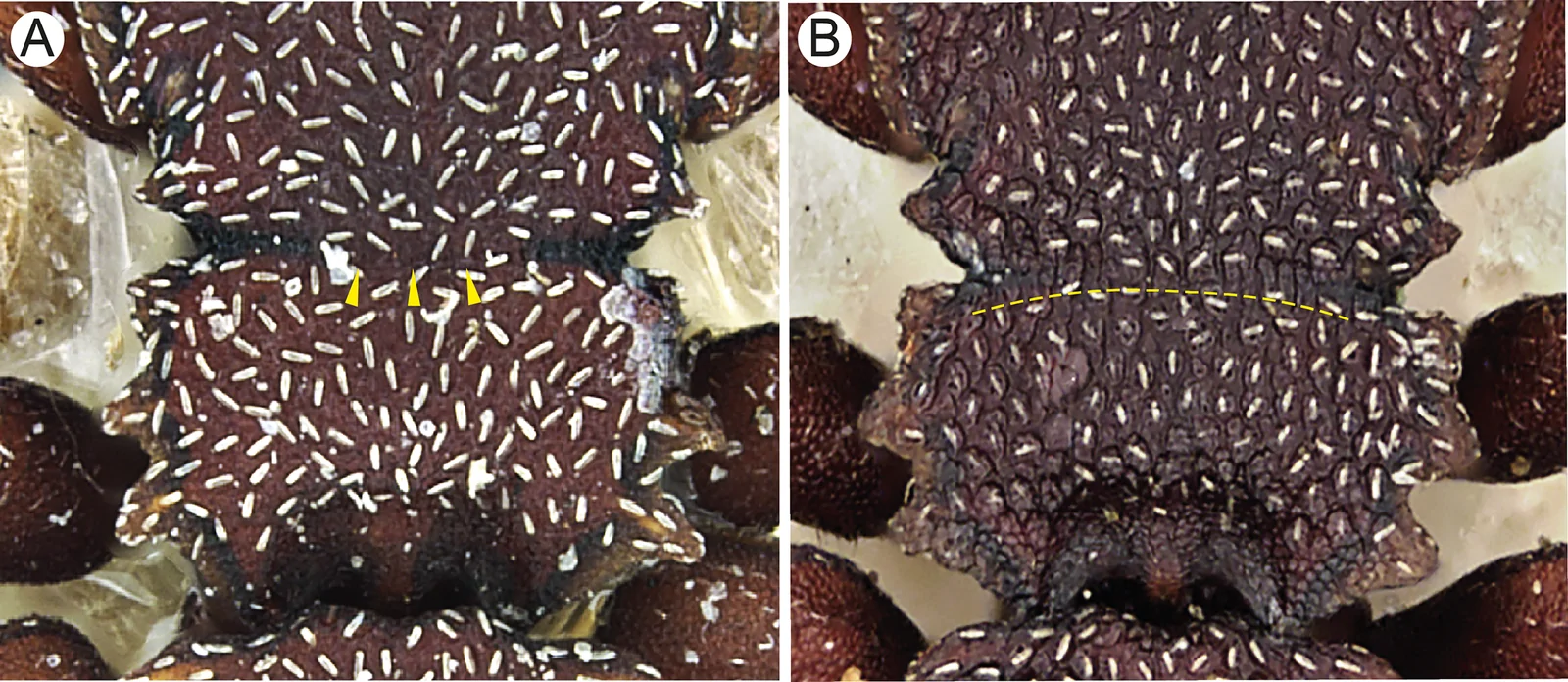
Propodeal groove (dorsal view). (A) Absent medially (yellow arrowheads). *Cephalotes porrasi*. Image by Zack Lieberman. (B) Continuous. *Cephalotes pallens*. Image by Ryan Perry.

**Figure 13:**
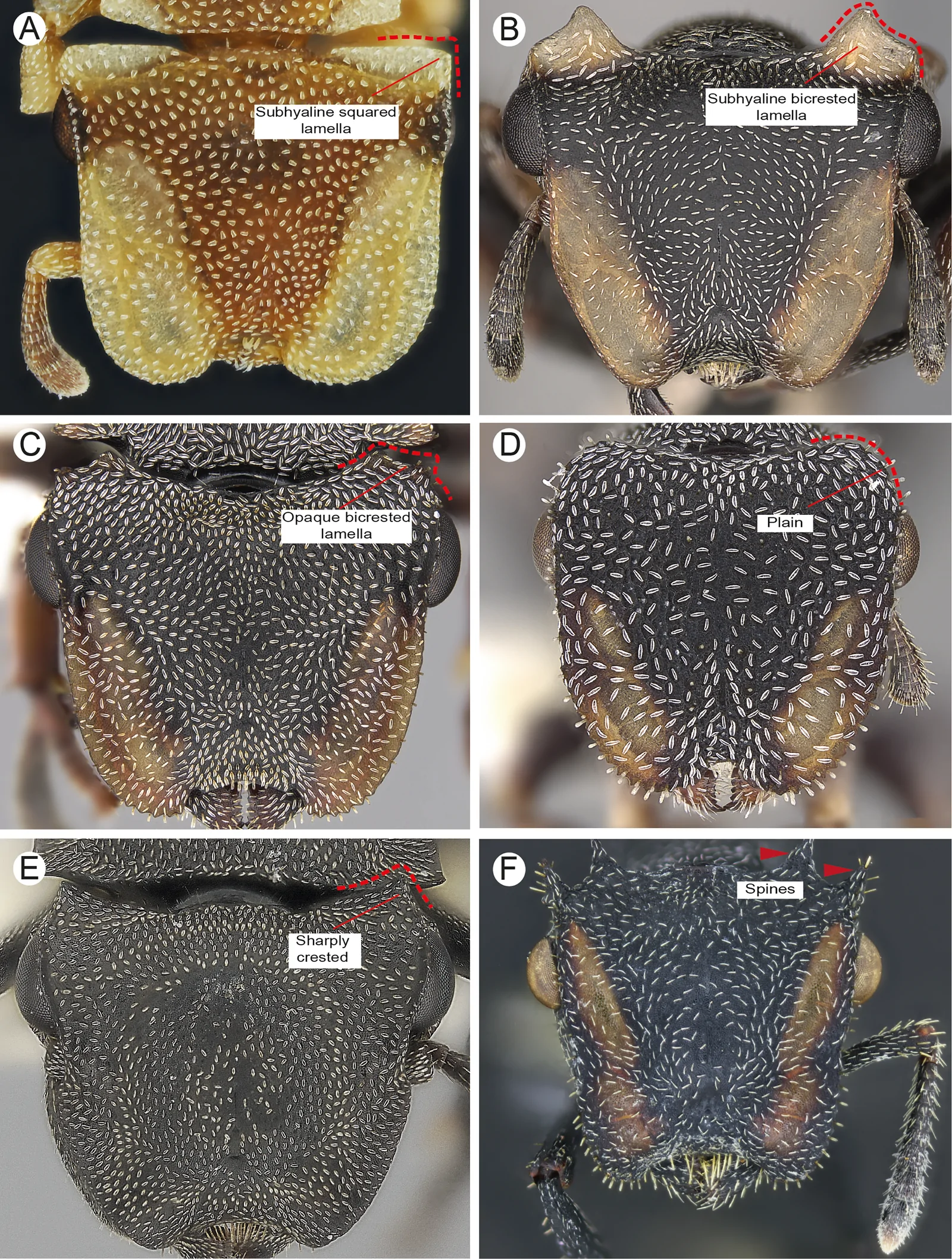
Posterolateral head margin (full-face view). (A) Subhyaline squared lamella. *Cephalotes esthelae*. (B) Subhyaline bicrested lamella. *Cephalotes spinosus*. Image by Ryan Perry. (C) Opaque lamella. *Cephalotes cordatus*. Image by Wade Lee. (D) Continuous. *Cephalotes setulifer*. Image by Wade Lee. (E) Sharply crested. *Cephalotes basalis* Image by Shannon Hartman. (F) Spiny. *Cephalotes sacha*. Image by Akayky Yumbay.

**Figure 14:**
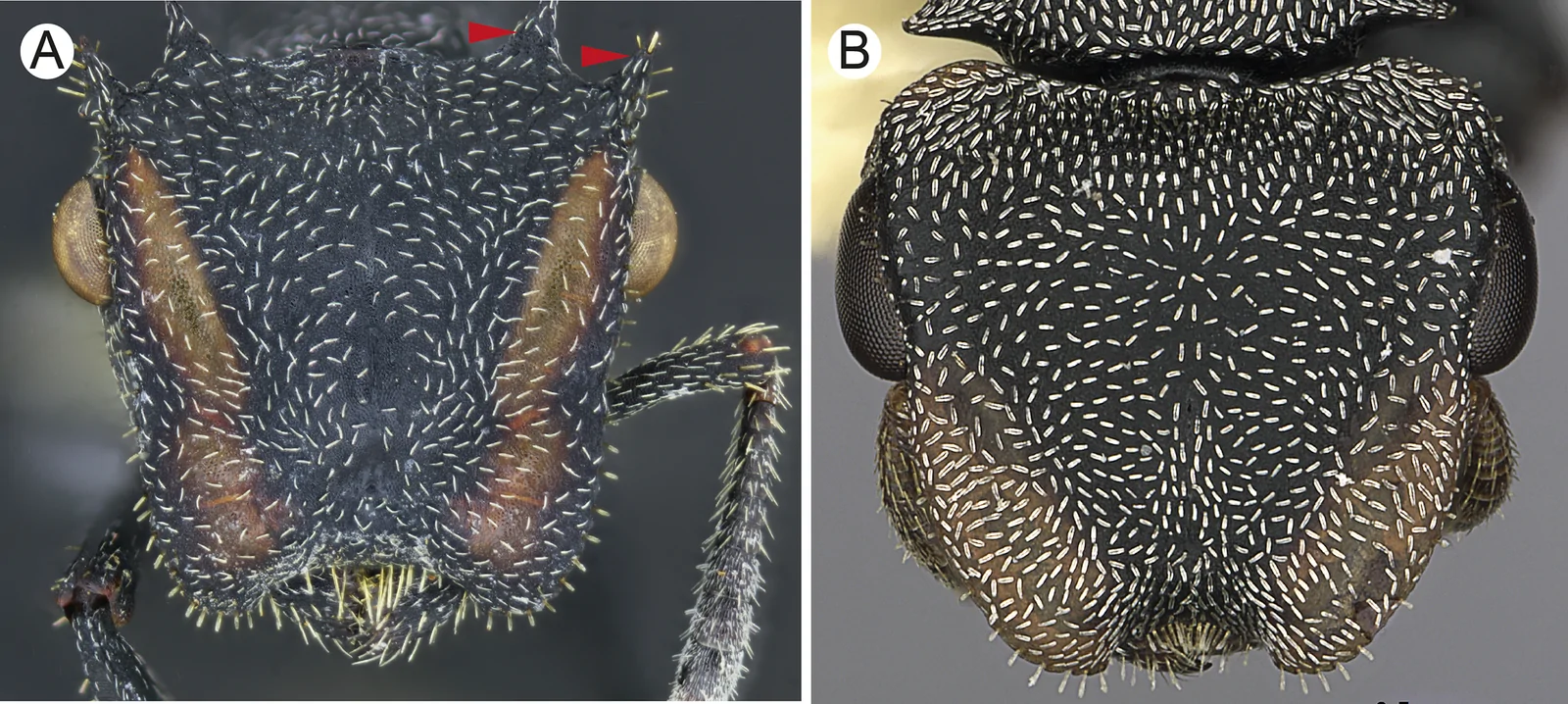
Posterolateral margin of head (full-face view). (A) Spiny. *Cephalotes sacha*. Image by Akayky Yumbay. (B) Without spines. *Cephalotes manni*. Image by Will Ericson.

**Figure 15:**
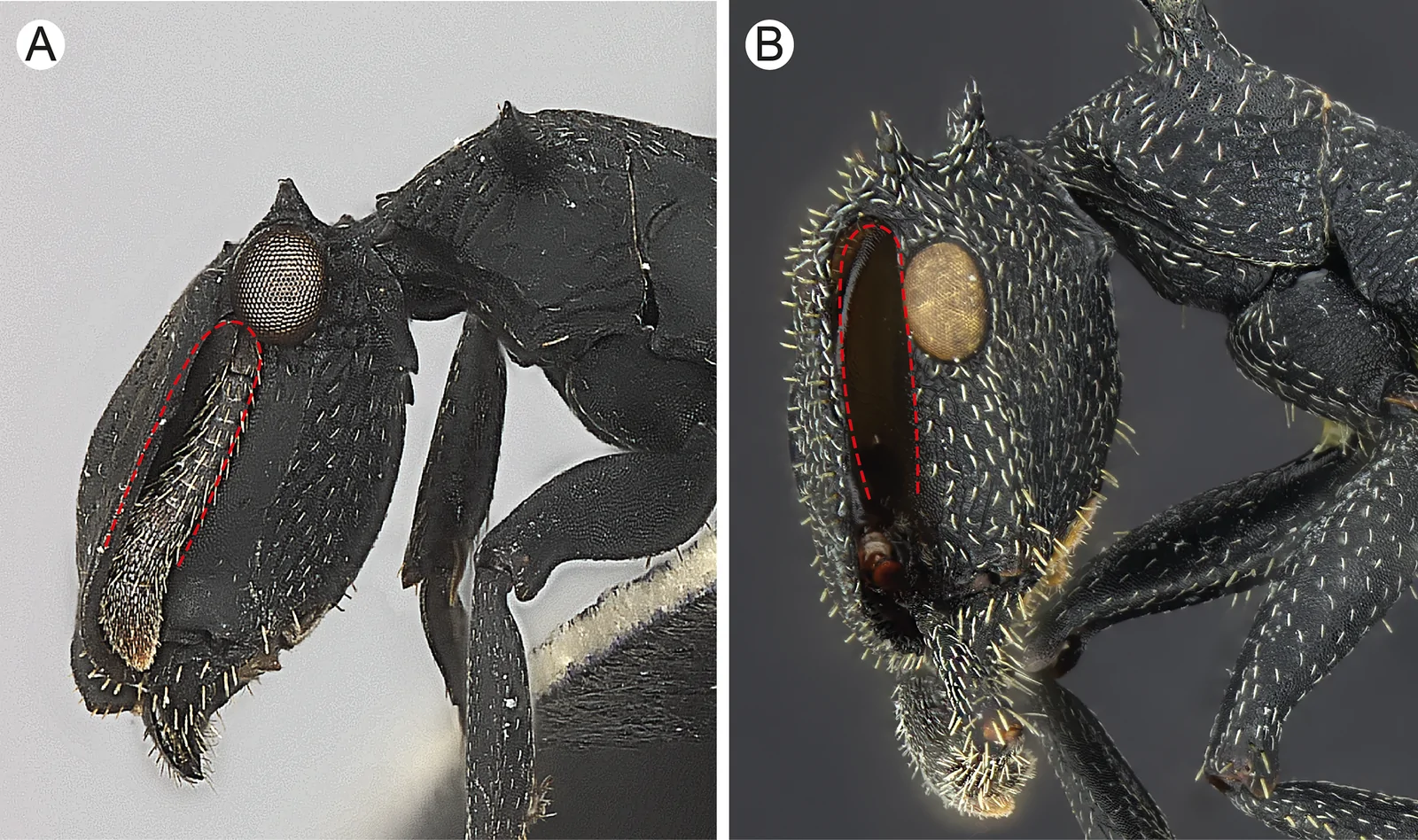
Eye placed (lateral view). (A) Behind scrobe. *Cephalotes opacus*. Image by Shannon Hartman. (B) Under scrobe. *Cephalotes sacha*. Image by Akayky Yumbay.

**Figure 16:**
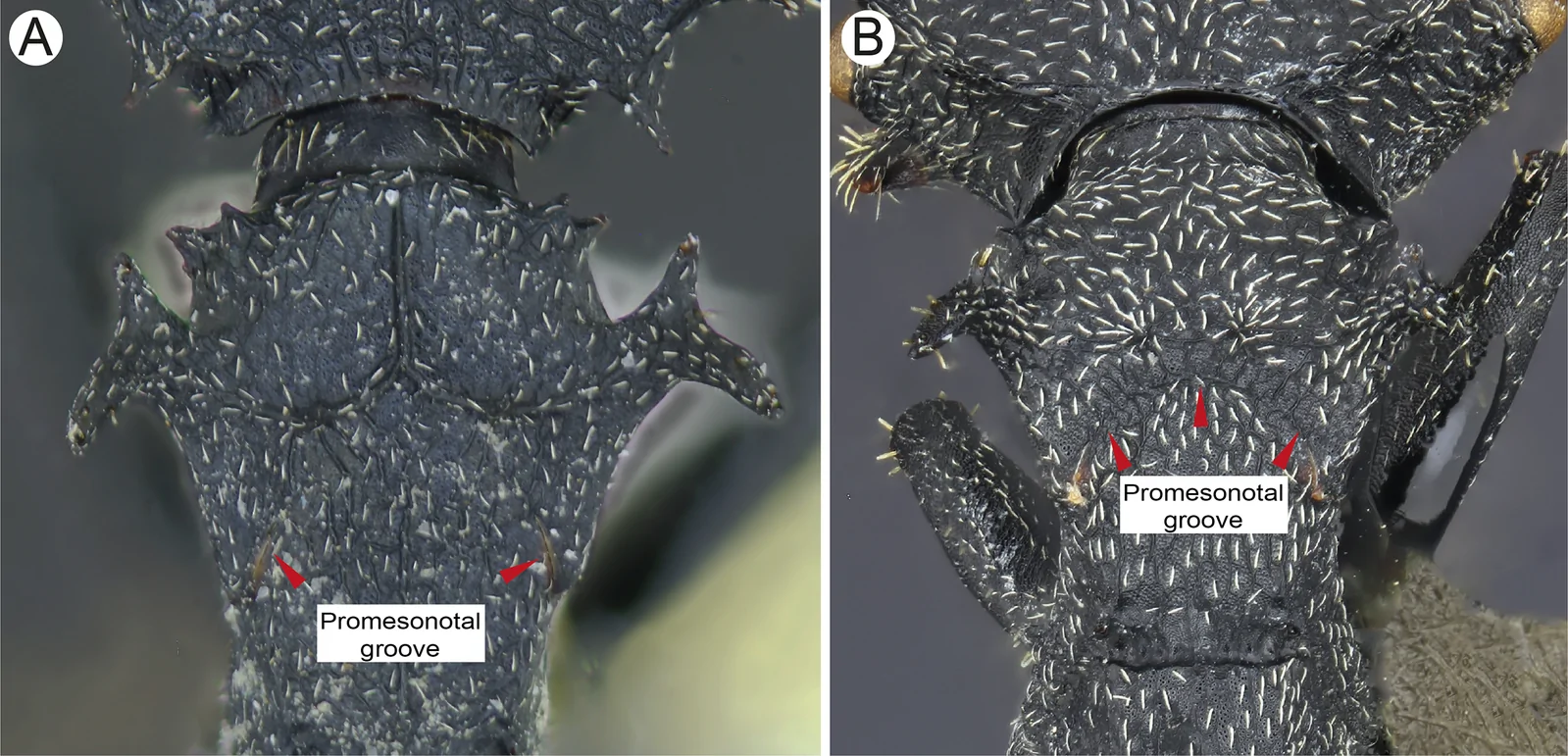
Pronotum (dorsal view). (A) With inverted Y-shaped carina. *Cephalotes serraticeps*. (B) Plain. *Cephalotes sacha*. Images by Akayky Yumbay.

**Figure 17:**
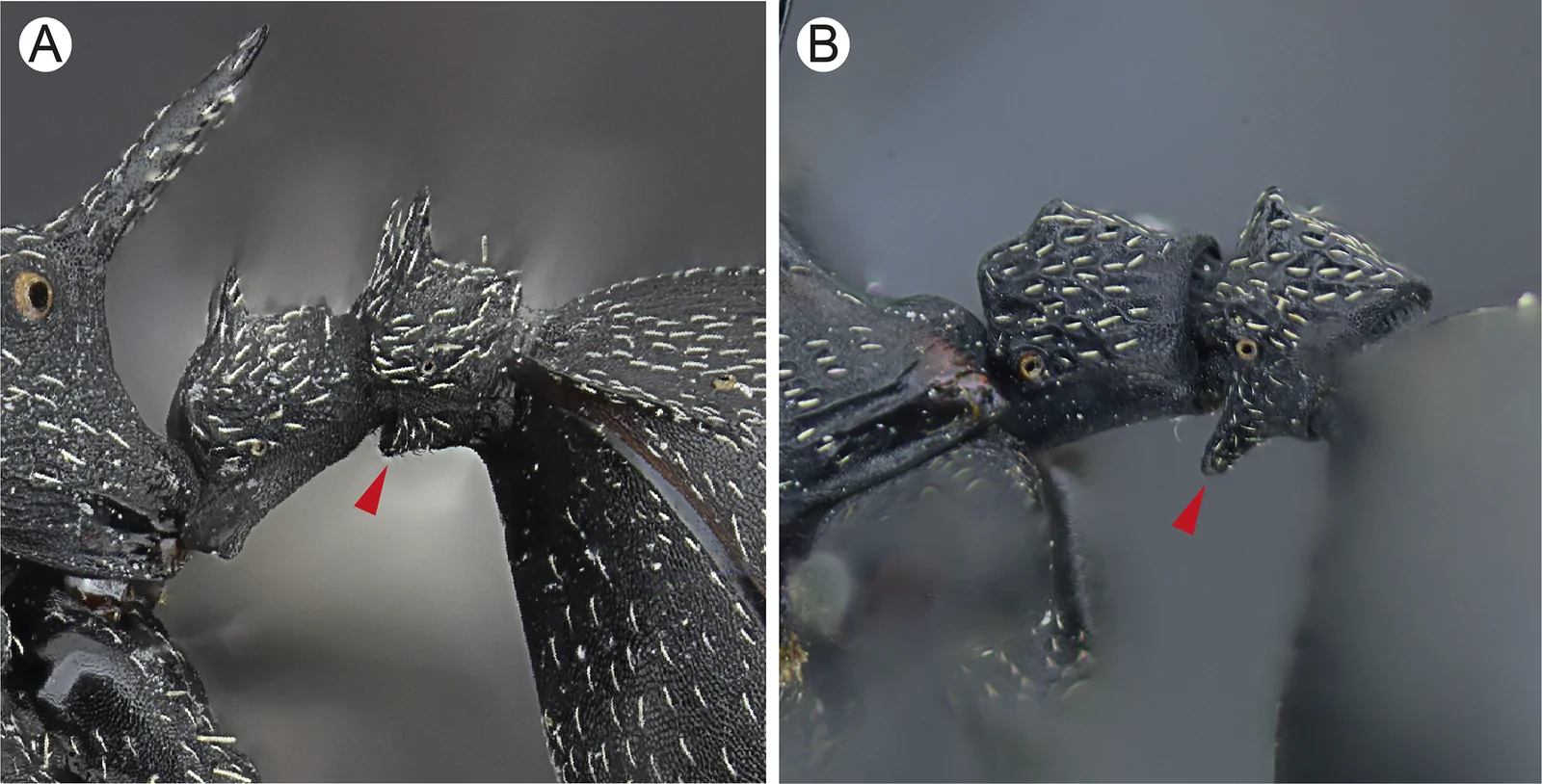
Subpostpetiolar process (lateral view). (A) Short (red arrowhead). *Cephalotes sacha*. (B) Elongated (red arrowhead). *Cephalotes atratus*. Images by Akayky Yumbay.

**Figure 18:**
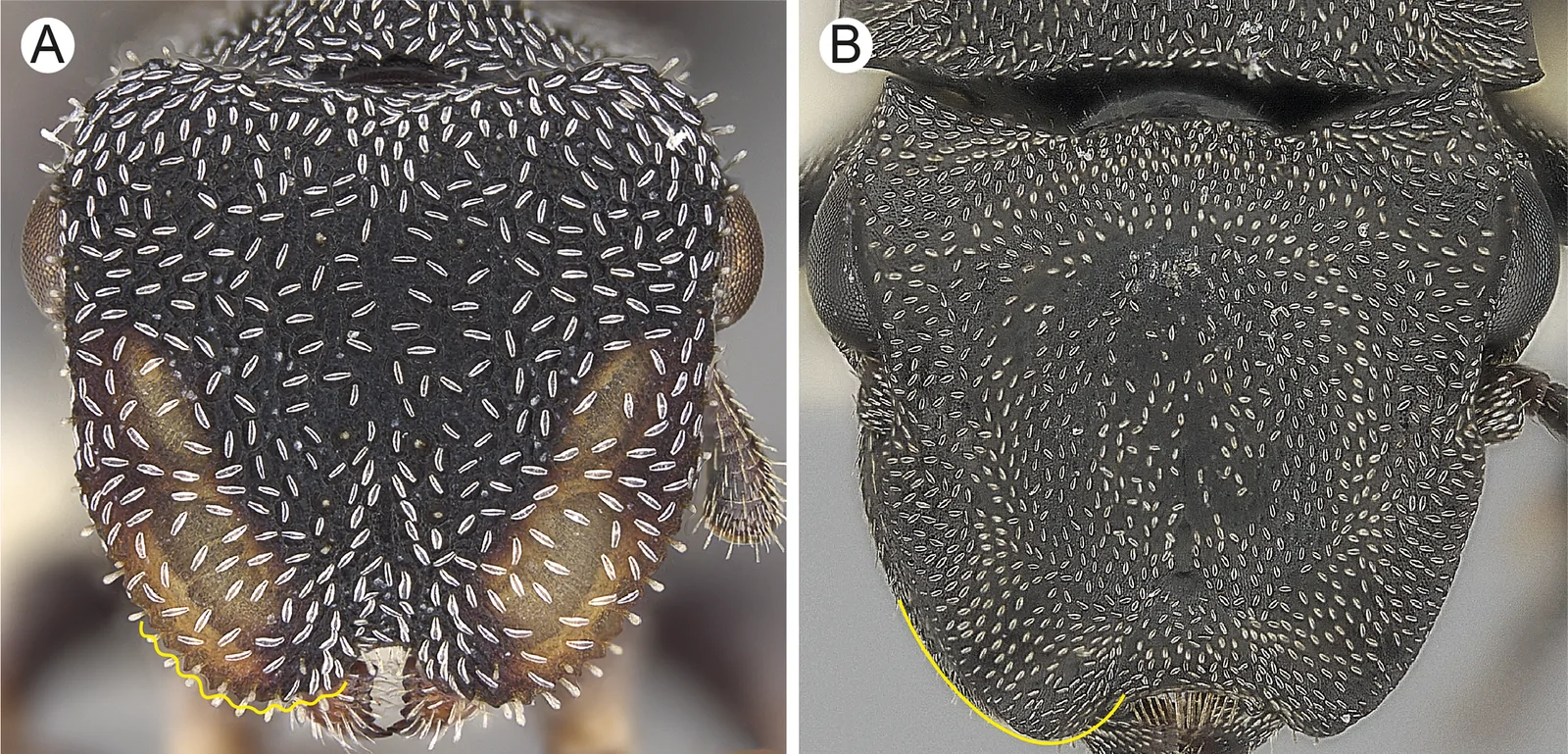
Anterolateral border of frontal carina (full-face view). (A) Crenulate. *Cephalotes setulifer*. Image by Wade Lee. (B) Plain. *Cephalotes basalis*. Image by Shannon Hartman.

**Figure 19:**
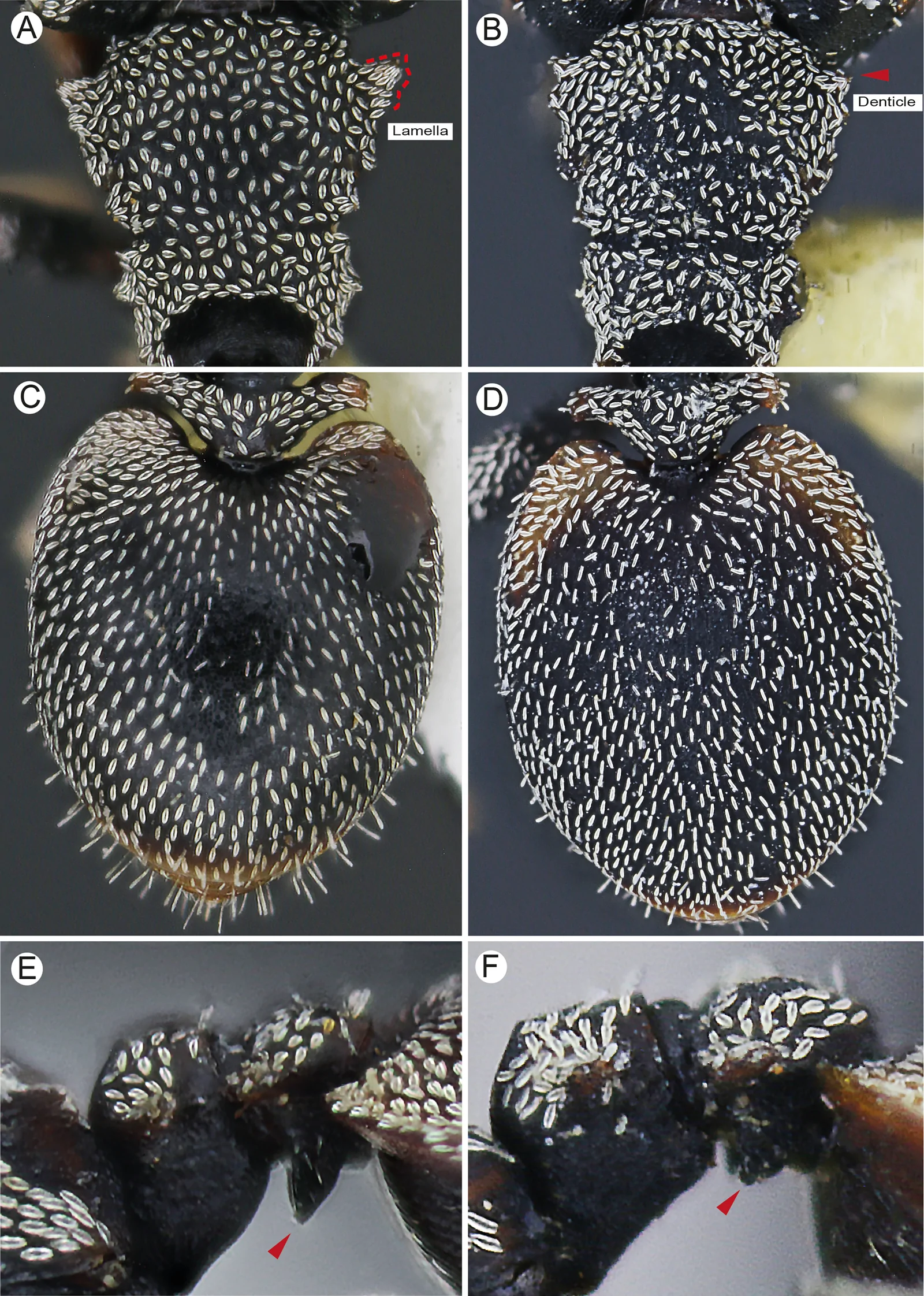
Lateral pronotal margin and gastric integument hue (dorsal view). (A) Lamellate. *Cephalotes trichophorus*. (B) Denticulate. *Cephalotes setulifer*. (C) Shiny. *Cephalotes trichophorus*. (D) Opaque. *Cephalotes setulifer*. Subpostpetiolar process (lateral view). (E) Elongated (red arrowhead). *Cephalotes trichophorus* (F) Short (red arrowhead). *Cephalotes setulifer*. Images (A), (B) by Akayky Yumbay; (C)–(F) by Adrian Troya.

**Figure 20:**
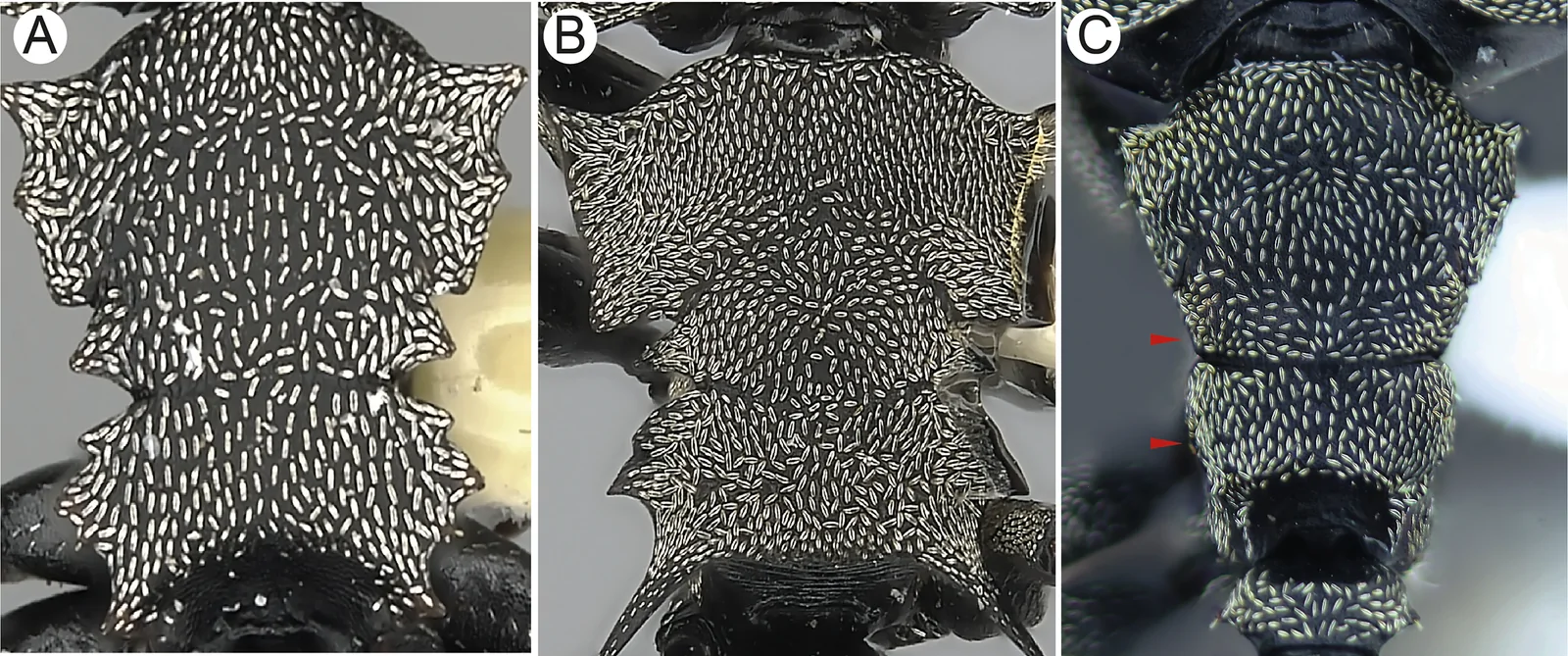
Lateral mesonotal and propodeal margins (dorsal view). (A) Denticulate. *Cephalotes manni*. Image by Will Ericson. (B) Spiny. *Cephalotes inca*. Image by Shannon Hartman. (C) Plain (red arrowheads). *Cephalotes solidus*. Image by Akayky Yumbay.

**Figure 21:**
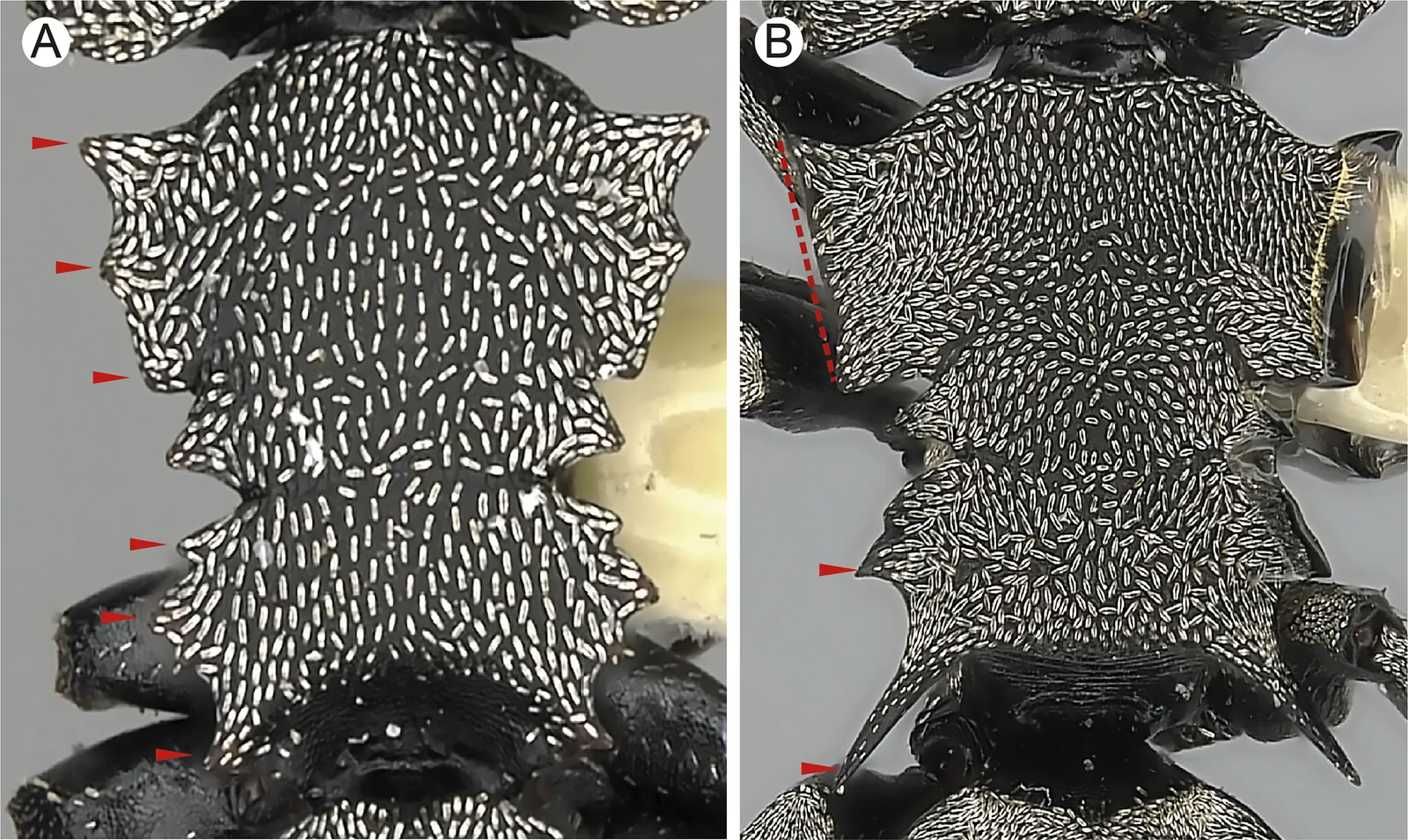
Lateral pronotal and propodeal margins (dorsal view). (A) Denticulate (red arrowheads). *Cephalotes manni*. Image by Will Ericson. (B) Lamellate pronotum (red dotted line), spiny propodeum (red arrowheads). *Cephalotes inca*. Image by Shannon Hartman.

**Figure 22:**
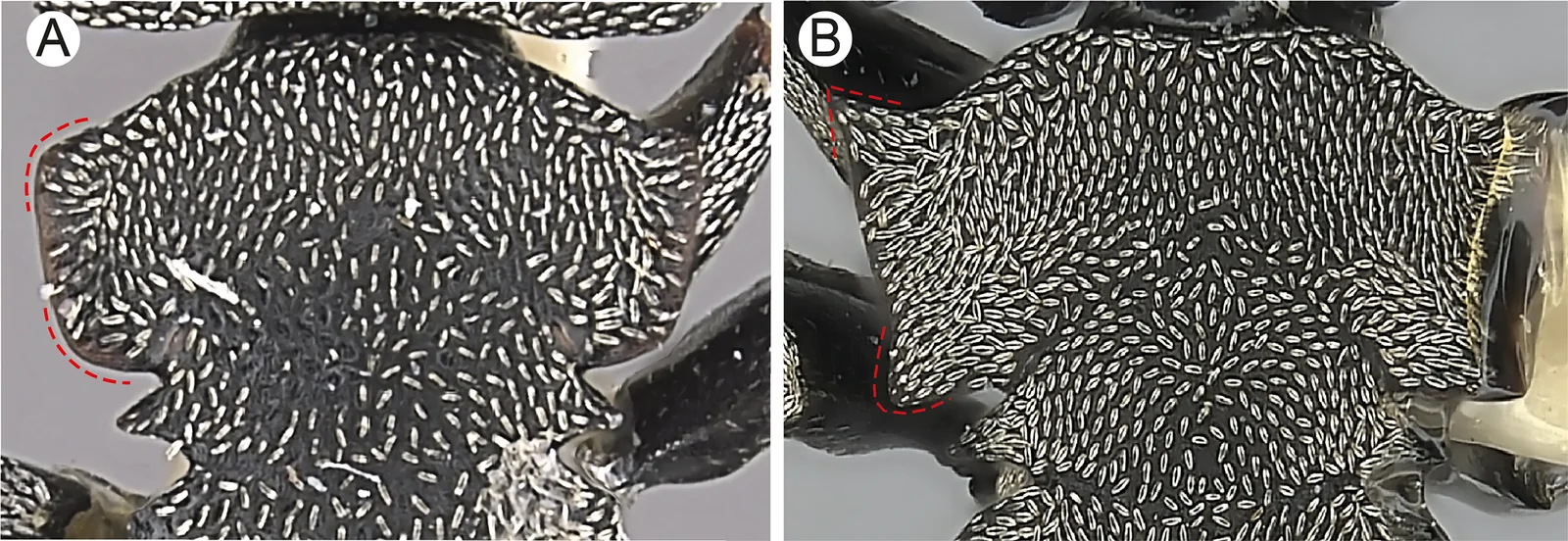
Humeral lamellae (dorsal view). (A) Subacute to rounded anteriorly, rounded posteriorly (red dotted lines). *Cephalotes ramiphilus*. Image by Will Ericson. (B) With spine anteriorly, acute posteriorly (red dotted lines). *Cephalotes inca*. Image by Shannon Hartman.

**Figure 23:**
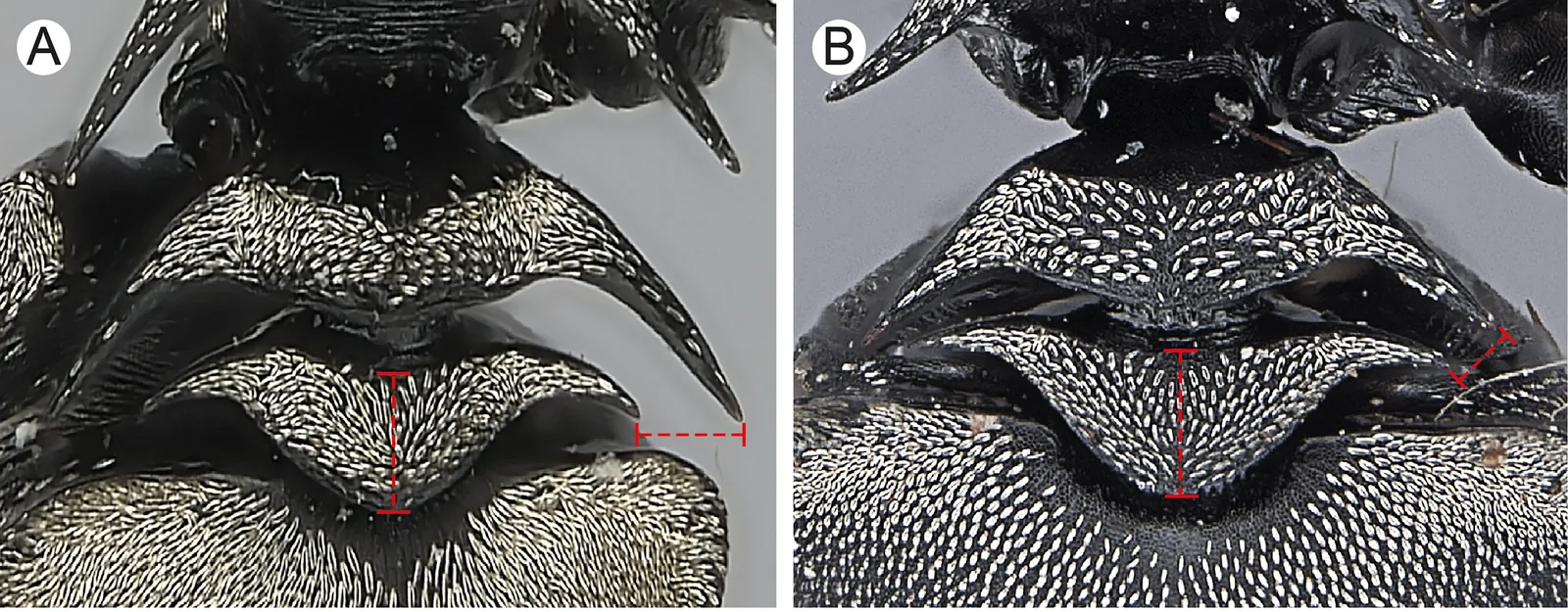
Distance from petiolar spinal tip to postpetiolar spinal tip as compared to mid postpetiolar length. (A) Subequal. *Cephalotes inca*. Image by Shannon Hartman. (B) Shorter. *Cephalotes basalis*. Image by Alexandra Westrich.

**Figure 24:**
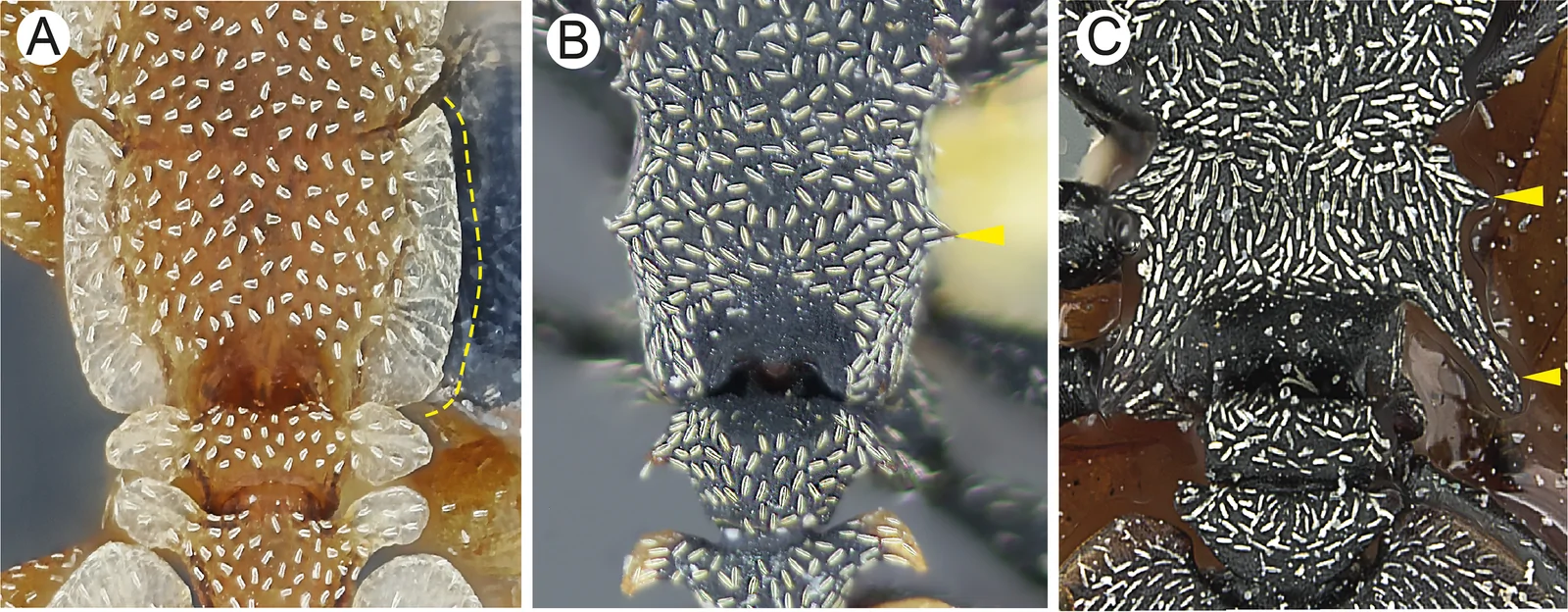
Lateral propodeal margin (dorsal view). (A) Lamellate. *Cephalotes esthelae*. Image by Elian Ilguan. (B) Denticulate (yellow arrowhead). *Cephalotes ecuadorialis*. Image by Akayky Yumbay. (C). Spiny (yellow arrowheads). *Cephalotes pusillus*. Image by Zack Lieberman.

**Figure 25:**
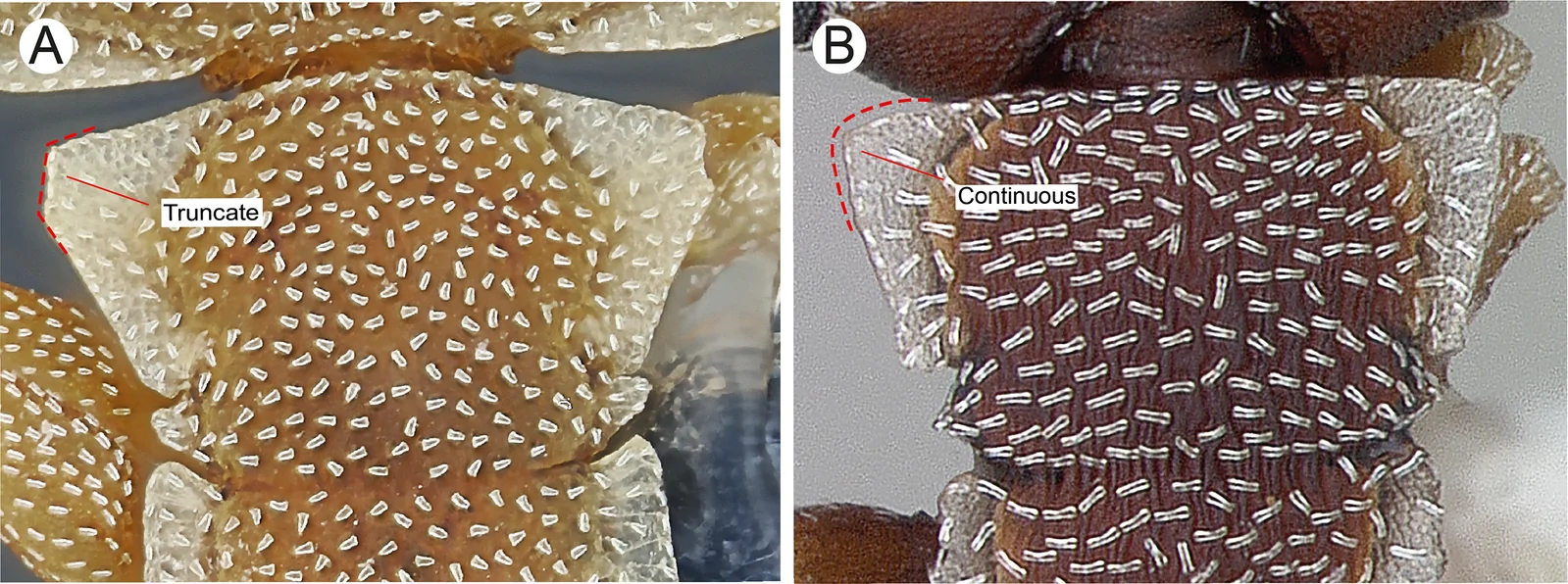
Pronotal lamella (dorsal view). (A) Truncate (red dotted line). *Cephalotes esthelae*. Image by Elian Ilguan. (B) Continuous (red dotted line). *Cephalotes maculatus*. Image by April Nobile.

**Figure 26:**
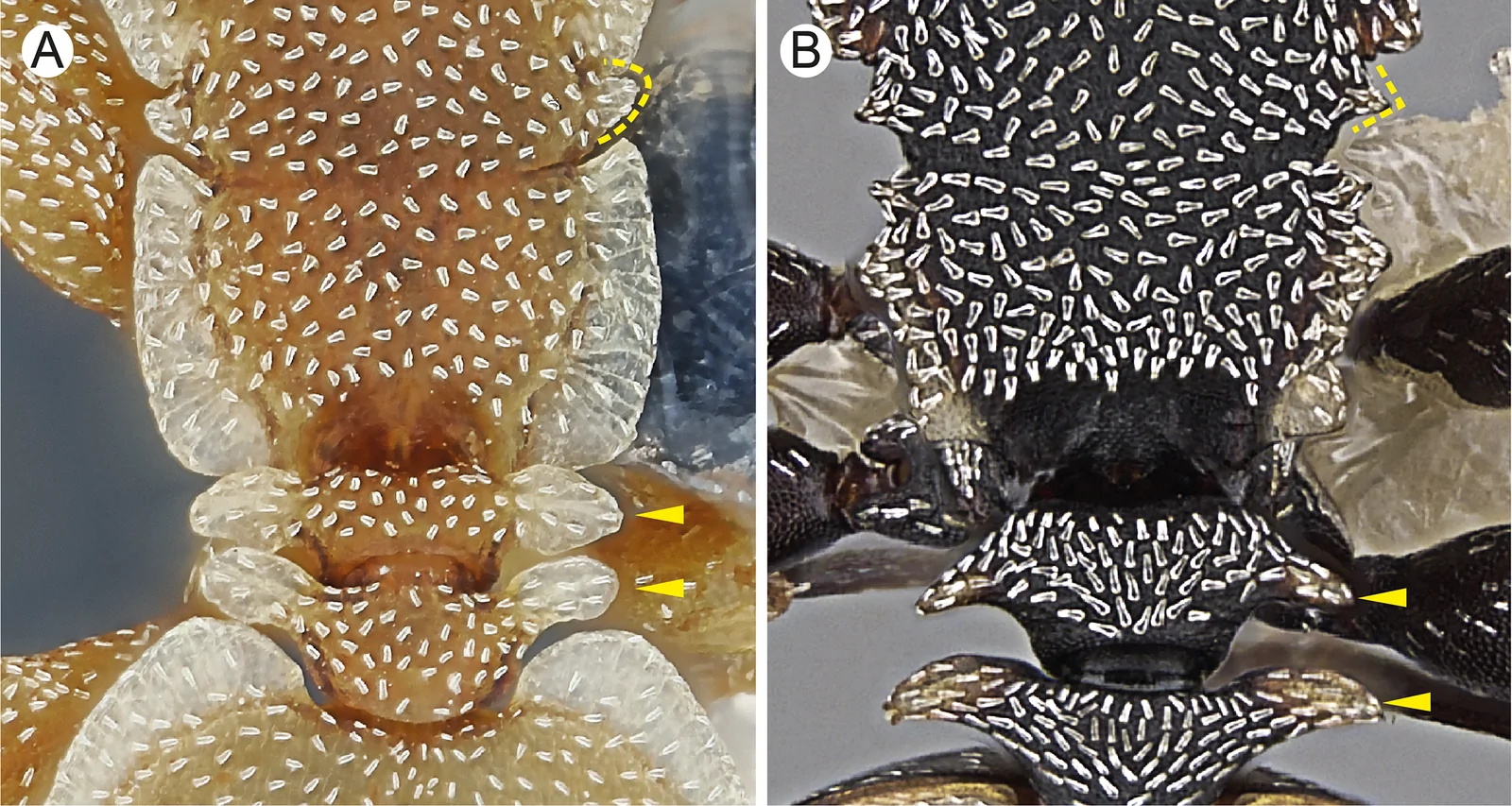
Mesonotal and petiolar lateral projections (dorsal view). (A) Lamellate (yellow dotted line and arrowheads). *Cephalotes esthelae*. Image by Elian Ilguan. (B) Denticulate (yellow dotted line and arrowheads). *Cephalotes scutulatus*. Image by Wade Lee.

**Figure 27:**
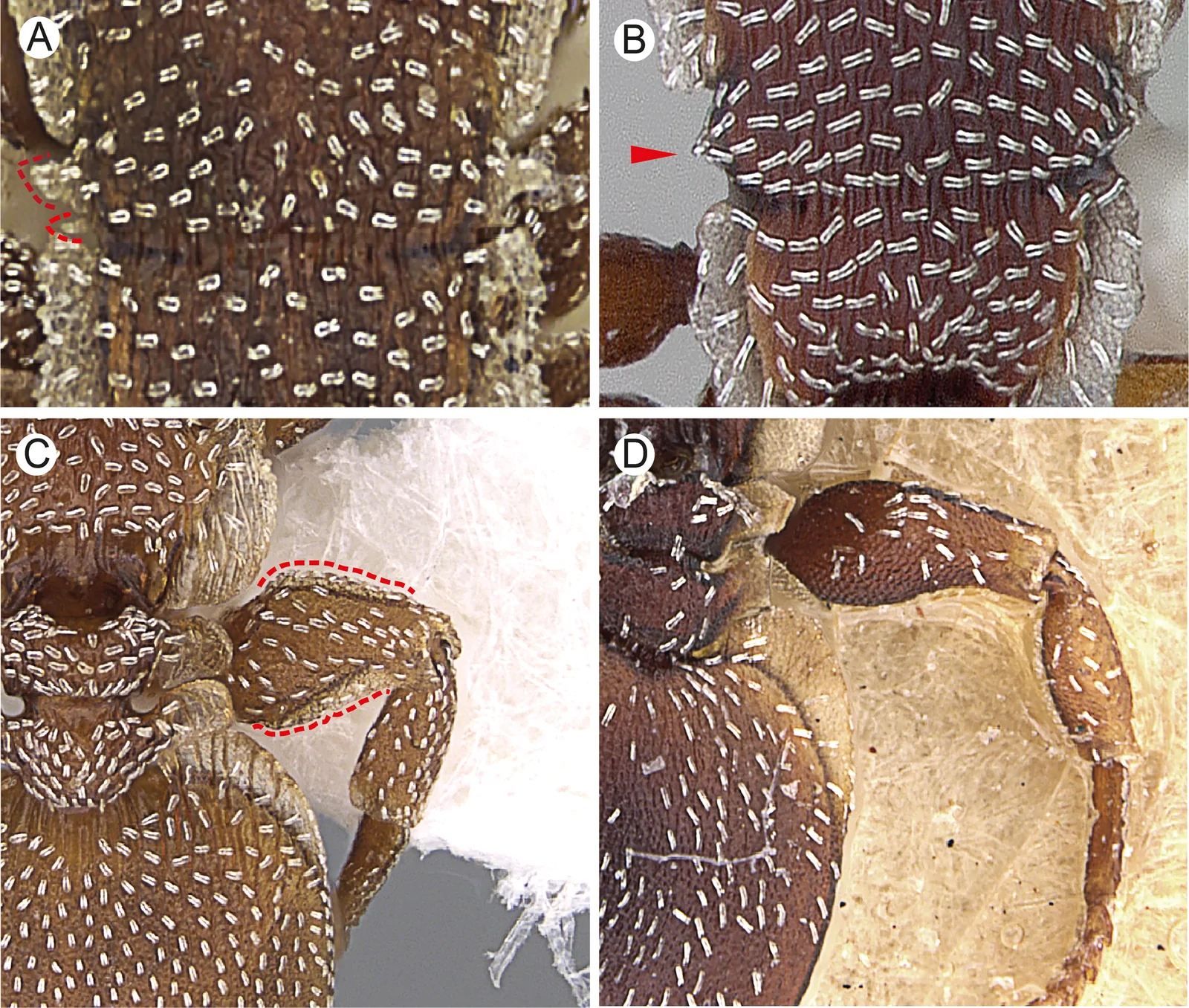
Lateral mesonotal margin (dorsal view). (A) Lamellate (red dotted lines). *Cephalotes grandinosus*. Image by Ryan Perry. (B) Denticulate (red arrowhead). *Cephalotes maculatus*. Image by April Nobile. Hind femur (dorsal or ventral view). (C) Lamellate (red dotted lines). *Cephalotes persimplex*. Image by Wade Lee. (D) Without lamella. *Cephalotes maculatus*. Image by Will Ericson.

**Figure 28:**
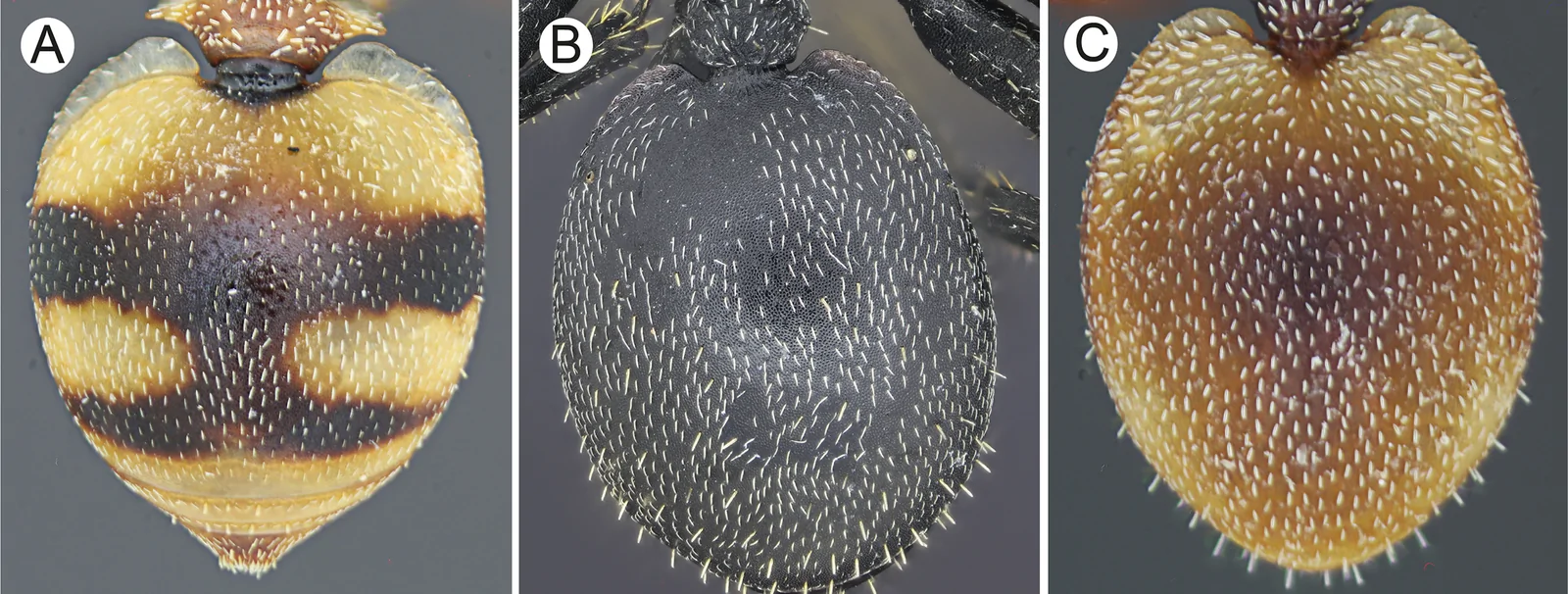
Gaster (dorsal view). (A) Spotted. *Cephalotes umbraculatus*. (B) Concolored. *Cephalotes sacha*. (C) Heterocolored. *Cephalotes peruviensis*. Images by Akayky Yumbay.

**Figure 29:**
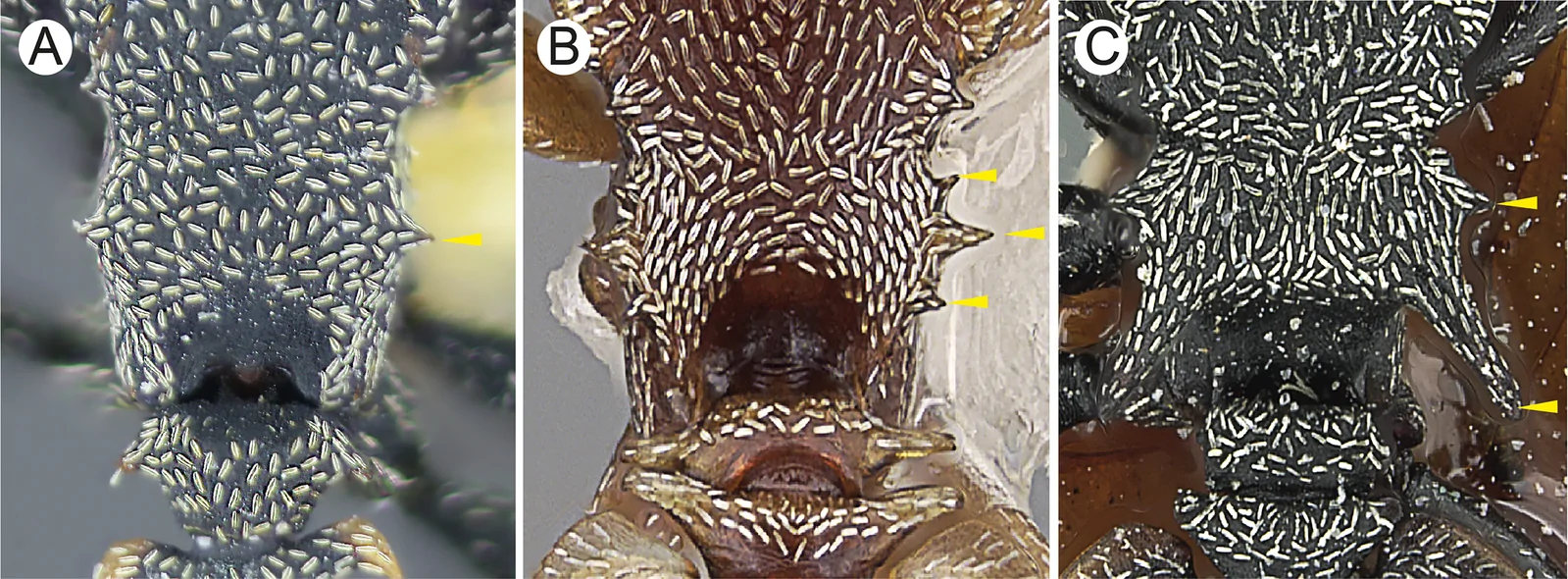
Lateral propodeal margin (dorsal view). (A) Denticulate (yellow arrowhead). *Cephalotes ecuadorialis*. Image by Akayky Yumbay. (B) Tridenticualate (yellow arrowheads). *Cephalotes dentidorsum*. Image by Wade Lee. (C) Spiny (yellow arrowheads). *Cephalotes pusillus*. Image by Zack Lieberman.

**Figure 30:**
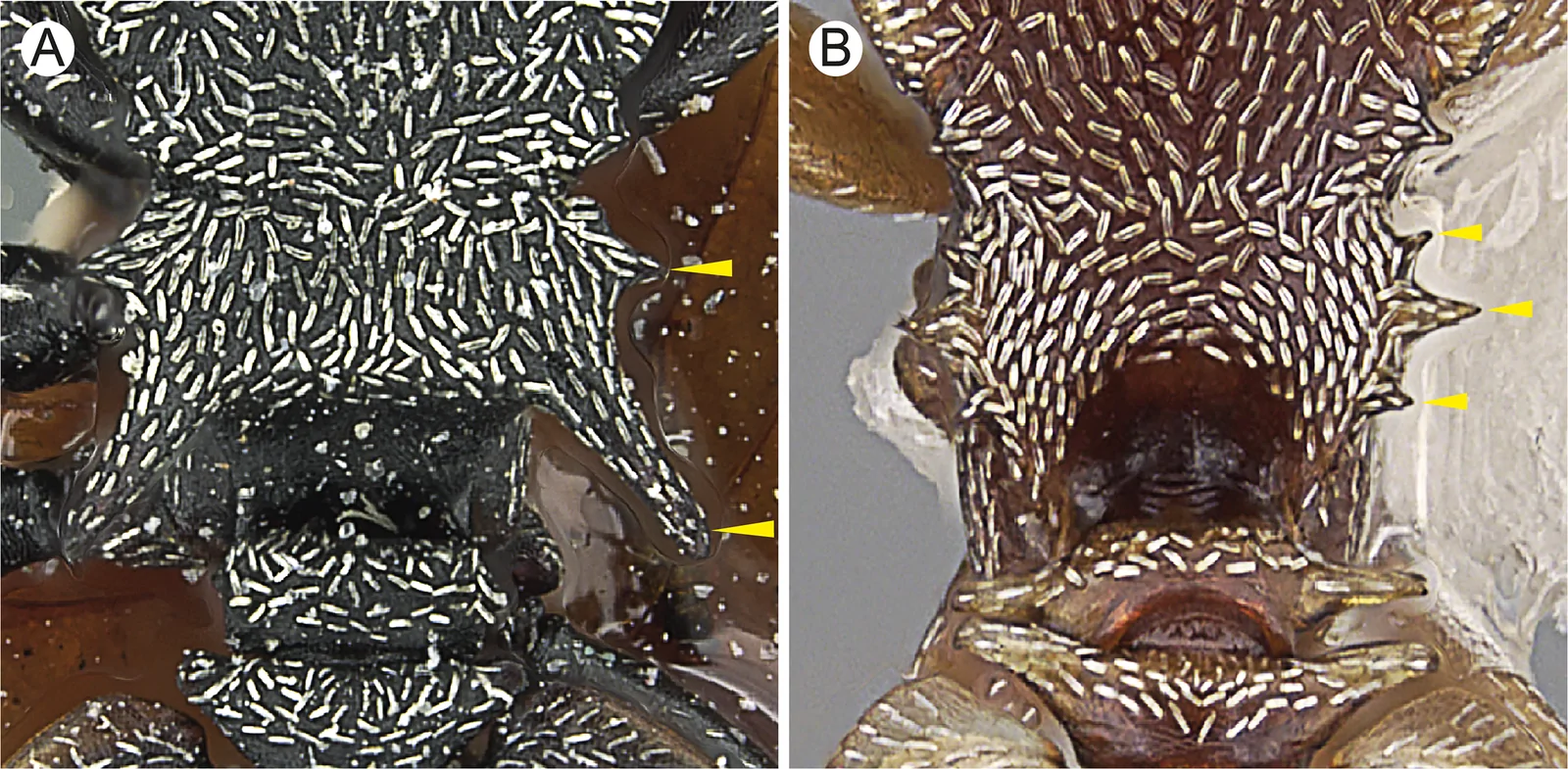
Lateral propodeal margin (dorsal view). (A) Bispinose (yellow arrowheads). *Cephalotes pusillus*.Image by Zack Lieberman. (B) Tridenticulate (yellow arrowheads). *Cephalotes dentidorsum*. Image by Wade Lee.

**Figure 31:**
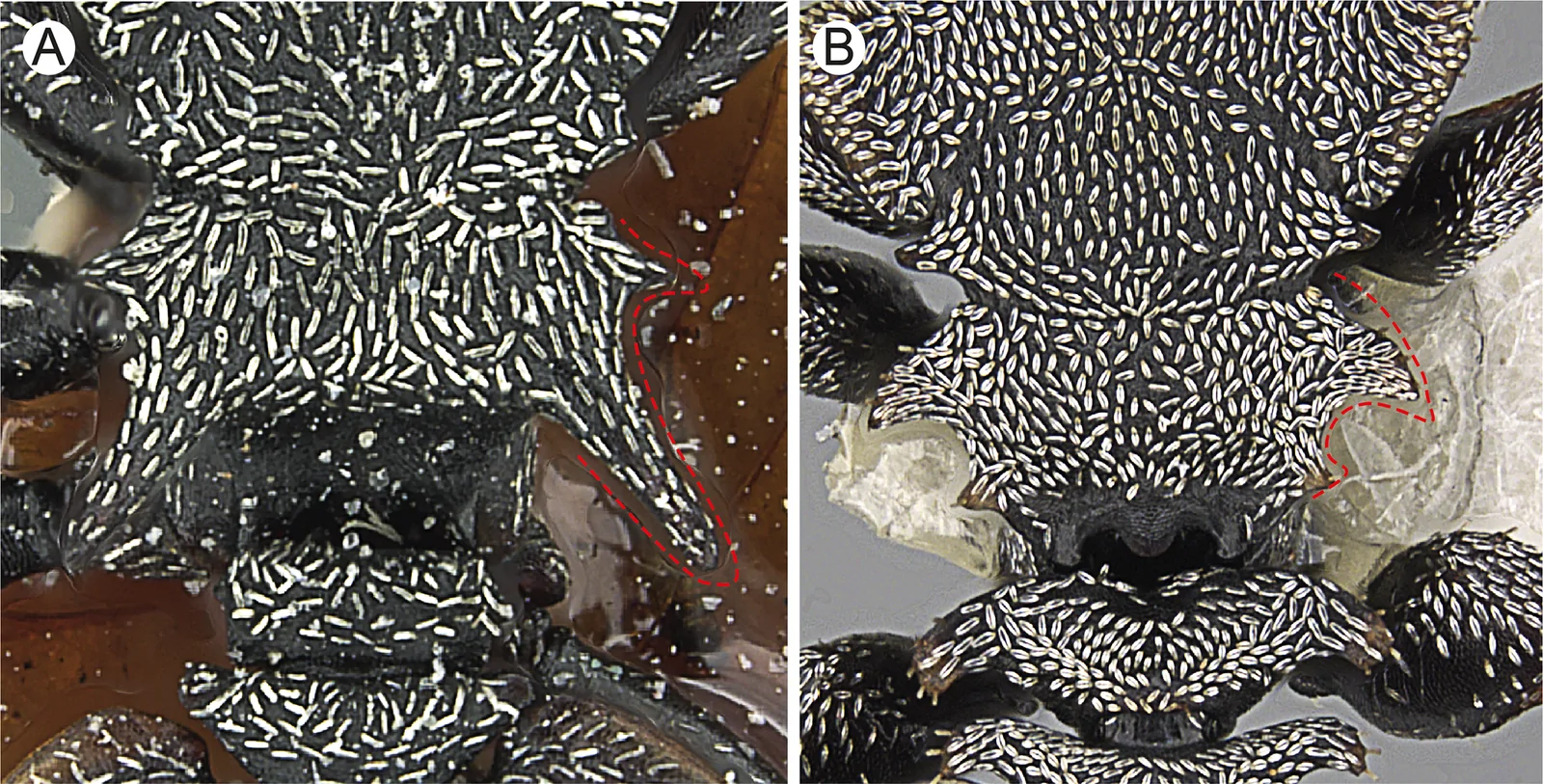
Posterior propodeal spine as compared to anterior spine (dorsal view). (A) Longer (red dotted line). *Cephalotes pusillus*. Image by Zack Lieberman. (B) Shorter (red dotted line). *Cephalotes cordatus*. Image by Wade Lee.

**Figure 32:**
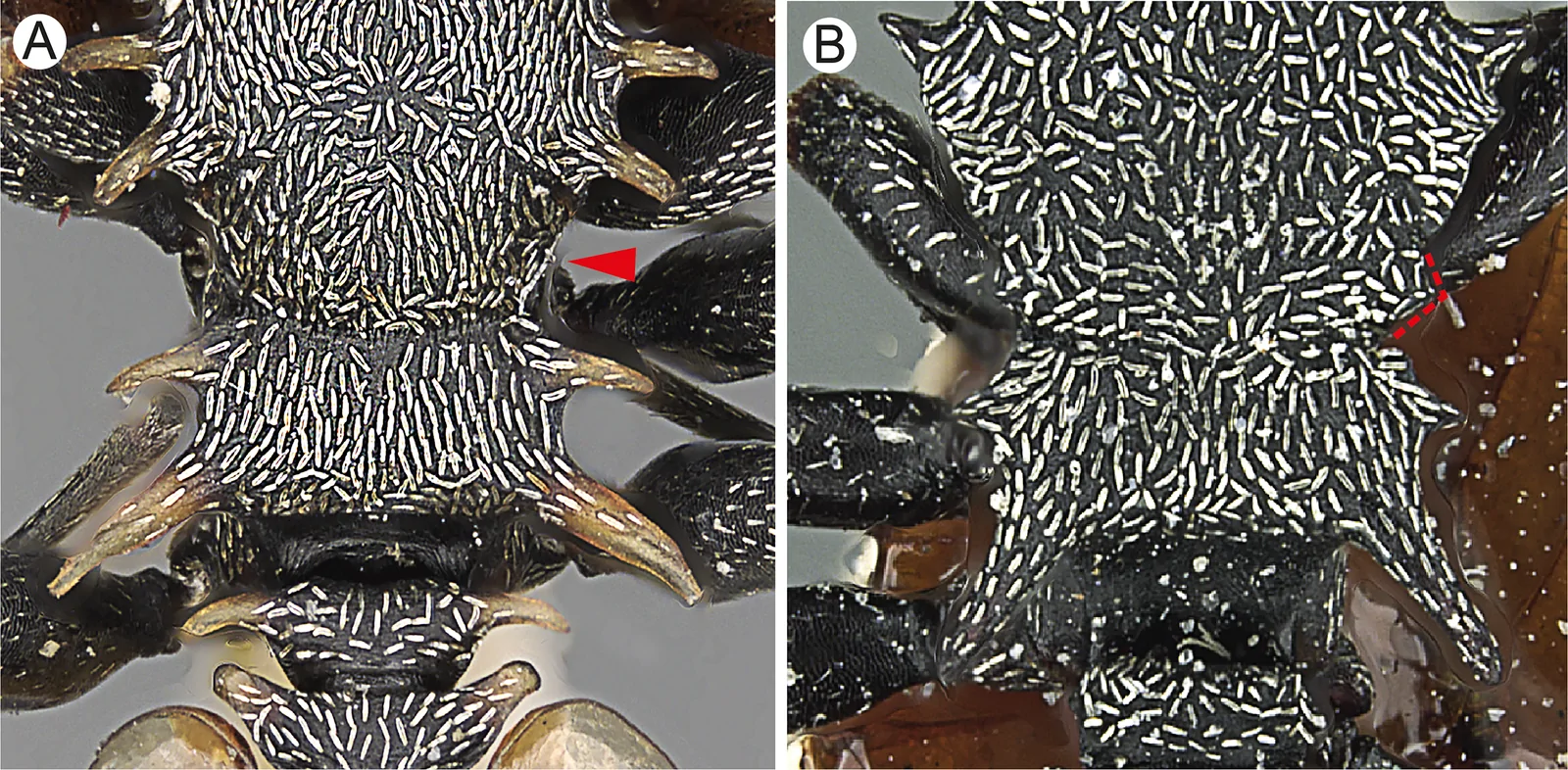
Lateral mesonotal margin (dorsal view). (A) Plain (red arrowhead). *Cephalotes spinosus*. Image by Ryan Perry. (B) Denticulate (red dotted line). *Cephalotes pusillus*. Image by Zack Lieberman.

**Figure 33:**
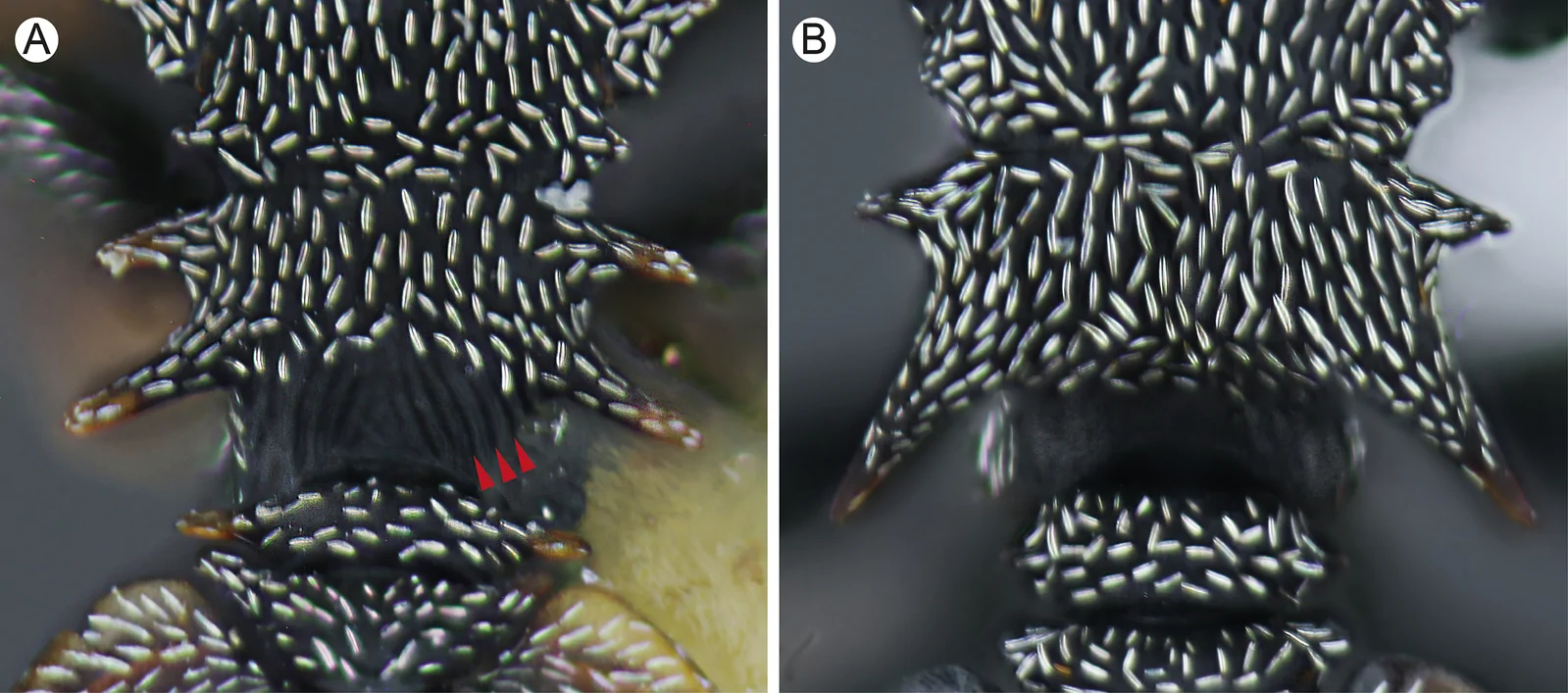
Declivous face of propodeum (dorsal view). (A) Striate (red arrowheads). *Cephalotes minutus*. (B) Plain or feebly punctate. *Cephalotes pusillus*. Images by Akayky Yumbay.

**Figure 34:**
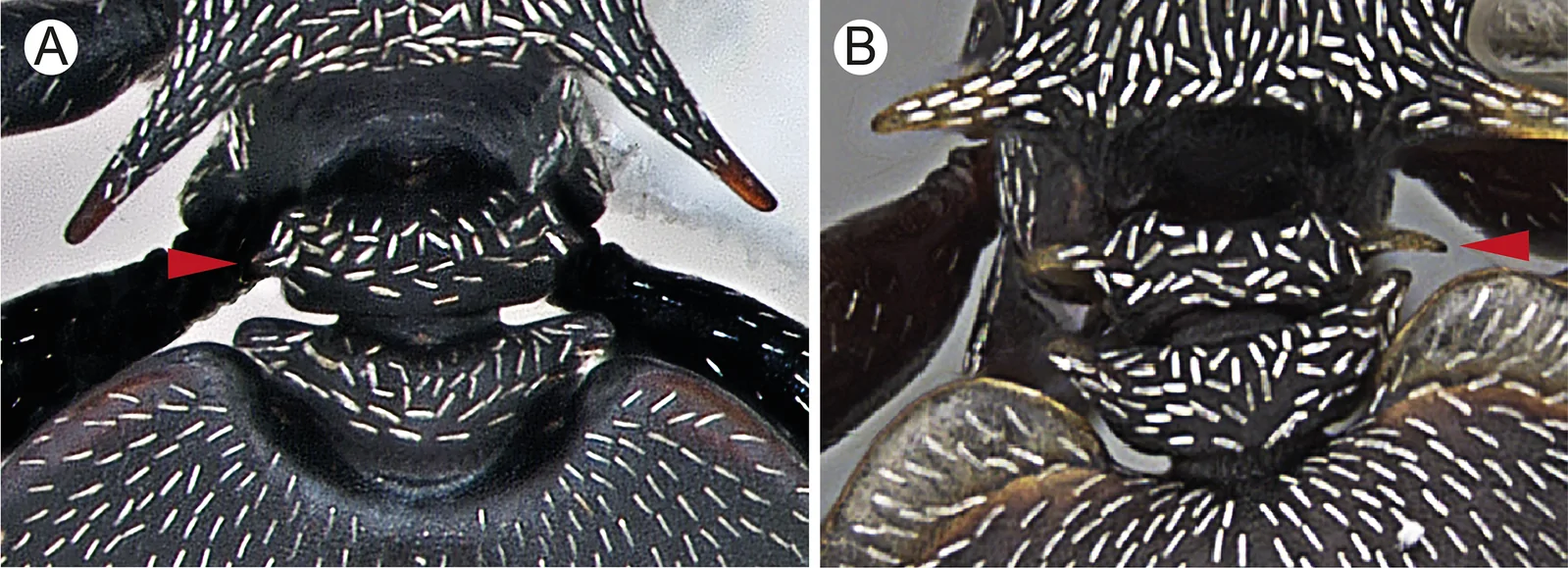
Lateral margin of petiole (dorsal view). (A) Denticulate (red arrowhead). *Cephalotes pusillus*. Image by April Nobile. (B) Spiny (red arrowhead). *Cephalotes laminatus*. Image by Wade Lee.

**Figure 35:**
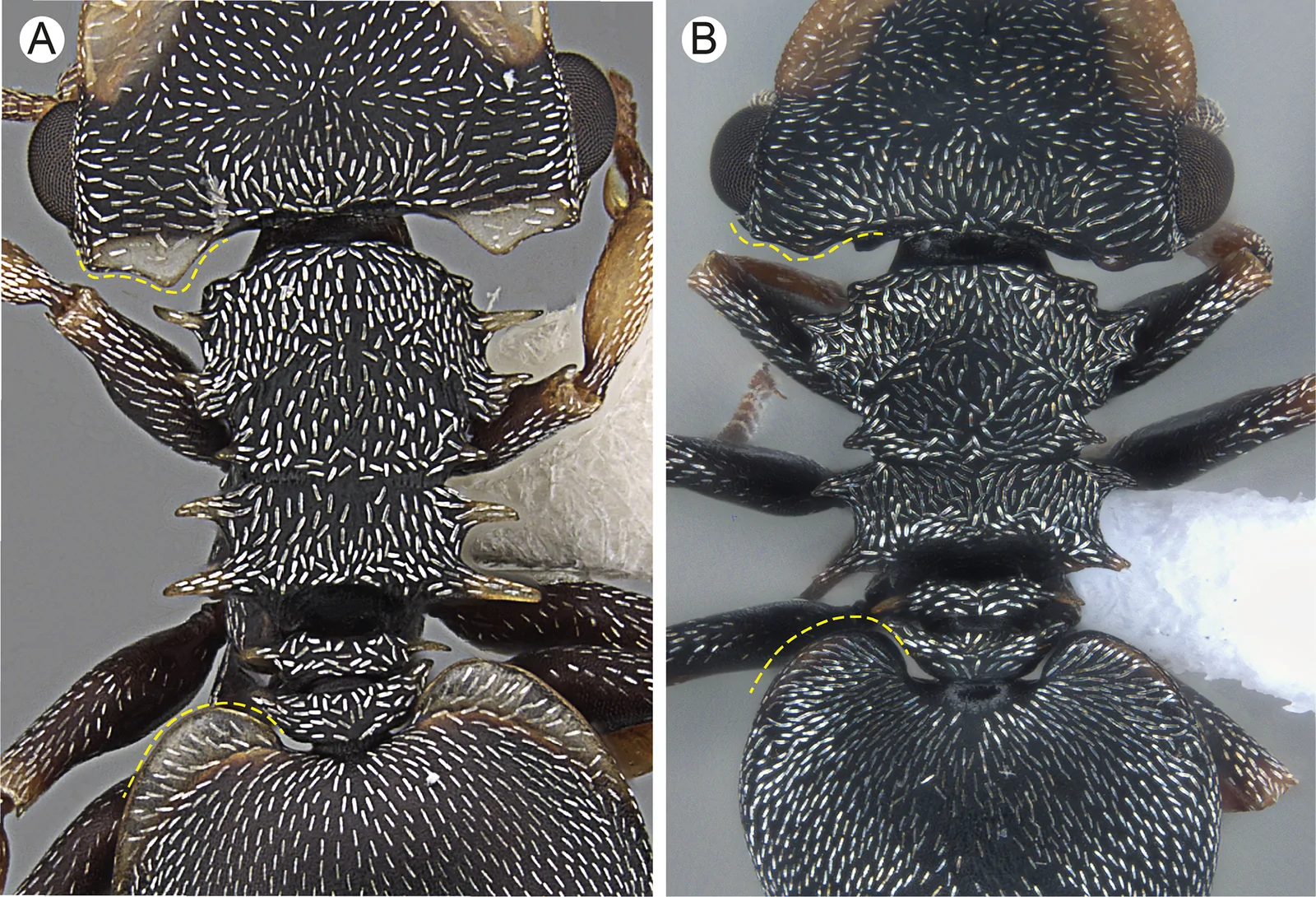
Head and gastric lamellae (dorsal view). (A) Subhyaline (yellow dotted lines). *Cephalotes laminatus*. Image by Wade Lee. (B) Concolorous to body hue (yellow dotted lines). *Cephalotes simillimus*. Image by Frederic Petitclerc.

**Figure 36:**
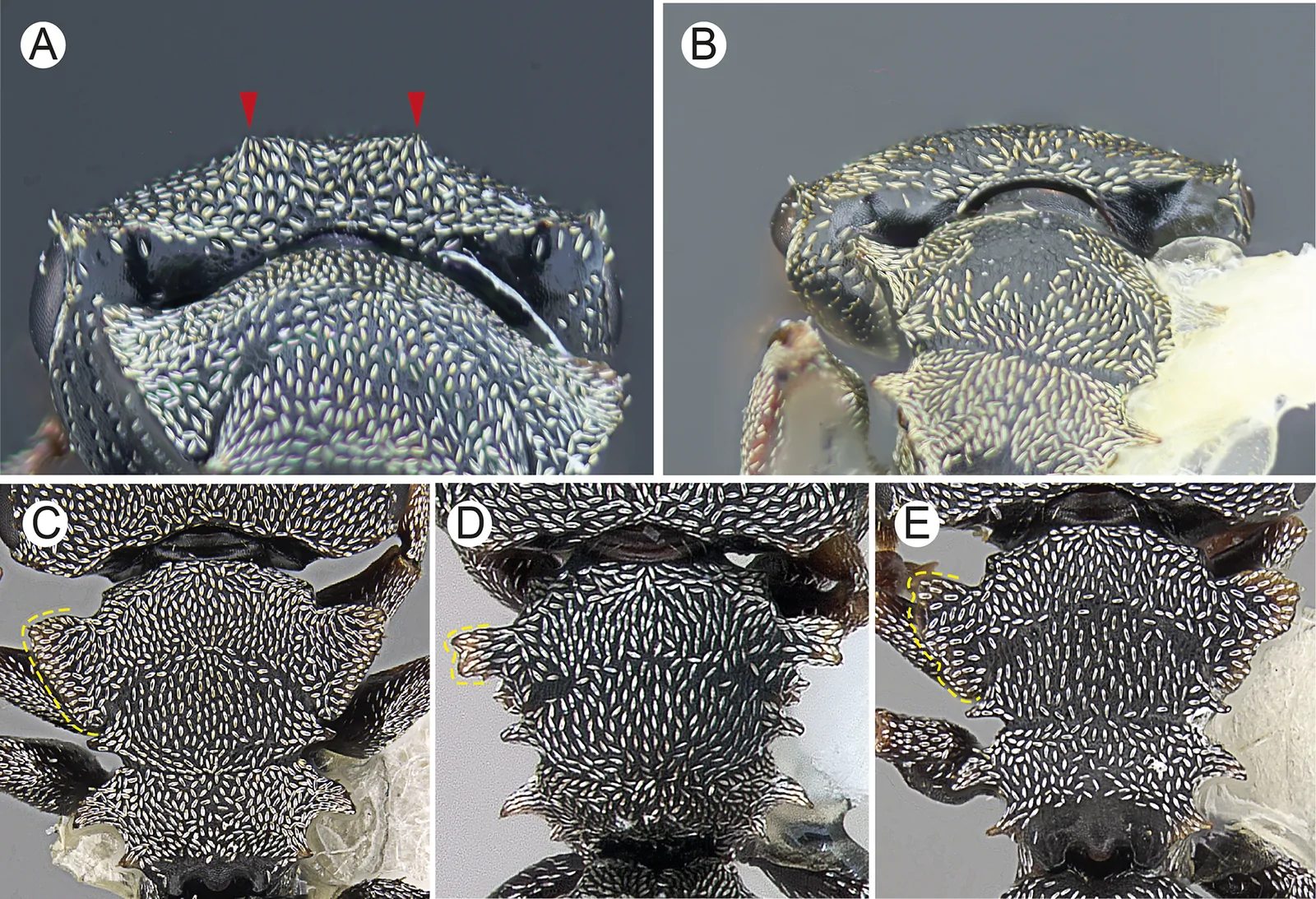
Vertex (posterior view). (A) Denticulate (red arrowheads). *Cephalotes cordatus*. (B) Plain. *Cephalotes depressus*. Images by Akayky Yumbay. Pronotal lamellar margin (dorsal view). (C) Mostly plain (yellow dotted line). *Cephalotes cordatus*. Image by Wade Lee. (D) Bidentate (yellow dotted line). *Cephalotes depressus*. Image by April Nobile. (E) Crenulate or notched (yellow dotted line). *Cephalotes pavonii*. Image by Wade Lee.

**Figure 37:**
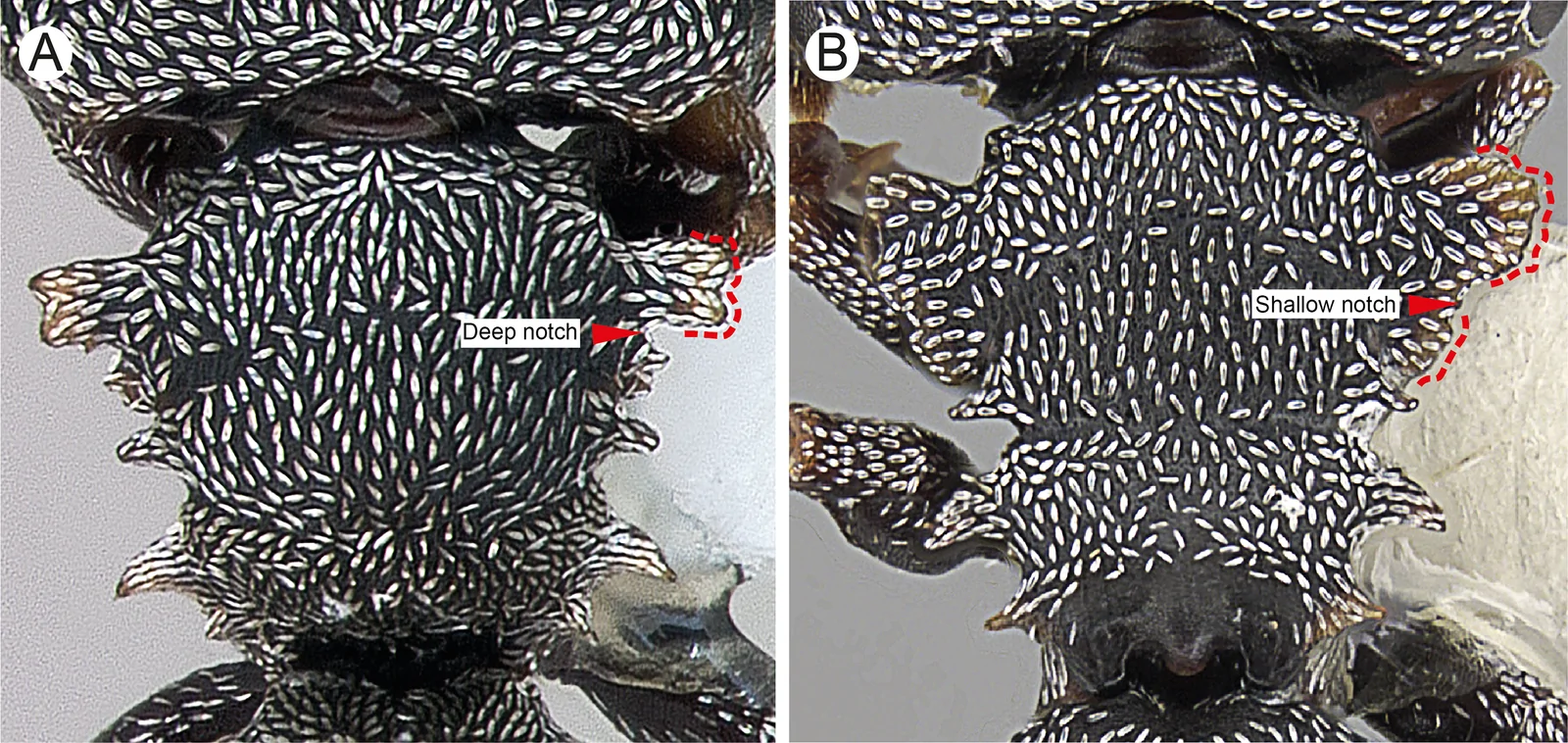
Pronotal lamella (dorsal view). (A) Bidenticulate (dotted red line), followed by deep notch (arrowhead). *Cephalotes depressus*. Image by April Nobile. (B) Broad and crenulate, with small notch medially. *Cephalotes pavonii*. Image by Wade Lee.

**Figure 38:**
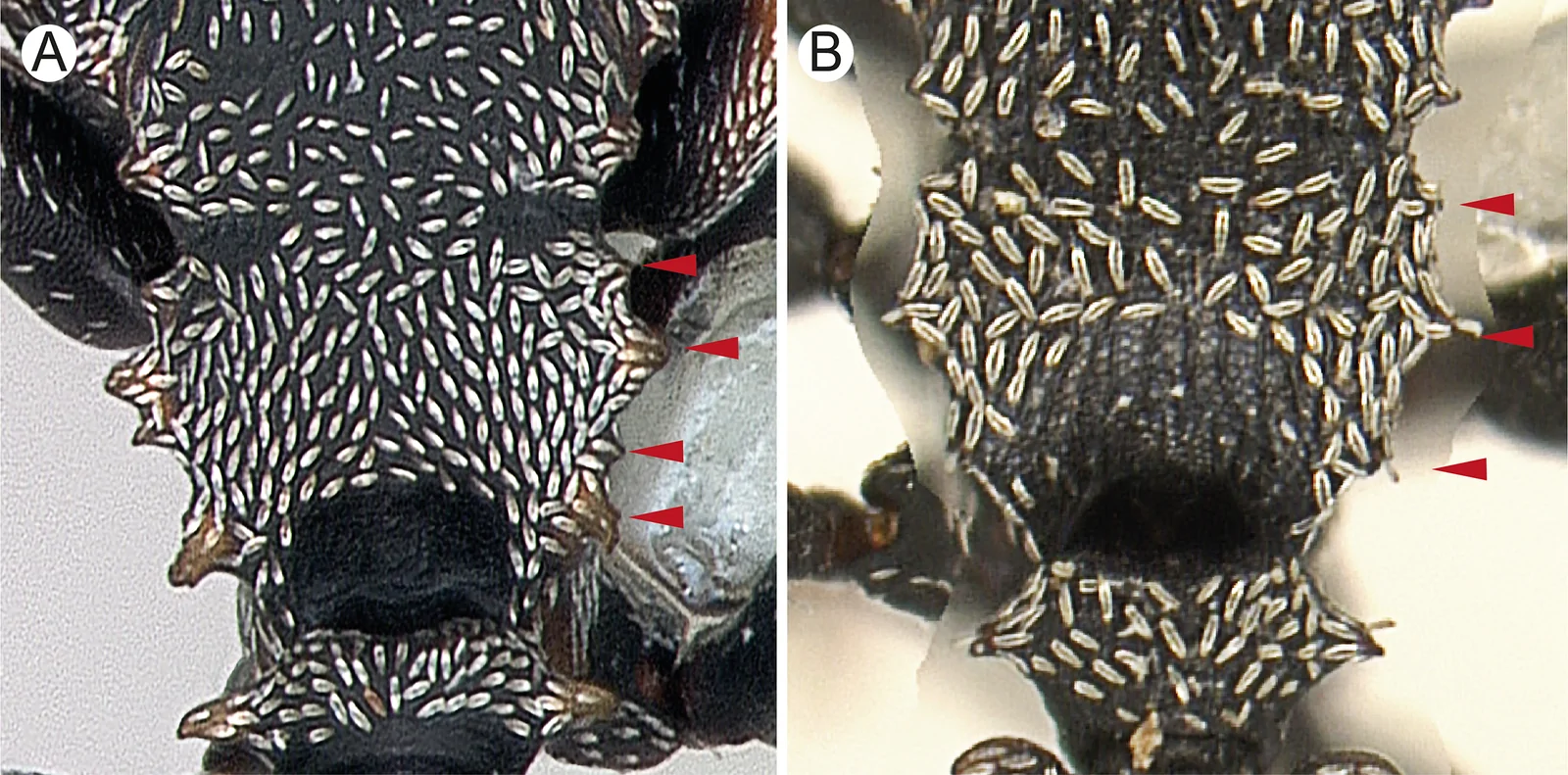
Propodeal denticles (dorsal view). (A) Four to five (red arrowheads). *Cephalotes targionii*. Image by April Nobile. (B) Three (red arrowheads). *Cephalotes peruviensis*. Image by C. Richart.

**Figure 39:**
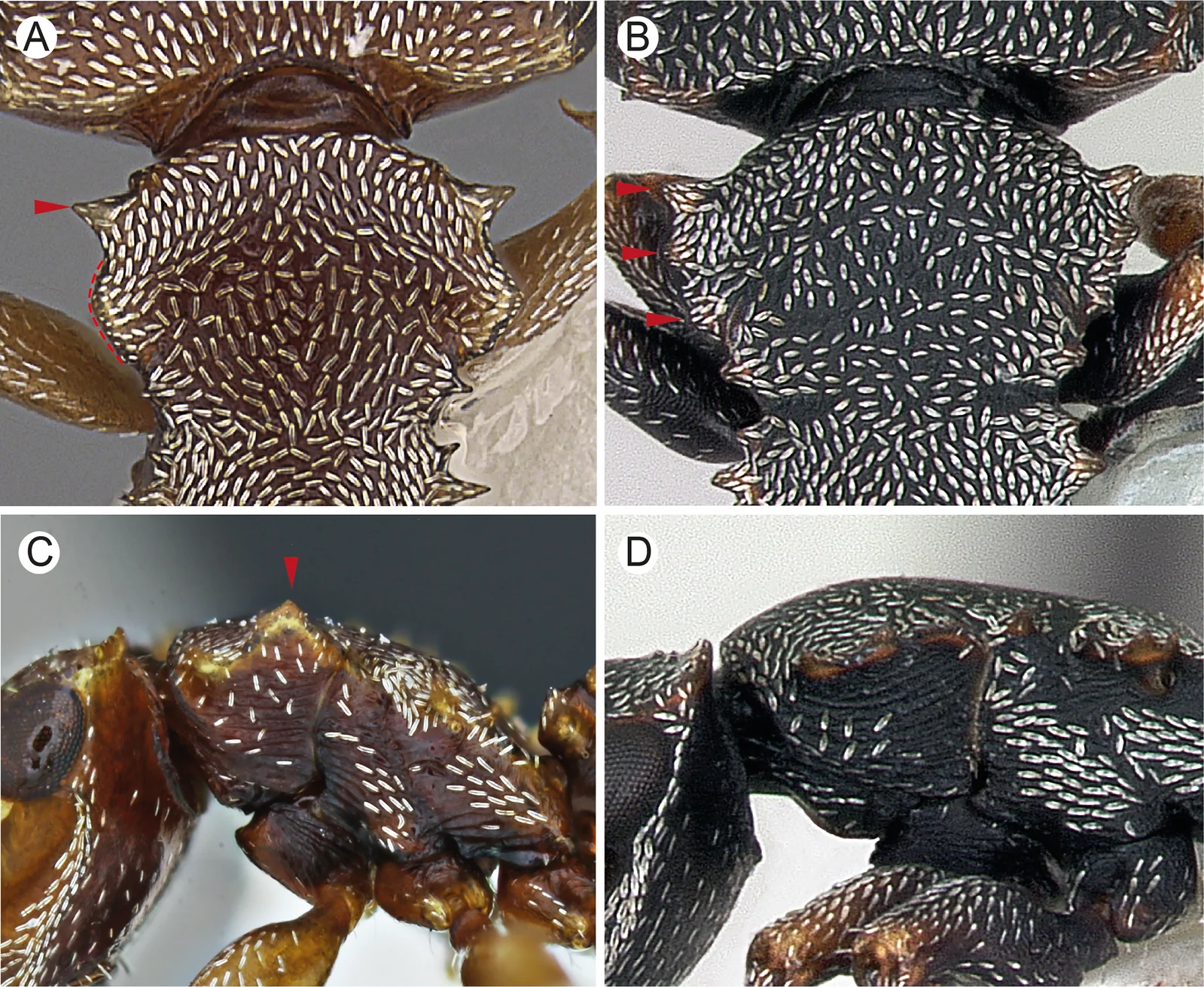
Lateral pronotal margin (dorsal view). (A) With spine anteriorly (red arrowhead) followed by broad, crenulate lobe upturned. *Cephalotes dentidorsum*. Image by Wade Lee. (B) With three membranaceous denticles (red arrowheads). *Cephalotes targionii*.Image by April Nobile; Lateral pronotal margin (lateral view). (C) Crenulate lobe upturned (red arrowhead). *Cephalotes dentidorsum*. Image by Akayky Yumbay. (D) Without upturned lobe. *Cephalotes targionii*. Image by April Nobile.

**Figure 40:**
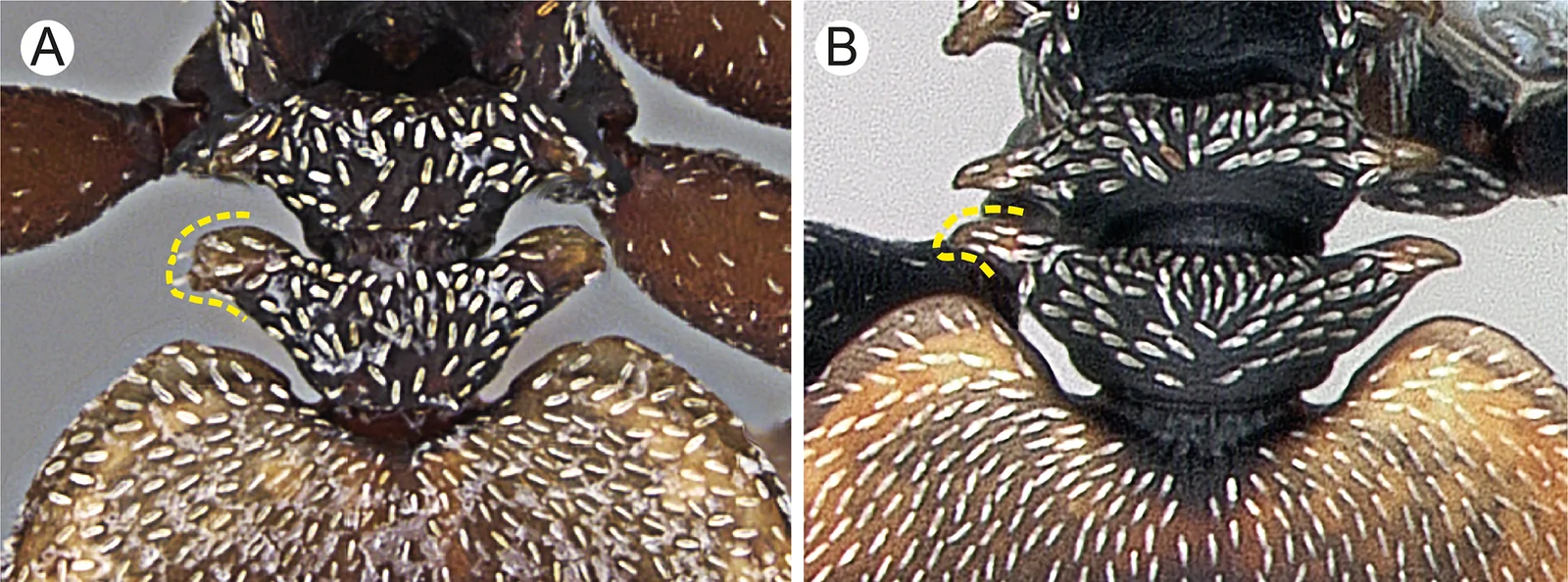
Postpetiolar lateral projection (dorsal view). (A) Lamellar, wing-shaped (yellow dotted line). *Cephalotes goeldii*. Image by Wade Lee. (B) Spiny (yellow dotted line). *Cephalotes targionii*. Image by April Nobile.

The Greek symbol ☿ is used to denote a worker or a soldier specimen.

***Cephalotes esthelae* Ilguan & Troya sp. nov.**

LSID: urn:lsid:zoobank.org:act:F29AD21C-7825-444C-A69A-DDF51C70D60E.

Figures 41A–41D (☿ holotype); Figs. 42A–42D (☿ soldier); Fig. 6C (distribution of records).

**Figure 41:**
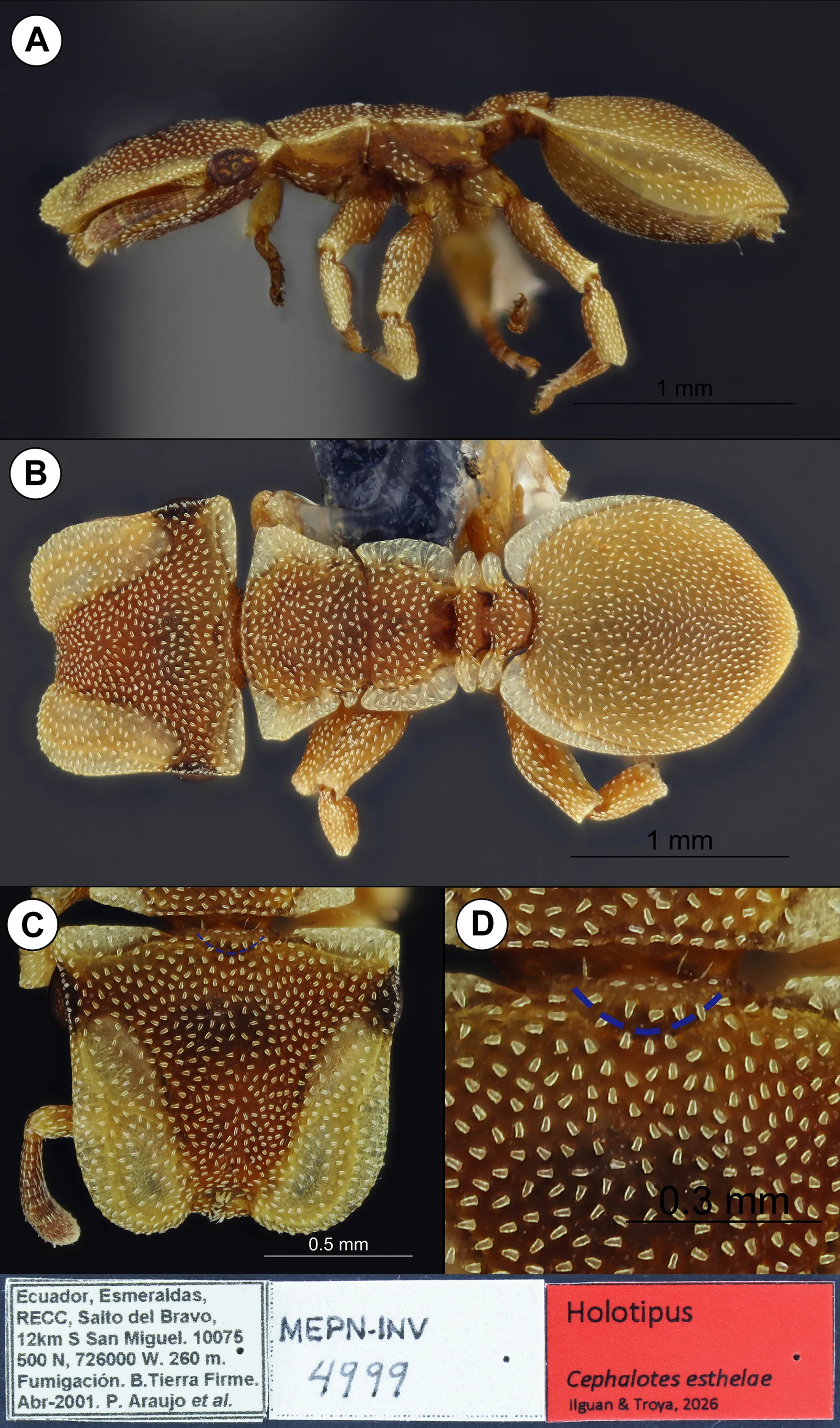
Holotype worker of *Cephalotes esthelae* sp. nov. (MEPN: MEPNINV4999). (A) Full body, lateral view. (B) Full body, dorsal view. (C) Head, full-face view. (D) Close-up of concavity on posteromedian cephalic margin (blue dashed line). Collection labels are shown at the image’s foot. Image by Elian Ilguan.

**Figure 42:**
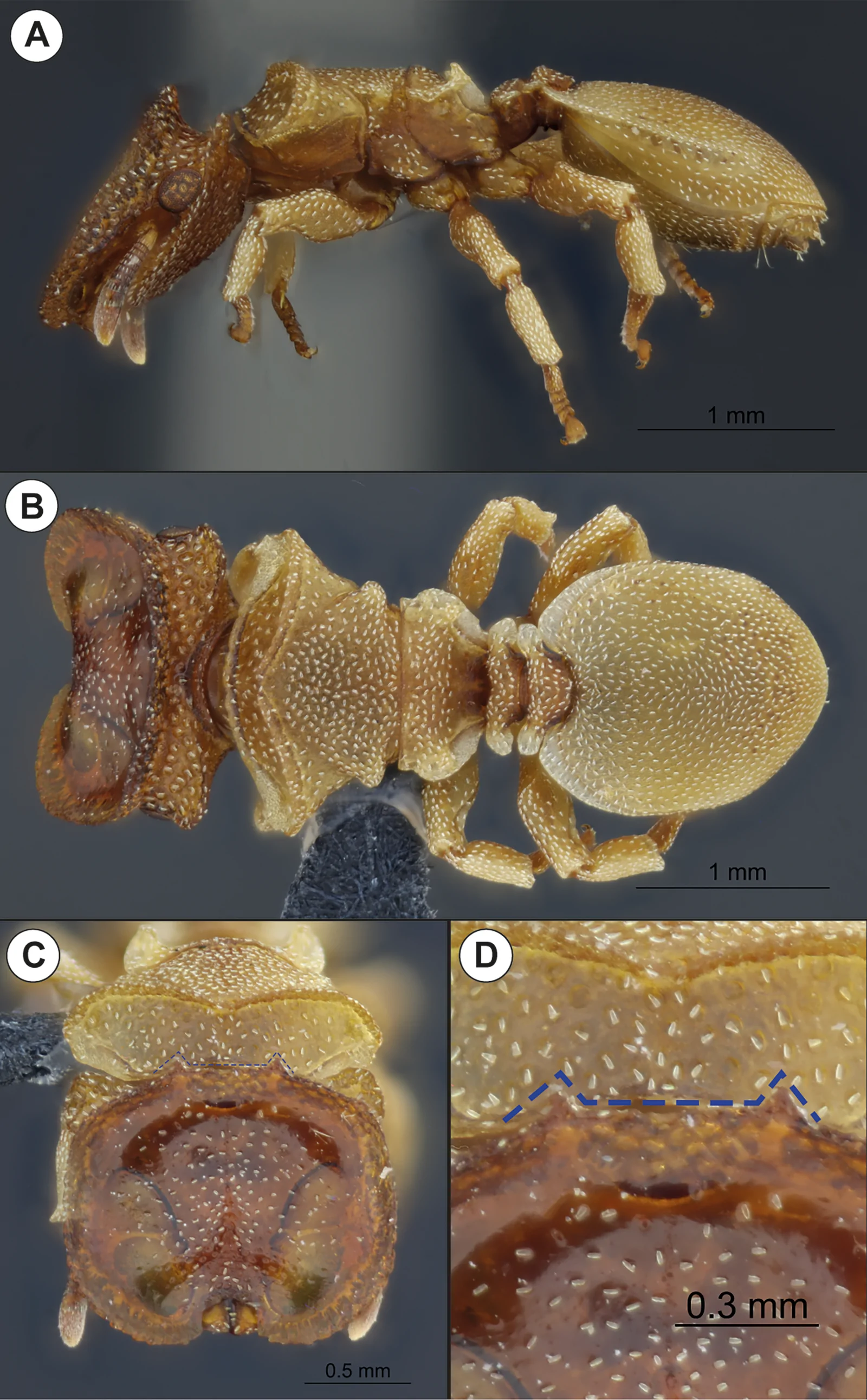
Soldier of *Cephalotes esthelae* sp. nov. (MEPN: MEPNINV4996). (A) Full body, lateral view. (B) Full, body, dorsal view. (C) Head, full-face view. (D) Close-up of teeth on posterior cephalic margin (blue dashed line). Image by Elian Ilguan.

**Type material.** Holotype. ☿; ECUADOR: Esmeraldas: Reserva Ecológica Cotacachi Cayapas (RECC), Salto de Bravo, 12 km S San Miguel, 10075500°N, 726000°W [0.682 to −78.969], 260 m, Fumigación [method], B. Tierra Firme [habitat], Abril 2001, P. Araujo et al. [F. Bersosa, P. Rosero, A. Ortega, P. Guerra, V. Granda, A. Troya] [leg.] (MEPN: MEPNINVINV4999).

**Paratypes.** 5 ☿. Same data as holotype (MEPN: ☿ soldier, MEPNINV4996; ICN: ☿ soldier, MEPNINV4997; ICN: ☿, MEPNINV5002; MCZC: ☿, MEPNINV5001); Same data as holotype, except: La Tabla, 7 km de Playa de Oro, río Santiago, 00°50′42″N, 78°44′40″W [0.845 to −78.7444], 120 m, 01-Abr-2001 (MEPN: ☿, MEPNINV28234).

**Etymology.** The specific name is dedicated to Esthela, mother of Elian Ilguan, one of the authors of this work. This is in recognition of her unconditional support and constant guidance in his personal and scientific training. The epithet is formed in the Latin feminine, singular genitive (International Code of Zoological Nomenclature: Art. 31.1.2).

**Diagnosis.** Provisionally placed in the *pinelii*. + *grandinosus* species-group, *sensu* Price et al. (2022), which is characterized by continuous mesosoma dorsally, without grooves; hind femur with ventral lamellar crest; mesosoma and petioles with lamellae; dorsoventrally flattened body. Workers: In full-face view, posteromedian cephalic margin concave (Figs. 41C and 41D); in dorsal view: posterior head margin broader than pronotum by about ¾ to one maximum eye diameter (Fig. 41B); propodeal lamellae transversely striate (Fig. 41B). Soldiers: Cephalic disc bigger (CI: 114-130 mm) than all other known soldiers in this species-group; in lateral view, propodeal dorsum slightly higher than mesonotal dorsum (Fig. 42A). Workers and soldiers: Body with abundant foveae bearing simple, mostly subtriangular-like hairs (Figs. 1A2, 41B–41D, 42B).

**Worker description.** Measurements (*n* = 5): HW: 1.19–1.5 (1.3); HL: 0.99–1.19 (1.19); EL: 0.24–0.26 (0.25); CCHA: 0.2–0.24 (0.2); PW: 0.92–1.03 (1); WL: 1.1–1.16 (1.1); PTL: 0.18–0.21 (0.18); PTW: 0.62–0.66 (0.66); PPL: 0.2–0.24 (0.22); PPW: 0.57–0.66 (0.64); GL: 1.31–1.51 (1.42); GW: 1.14–1.18 (1.18); HBL: 0.31–0.34 (0.33); HBW: 0.11–0.13 (0.13); Indices. CI: 114.71-130 (125); OI: 19.3–21.61 (19.68); PI: 26.67–32.87 (26.67); HBI: 37.14–42.86 (38.67) TL: 3.69–4.31 (4.08).

**Head.** *In frontal view:* Subquadrate, wider than long (CI: 115–130). Occipital corner with subhyaline lamella; posterolateral margin of frontal lobe covering most of eye dorsally; posteromedian margin deeply concave; width of posterior margin broader than maximum pronotal width; anterolateral margin feebly convex. Masticatory margin of mandible with one to two apical teeth, followed by three distal denticles, decreasing in size from apex to base. *In lateral view:* Anteromedian dorsal surface feebly convex, followed by slight depression and convexity on vertex (Fig. 41). Lateroventral carina present, running from anterior genal region to occipital carina (Fig. 41A). Eye ovoid. Antennomeres II–X about same length, excluding scape and two-segmented club; apical antennomere about two times longer than precedent.

**Mesosoma.** *In dorsal view:* Anterior pronotal margin convex; humerus with subhyaline, triangular lamella, truncate distally, lateral margin straight, forming approximately 60° angle with imaginary intersecting horizontal line (Fig. 41B). Promesonotal suture absent. Mesonotum with subhyaline, triangular lamella directed anterolateriad. Propodeum with broad, subhyaline lamella, shorter anteriorly, broader posteriorly; lateral margin straight to feebly convex; anterior lamellar margin round to weakly acute, posterior lamellar margin round. *In lateral view:* Propodeal spiracle round, just below propodeal lamella. Meso- and metapleural sutures absent; metanotal groove absenton. Metapleural gland opening barely discernible.

**Legs.** Meso- and metafemur angulate with or without distal, subhyaline lamella on posteroventral margin. Apices of pro-, meso-, and metatibia with weakly-developed, pointed external lamella. Probasitarsus subcylindrical; meso- and metabasitarsus flattened; probasitarsus about the same length of protarsomeres II–IV; meso- and metabasitarsus longer than respective tarsomeres II–V combined.

**Petiole and postpetiole.** *In lateral view:* Petiole about as high as long; dorsal surface of node relatively flat; subpetiolar process longitudinally ridged, with weakly developed, round tip directed ventrad. Postpetiole longer dorsally than ventrally; dorsal surface of postpetiolar node relatively flat; subpostpetiolar process well-developed, anteriorly lip-shaped. *In dorsal view:* Petiole, excluding lamellae, shorter than broad; anterior petiolar margin slightly concave; petiolar lamella well-developed, subhyaline, rhomboid-shaped, slightly longer than broad, distally subacute. Postpetiole, excluding lamellae, about as long as broad; anterior postpetiolar margin straight; postpetiolar lamella well-developed, subhyaline, longer than broad, distally round; nodal lamella slightly longer than width of node, postnodal lamella slightly shorter than length of postnode. Petiolar node longitudinally shorter than postpetiolar node.

**Gaster.** *In dorsal view:* Abdominal Segment IV (AIV) suboval, slightly longer (GL: 1.31–1.51) than broad (GW: 1.16–1.18); margin of posterior third straight; anteromedian margin u-shaped. Anterior subhyaline lamella present, extending posteriad to approximately half length of AIV; anterior lamellar margin round, internal margin continuous with anteromedian margin of AIV. Posterior margin of AIV subacute. *In lateral view*: AIV convex both ventrally and dorsally.

**Color and pilosity.** Body light brown, except whitish to pale yellowish frontal lobes and lamellae; cuticle overall opaque, including legs.

Head, mesosoma and petioles with moderately abundant, appressed, mostly subtriangular-like, canaliculate hairs. Mandibles with suberect hairs. Canaliculate hairs on AIV relatively less abundant than rest of body and mostly linear or rectangular-shaped.

**Integument and sculpture.** Integument mostly foveate-reticulate, except on AIV and legs. Striae absent from head, mesosoma and petiolar dorsum; shallow striae present on dorsal and lateroventral surface of AIV, and ventrally on head. Propodeal and gastral lamellae bearing faint, horizontal and longitudinal striae, respectively (Fig. 41B).

**Soldier description.** Measurements (*n* = 2): HW: 1.61–1.64; HL: 1.25–1.28; EL: 0.26; CCHA: 0.33; PW: 1.58–1.73; WL: 1.42–1.47; PTL: 0.22–0.24; PTW: 0.72–0.73; PPL: 0.24–0.26; PPW: 0.68–0.7; GL: 1.64–1.69; GW: 1.34–1.36; HBL: 0.28–0.33; HBW: 0.15; **Indices.** CI: 116.28-119.5; OI: 16.43; PI: 30.3–32.84; HBI: 46.67–53.85; TL: 4.77–4.94.

**Head.** *In frontal view*: Wider than long (CI: 116–119), disc-shaped, overall margin raised so that base of disc not visible laterally; short, longitudinal convexity anteromedially on disc floor; anterior margin slightly crenulate; posterior margin with pair of teeth medially; anterior margin with u-shaped cleft medially, lateral margin straight, anterior and posterior margins convex. Occipital corner bearing round, salient carina ventrally. Maximum width similar to that of anterior pronotal crest. Masticatory margin of mandible with apical tooth, basally edentate. *In lateral view:* Dorsal margin mostly flat. Lateroventral carina present, running from anterior genal region to posteroventral occipital carina. Posterior cephalic margin strongly concave. Eye: as worker. Antenna: as worker.

**Mesosoma.** *In dorsal view:* promesonotum triangular-shaped, maximum width about two times mesopropodeal suture; promesonotal suture absent; anterior pronotal margin convex; pronotal crest acute anteriorly; promesonotal suture deeply impressed; lateral pronotal margin slightly concave, forming approximately 60° angle with imaginary intersecting horizontal line (Fig. 42B). Mesonotum with salient posterolateral, round carina. Propodeum subtriangular, with broad hyaline lamella laterally, anterior lamellar margin round; anterolateral propodeal margin with tiny denticle. Propodeal declivity concave. *In lateral view:* pronotal anterior margin straight; pronotal crest raised; mesonotum straight; propodeal declivity pronounced; posterior propodeal margin continued with dorsal margin, without forming angle. Posterior propodeal carina with vertically raised, round-tipped denticle. Mesopleural suture absent; mesometapleural suture present, without groove. Propodeal spiracle round, about two spiracle diameters below propodeal carina. Metapleural gland opening barely discernible. *In frontal or dorsal views*: pronotal margin with subhyaline lamella.

**Legs.** As worker.

**Petiole and pospetiole.** As worker.

**Gaster.** As worker, except AIV longer (GL: 1.64–1.69) than broad (GW: 1.34–1.36).

**Color and pilosity.** As worker, except: head mostly ferruginous to dark brown; dorsum bearing much less appressed canaliculate hairs.

**Integument and sculpture.** As worker, except head dorsum polished.

**Comments**

The workers of *C. esthelae*, as compared to members of the *pinelii* + *grandinosus* species-group, the group to which we suspect this species might be part of, are medium-sized (WL: 1.01–1.16 mm), and considering only the head size, this species is amongst the largest (HL: 0.92–1.09 mm; HW: 1.15–1.28 mm). In contrast, the average head size of the workers of the other *pinelii* + *grandinosus* members is HL: 0.79–1.12 mm, and HW: 0.87–1.25 mm. The soldiers, however, exhibit a significantly longer head (HL: 1.60 mm) compared to that of the other known soldiers of the *pinelii* + *grandinosus* species-group (HL: 1.12–1.37 mm). From a morphological perspective *C. persimilis* and *C. persimplex* are the closest species to *C. esthelae*, which differs from them in the following: in frontal view, the head is broader than the pronotum by about one maximum eye length (Fig. 41B), while in both *C. persimilis* and *C. persimplex* the head is almost as broad as the pronotum (CASENT0173700, CASENT0922544); the posterior cephalic margin has a median concavity (Figs. 41C and 41D), which is absent in *C. persimilis* and *C. persimplex*. The worker of *C. foliaceus* is the only known caste in the *pinelii* + *grandinosus* group with a similarly big head (HL: 1.10 mm; HW: 1.43 mm), but this is easily distinguished from the worker of *C. esthelae* by the lamella of the first gastral tergite, which in *C. esthelae* runs posteriad to about the median gastral region (Fig. 41B), whereas in the worker of *C. foliaceus* the lamella surrounds the complete gastral margin (CASENT0904925). *Cephalotes esthelae* and *C. persimplex* are the only species in the genus showing subtriangular-shaped canaliculate hairs on the dorsum of head and mesosoma. These are usually discernible with high magnification (≥60×).

A single nontype worker (Fig. 43), collected near Lumbaqui town (Sucumbíos province) in a well-preserved Amazonian cloud forest, to the eastern Andean chain, shows a longer gaster (GL: 1.51 mm) as compared to the workers of the type material (GL: 1.31–1.42 mm) which were collected in lowland rain forests in Esmeraldas province, to the western side of the Andes. In addition, the gastric hairs of this Amazonian specimen are smaller and less abundant (Fig. 43) as compared to those of the specimens of the type series.

**Figure 43:**
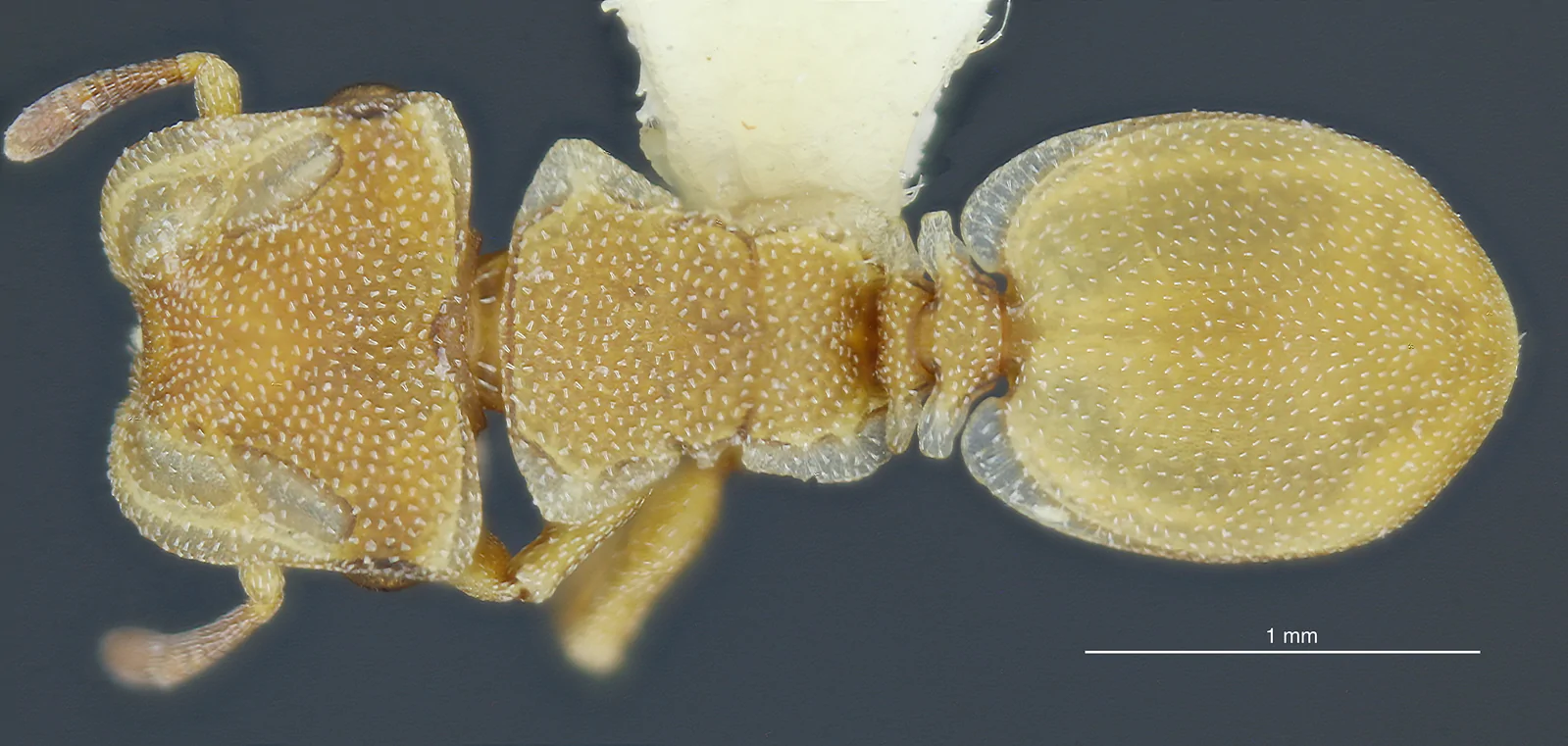
Worker of *Cephalotes esthelae* sp. nov. (MEPN: MEPNINV18178) from Lumbaqui town, Amazonia. Note the difference in gaster length and in the gastric hair size, as compared to the holotype. Image by Adrian Troya.

**Distribution notes**

*Cephalotes esthelae* has so far been found exclusively in Ecuador, inhabiting well-preserved terra firme forests with an elevation range between 120 m and 550 m. In the northern part of this country, its distribution reaches pristine forests near a small river called Puchuchoa, close to the town of Lumbaqui, in the western side of the province Sucumbíos. This is a transition zone between the lowland rain forests of Amazonia and the Andean foothills, mostly dominated by pre-mountainous cloud forests. To the west, this species is found in lowland well-preserved forests of the Reserva Ecológica Cotacachi Cayapas, province of Esmeraldas, which is part of the southern section of the Chocó Biogeographic. These ecosystems, characterized by their high biodiversity and endemism, are currently under increasing anthropogenic pressure due to high deforestation rates and changes in land use (Kleemann et al., 2022; Rivas, Guerrero-Casado & Navarro-Cerrillo, 2024), with still unknown, but likely negative impacts on the populations of many species.

*Other material examined*

☿, ECUADOR, Sucumbíos, Lumbaqui, Quebrada Puchuchoa, 00°04′51,4″N, 77°16′43,6″W [0.081 to −77.2788], 550 m, 23-Ene-2003 (MEPN: ☿, MEPNINV18178).

**Distribution.** Ecuador: Esmeraldas, Sucumbíos.

***Cephalotes sacha* Yumbay & Troya sp. nov.**

LSID: urn:lsid:zoobank.org:act:894642B2-D783-4E44-ABA0-EC3C8809197B

Figures 44A–44F (☿ holotype); Figs. 45A–45C (☿ soldier); Fig. 5B (distribution of records).

**Figure 44:**
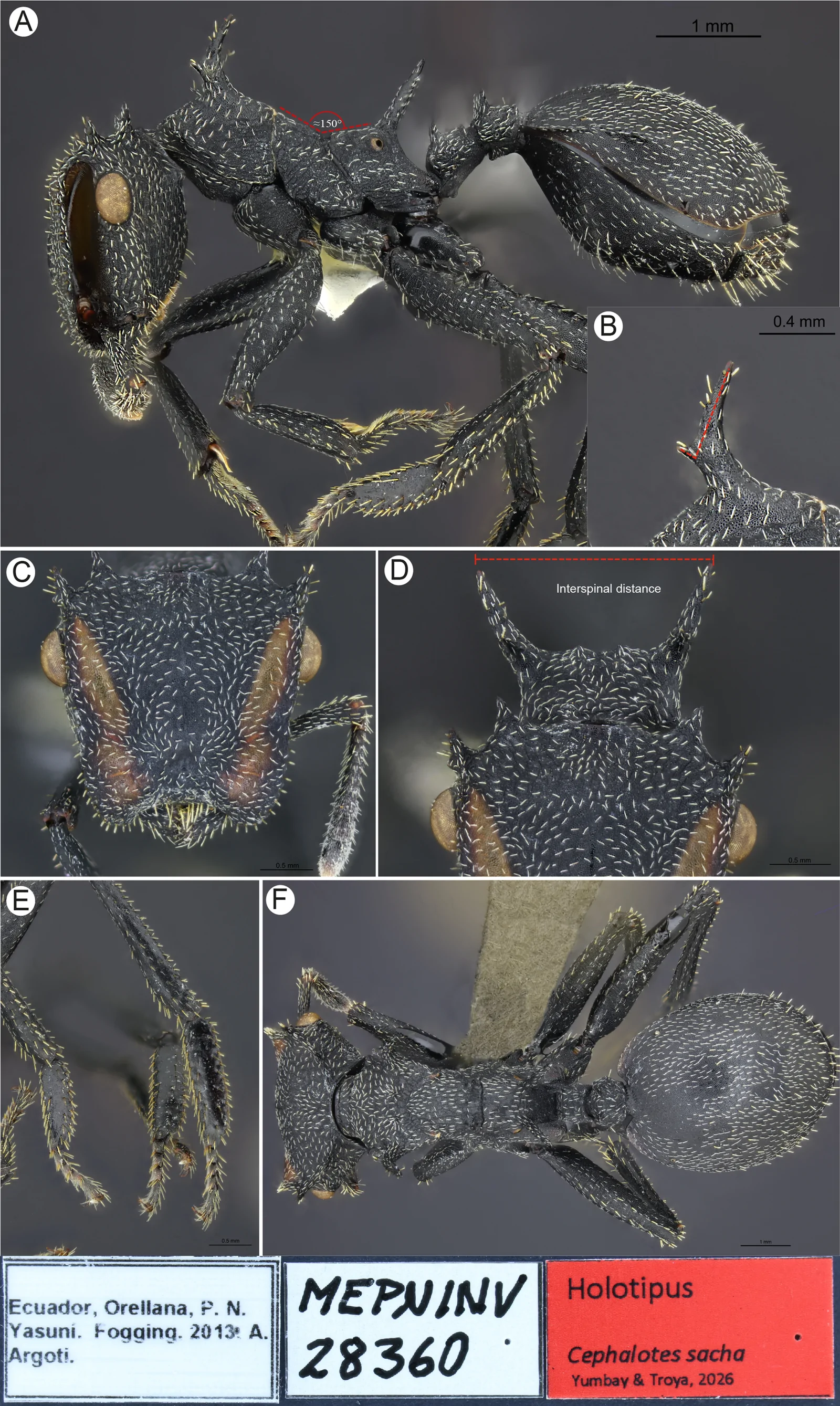
Holotype worker of *Cephalotes sacha* sp. nov. (MEPN: MEPNINV28360). (A) Full body, lateral view. (B) Pronotal spine, lateral view. (C) Head, full-face view. (D) Close-up of pronotal spines, frontal view. (E) Meso- and metatarsi, lateral view. (F) Full body, dorsal view. Collection labels are shown at the image’s foot. Image by Akayky Yumbay.

**Figure 45:**
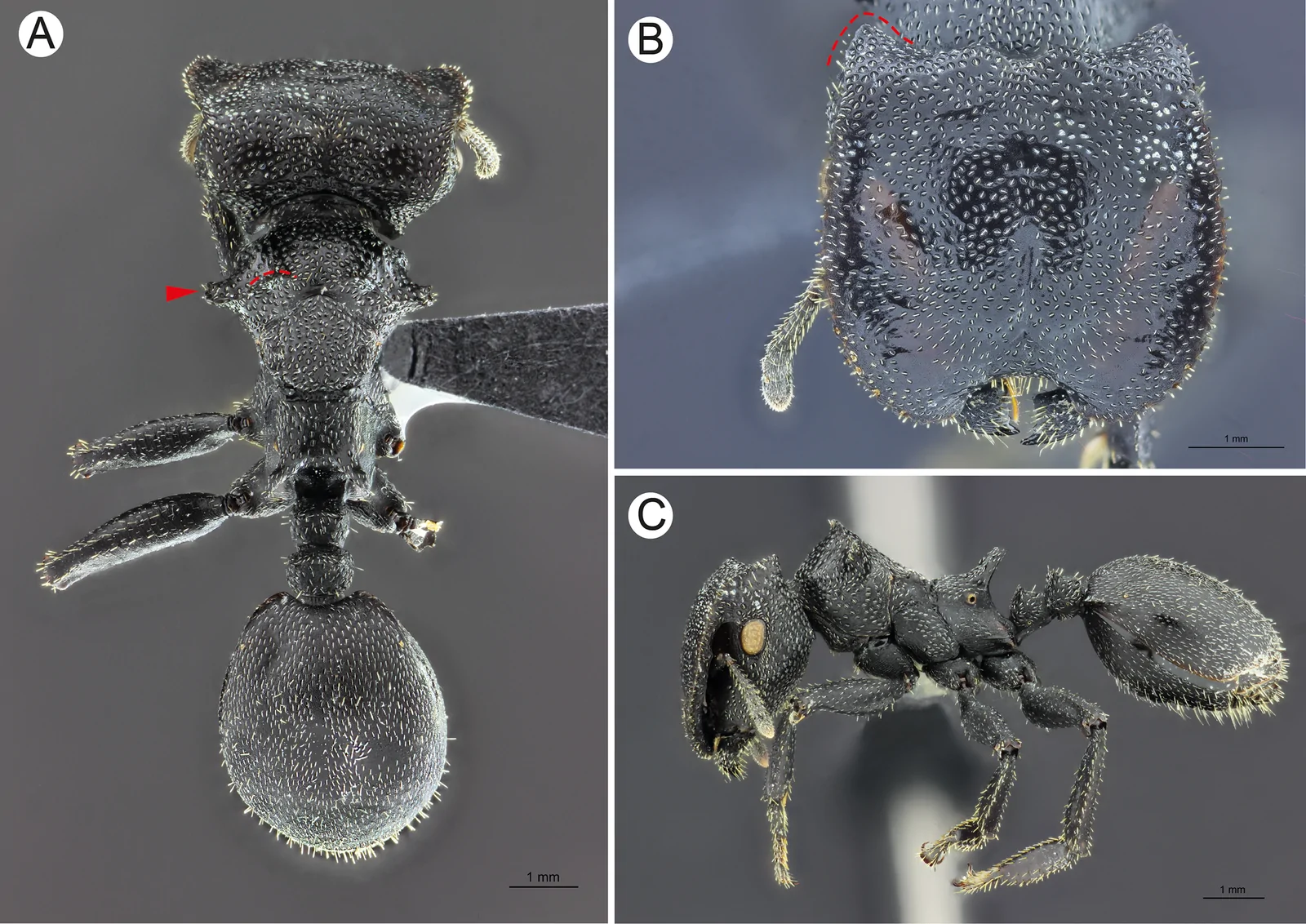
Soldier of *Cephalotes sacha* sp. nov. (MEPN: MEPNINV28356). (A) Full body, dorsal view. (B) Head, full-face view. (C) Full body, lateral view. Red arrowhead: angulate projection with truncate tip; Red dashed line: pronotal blunt tooth. Image by Akayky Yumbay.

**Type material.** Holotype. ☿; ECUADOR: Orellana: P. N. [Parque Nacional] Yasuní. Fogging [method]. 2013. A. Argoti [leg.]. (MEPN: MEPNINV28360).

**Paratypes**: 12 ☿. Same data as holotype (MEPN: ☿ soldier, MEPNINV28356; ICN: ☿ soldier MEPNINV28448; DZUP: ☿, MEPNINV28361; IAVH: ☿, MEPNINV28363; ICN: ☿, MEPNINV28364; QCAZ: ☿, MEPNINV28358; MCZC: ☿, MEPNINV28359; USNM: ☿, MEPNINV28362); same data as holotype, except: Aguarico Cononaco, Guacamayo Saladero, 01°17′40″S 76°03′39″W [−1.2944 to −76.0608], 215 m Fogging, 02-feb-14 A. Troya & P. Duque (MEPN: ☿ soldier, MEPNINV35175; ☿, MEPNINV 35174); ECUADOR: Sucumbíos: Lumbaqui, Fogging, ene-2003, P. Araujo (MEPN: ☿, MEPNINV37758; MCZC: ☿, MEPNINV37759).

**Etymology.** The specific epithet is derived from the Kichwa “sacha”, meaning “forest” or “jungle”. The name alludes to the Amazonian rainforests where specimens of this species were found. This is a tribute to both the forests and the indigenous language spoken by numerous peoples and communities in Ecuador. As such, we highlight the need to preserve our natural and cultural heritage. The epithet is a noun in apposition, thus indeclinable (International Code of Zoological Nomenclature: Arts.11.9.1.2.; 31.2.1).

**Diagnosis.** A member of the *atratus* species-group which is characterized mainly by the following: head corner with pair of spines, and humerus with one spine which is usually feebly bifurcated at tip. Workers: Mesonotum and propodeum forming a ~150° angle, in lateral view (Fig. 44A); integument opaque; in lateral view, postpetiolar dorsal spines longer than subpostpetiolar process, in lateral view; body with simple hairs without foveae (Figs. 44A, 44C, 44D); in dorsal view: promesonotum without Y-shaped carina (Fig. 44F); anterior projection of pronotal spine about four times shorter than posterior projection, forming straight angle with it (Fig. 44B). Soldiers: In dorsal view, pronotum with pair of blunt teeth dorsally, and pair of salient, horizontally directed, angulate projections with truncate tips laterally (Fig. 45A); in full-face view, cephalic disc subquadrate with acute projection on occipital corner (Fig. 45B); in dorsal view, anterolateral margin of gaster bearing slightly salient, non-translucent lamella which fades away in the first anterior fourth dorsally (Fig. 45C).

**Worker description.** Measurements (*n* = 10): HW: 2.13–2.62 (2.34); HL: 2.1–2.53 (2.34); EL: 0.4–0.5 (0.43); PW: 1.5–1.79 (1.63); WL: 2.2–2.62 (2.49); PTL: 0.44–0.59 (0.53); PTW: 0.32–0.55 (0.54); PPL: 0.44–0.55 (0.48); PPW: 0.58–0.67 (0.64); GL: 2.3–2.88 (2.56); GW: 2.1–2.27 (2.17); HBL: 1.28–1.66 (1.44); HBW: 0.29–0.42 (0.35); ID: 1.43–1.82 (1.78); **Indices.** CI: 88.46-103.53 (100); OI: 18.19–21.51 (18.19); PI: 93.75–137.5 (97.6); HBI: 22.5–27.8 (24.44); TL: 7.51–9.21 (8.37).

**Head.** *In frontal view:* Subquadrate, slightly longer than wide (CI: 88–103); occipital corner with pair of equally sized spines, each about ¾ maximum eye length; posterior margin feebly convex medially; lateral margin of frontal lobe mostly straight, weakly crenulate, slightly concave medially; anterior margin of frontal lobe weakly crenulate, round. Eye completely visible. Anterior clypeal margin broadly concave, at least ½ of mandible dorsum visible. External mandibular margin convex, masticatory margin with two apical teeth, followed by one to three denticles distally. *In lateral view:* Slightly convex medially; lateroventral carina present, running from gena to posteroventral ocular margin. Postgena convex. Nuchal carina well-developed, slightly pointed anteriorly. Eye ovoid. Antennomeres II–VIII about same length, each smaller than apical antennomeres forming a three-segmented club; apical antennomere about ¼ longer than precedent.

**Mesosoma.** *In dorsal view:* Anterior pronotal margin slightly convex, humerus with bifurcate spine, feebly inclined laterad. Anteroventral pronotal carina salient, directed anterolaterally. Maximum pronotal width about 1.5 times broader than width of mesonotum. Promesonotum trapezoid, with angular lateral margins. Promesonotal suture absent. Mesopropodeal groove strongly impressed. *In lateral view*: Pronotum convex. Propleuron convex, slightly angulate. Mesopleural suture absent. Mesometapleural groove impressed, vanishing before reaching ventral cleft. Propodeum with long dorsolateral spine, about six times as long as basal width, inclined posteriad, making about 150° angle with propodeal dorsal margin; propodeal declivity straight, inclined anterad, making about 90° angle with dorsum of straight propodeum. Propodeal spiracle circular, located about 1.5–2 spiracular diameters from propodeal dorsal margin. Orifice of metapleural gland opening slit-shaped, horizontally oriented, barely discernible, bounded distally by horizontal carina.

**Legs.** Pro-, meso-, and metafemur broadened basally, distally narrowed; protibia angulate, slightly flattened laterally; meso- and metatibia angulate, flattened laterally; meso- and metabasitarsus strongly flattened laterally, longer than respective tarsomeres II–V combined. Tarsal claws bearing small preapical tooth.

**Petiole and postpetiole.** *In lateral view:* Petiolar node, excluding pair of dorsal spines, slightly longer than high; nodal spine well-developed, slightly longer than basal width; dorsal nodal surface convex; subpetiolar process well developed, longitudinally ridged, with blunt, anteroventral tip. Postpetiolar node about as long as high; postnodal spine well-developed, slightly longer than nodal spine; dorsal postnodal surface slightly convex; subpostpetiolar process weakly developed, lip-shaped. *In dorsal view*: Petiole as long as broad, anterior margin feebly convex, posterior margin feebly concave; lateral margins feebly convex. Postpetiole slightly broader (PPW: 0.57–0.67) than long (PPW: 0.44–0.55), anterior margin straight, posterior margin feebly convex; lateral margins convex.

**Gaster.** *In dorsal view:* AIV oval, longer (GL: 2.30–2.88) than broad (GW: 2.01–2.27). Anterior lamella small but well-developed, extending posterolaterad to about one sixth length of AIV where it fades away; anterior lamellar margin rounded, internal margin continuous with anteromedian margin of AIV; posterior margin of AIV rounded. *In lateral view*: AIV dorsally convex; ventrally strongly convex.

**Color and pilosity.** Body opaque black, head dorsum bearing brownish to pale yellowish longitudinal stripe running from anterior margin of frontal lobe to posterolateral head margin. Dorsal and ventral surface of head, mesosoma, petioles and anterior dorsal region of AIV with moderately abundant, white to golden, mostly appressed hairs; antennae, mandibles, tibiae, tarsi and gaster bearing few erect and suberect hairs.

**Integument and sculpture.** Integument rugulose, composed of puncta and irregular, linear, non-continued striae located posterodorsally, laterally, and ventrally on head, dorsally on mandibles, most of mesosoma, except ventrally, legs, dorsally on petioles, and anterodorsally on AIV.

**Soldier description.** Measurements (*n* = 3): HW: 3.4–4.1; HL: 2.88–3.7; EL: 0.55–0.63; PW: 2.27–3.1; WL: 3.4–4; PTL: 0.64–0.77; PTW: 0.58–0.83; PPL: 0.58–0.67; PPW: 0.74–0.93; GL: 3.35–3.8; GW: 2.59–3.7; HBL: 1.6–1.64; HBW: 0.45–0.54; ID: 2.3–3; Indices. CI: 90.24-97.33; OI: 16.22–19.1; PI: 84.62–111.11; HBI: 28–34; TL: 10.65–12.99.

**Head**. *In frontal view*: Subquadrate, about as long as broad (CI: 90–97); anterolateral margin convex, crenulate; anterior margin strongly concave, smooth; posterior margin straight, medially bearing pair of blunt teeth; occipital corner angulate, pointed posteriad. Frontal lobe completely covering malar area and gena, and almost completely covering eye. External mandibular margin convex, masticatory margin with one apical tooth, followed by one to three tiny denticles distally. *In lateral view:* Slightly convex dorsally and ventrally; lateroventral carina absent; nuchal carina reduced. *In frontal or dorsal view:* Pronotum shorter than maximum head width. Eye: as worker. Antenna: as worker.

**Mesosoma.** *In dorsal view:* Promesonotum triangular-shaped; anterior propodeal margin convex, humerus bearing stout, salient, truncate lobe; promesonotal suture shallowly impressed. Mesonotum pentagonal; mesopropodeal suture deeply impressed. Propodeum subquadrate, bearing stout, irregular, laterally diverging spine. *In lateral view:* Pronotal top acute, straight anterior margin making 90° angle with straight promesonotal margin. Promesonotum making about 120° angle with propodeal dorsum. Propodeal spine inclined posteriad, about three times as long as basal width; posterior propodeal margin inclined anterad. Propodeal spiracle round, about two spiracle diameters from propodeal dorsum. Orifice of metapleural gland opening slit-shaped, horizontally oriented, barely discernible.

**Legs.** As worker.

**Petiole and postpetiole.** As worker, except: node about as long as high, in lateral view; nodal spines shorter, blunt-tipped; anterior tip of subpetiolar process spiny-shaped, in lateral view.

**Gaster.** As worker.

**Color and pilosity.** As worker, except: dorsal head stripes pale ferruginous, barely discernible.

**Integument and sculpture.** As worker, except: puncta smaller, and mostly on mesopleuron, propodeum, petiolar and postpetiolar dorsum, legs, and gaster. Impressed foveae on head, mesosoma, petiole and postpetiole.

**Comments**

Within the *atratus* species-group, *C. sacha* is relatively small (WL: 2.20–2.62), and except for *C. serraticeps* and *C. alfaroi*, its morphologically closest taxa, *C. sacha* differs significantly from the other group members: *C. atratus*, *C. placidus*, *C. oculatus*, and *C. opacus*. In regards to *C. serraticeps, C. sacha* differs in the following characters: in dorsal view, the promesonotum of the worker lacks a Y-shaped carina (present in *C. serraticeps*) (Fig. 16A); the anterior projection of the pronotal spine is almost six times shorter than the spine’s length (in *C. serraticeps* it is about three times shorter than the spine’s length); in lateral view, the mesonotum and propodeum make an obtuse angle of about 150° (Fig. 44A) (in *C. serraticeps* is about 180° or more, sometimes no angle is noticed) (see Fig. 11C in Pazmiño-Palomino & Troya, 2022); in frontal view, the interspinal distance (Fig. 44D) is narrower than the corresponding in *C. serraticeps*. In the soldiers, the occipital corner has a single acute carina (in *C. serraticeps* it has three distinct pointed teeth); the meso- and metapleural plates are foveate (pleural foveae are absent in *C. serraticeps*).

As of *Cephalotes alfaroi* we examined the image of the holotype worker (CASENT0904899) on AntWeb, and distinguished the following differences with respect to *C. sacha*: well-marked pleural striae (in *C. sacha* the pleural striae are shallower, less marked than in *C. alfaroi*); dorsum of gaster smooth and glossy, and in general, body relatively shiny (in *C. sacha* the gastral dorsum is opaque and striate, as well as the rest of the body); the propodeal spiracle is about three spiracular diameters off the propodeal dorsum (about 1.5–2 spiracular diameters in *C. sacha*). Regarding a non-type *C. alfaroi* worker from Corcovado National Park, at the pacific southwestern coastal region of Costa Rica (CASENT0040145), identified by Brian Fisher on AntWeb, we noticed the gastral dorsum has slight longitudinal striae anteriorly, but the rest of the integument is smooth and glossy, as in the holotype, which is from Jimenez, Limón province, in the northeastern Atlantic side of that country.

As to the soldier of *C. alfaroi*, its posterior vertexal border (near the occipital foramen) has a pair of well-developed teeth (absent in *C. sacha*); the propodeal spines of *C. alfaroi* are longer than those of *C. sacha*; the petiole of *C. alfaroi* is devoid of spines or teeth dorsally (in *C. sacha* the petiole has two well-developed, but short spines).

**Distribution notes.** *Cephalotes sacha* is so far, only known from Ecuador. It inhabits well-preserved Amazonian rainforests, from approximately 190 m up to 600 m of elevation. To the northern range its distribution reaches the forests of the Aguarico river, near Lumbaqui, a well-preserved area in western Sucumbíos province, close to the southern Colombian border. These forests are located in a transition zone between the Amazonian lowlands and the Andean foothills, which are currently subject to strong anthropic pressure (Kleemann et al., 2022). To the south its distribution reaches the southern region of Parque Nacional Yasuní, near the Cononaco river, just at the border of Orellana and Pastaza provinces, and close to the western Peruvian border. This area is mostly composed by pristine, lowland rainforests showing a high annual rainfall (Bass et al., 2010). This latter region of Parque Nacional Yasuní is arguably the most well-preserved site of the whole Ecuadorian Amazonia.

**Natural history notes**

The two specimens from Cononaco river were collected from a Myrtaceae tree, close to a stream. In winter, this collection site is probably flooded as judged by the water marks on the base of the Myrtaceae tree trunk.

**Distribution.** Ecuador: Orellana, Sucumbíos.

**New caste description**

***Cephalotes dentidorsum*** De Andrade, 1999

**Soldier** (Fig. 46)

**Figure 46:**
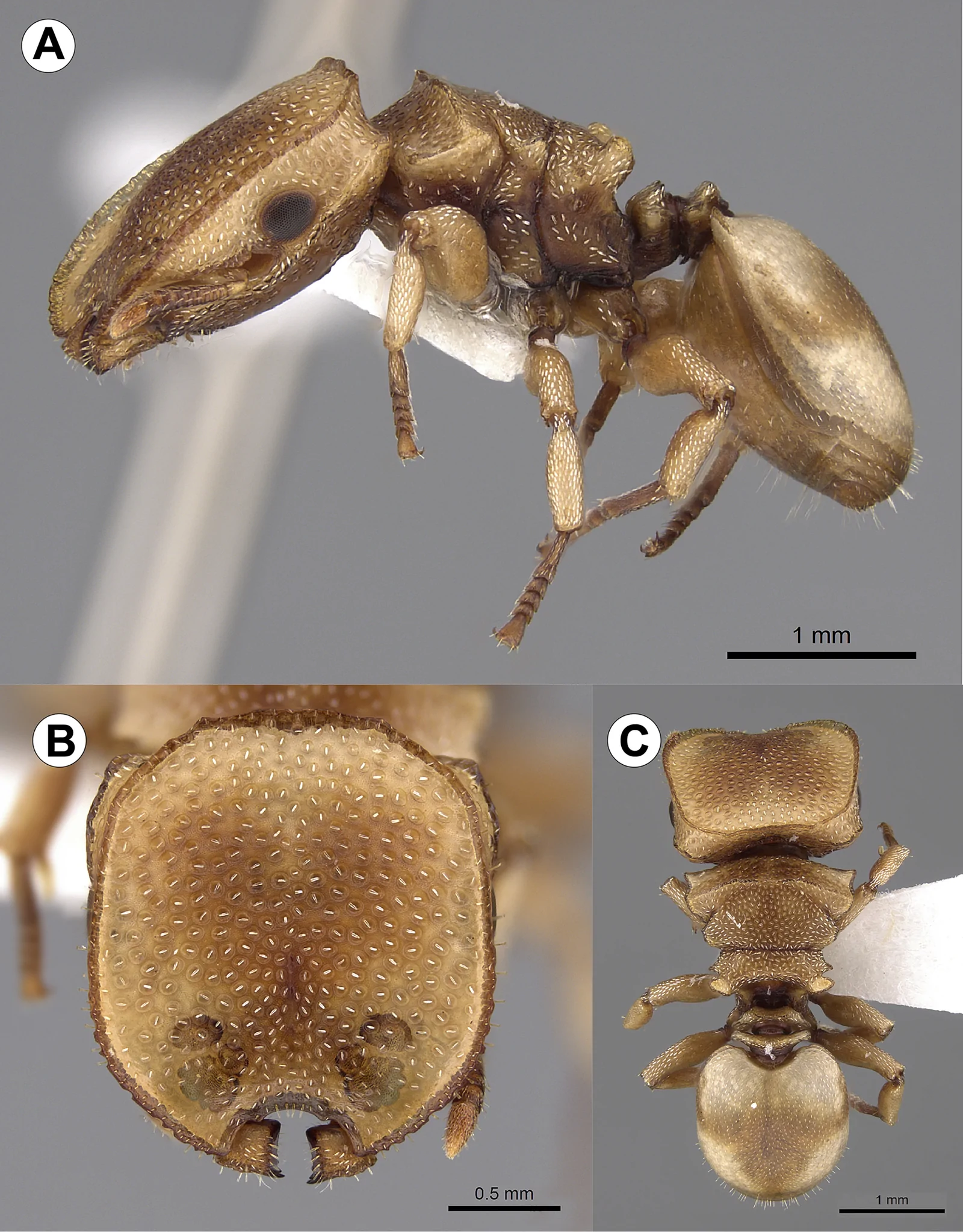
Soldier of *Cephalotes dentidorsum* (PSWC: CASENT0922531). (A) Full body, lateral view. (B) Head, full-face view. (C) Full body, dorsal view. Image by Wade Lee.

**Material examined.** Ecuador. Orellana. Parque Nacional Yasuní, −0.66667, −76.3833, 120 m, 1☿ (soldier), 2005-07-05, Argoti, A., fogging, (MEPN: MEPNINV28455); Peru. San Martín. Convento, 26 km NNE Tarapoto, −6.266667, −76.3, 220 m, 1☿ (soldier), 1986-08-20, Ward, P. S., manual, edge of second-growth rainforest, ex *Tachigalia* Benth. & Hook.f. (PSWC: CASENT0922531).

**Head**. *In full-face view*: subquadrate slightly wider than long (CI 102-102.2). Mandible with four teeth, strongly angled laterally. Clypeus with pair of denticles weakly defined. Dorsum of head disc-shaped, slightly convex anteriorly. Frontal carina crenulate anteriorly. Antenna with three-segmented club. Dorsal margin of antennal scrobe with lateral carina and posterior rounded projection. Lateroventral head margin without carina. Posteroventral cephalic corner acute, separated from dorsal cephalic disc.

**Mesosoma**. *In lateral view:* pronotum ascending, with a transversal carina raised in a crest; pronotal crest not crenulate, with a median depression. In dorsal view, anterior margin of pronotum weakly convex, lateral margins with two pairs of denticles, the anterior one acute, the second one rounded at level of transversal carina of pronotum. Mesonotum and propodeum discontinuous and flat; mesonotum with a pair of blunt rounded denticles; propodeal groove strongly impressed; dorsal and declivous faces of propodeum meeting in a distinct propodeal angle. In dorsal view, lateral margins of propodeum with a pair of median spines and obtuse denticles strongly bent dorsally, with the apices curved anteriad. Femora not angulated dorsally, mid, and hind basitarsi not flattened, with subparallel dorsal and ventral faces.

**Legs.** As worker.

**Petiole and postpetiole.** As worker, except: In dorsal view, petiole compressed anteroposteriorly, anterior margin with a distinct median concavity, lateral spines curved posteriad, dorsum with a pair of denticles, subpetiolar process broader and rounded anteriorly. Postpetiole slightly wider and longer than petiole, spines narrow and curved forward, dorsum with a “V” sharped elevation, subpostpetiolar process pronounced ventrally and compressed anteroposteriorly.

**Gaster.** As worker. Elongate, with narrow anterior lamellar expansions.

**Color and pilosity.** As worker, except: Head, dorsum of mesosoma and legs yellowish to ferruginous. Propleura predominantly ferruginous with the dorsal surface yellowish; meso- and metapleura predominantly yellowish with ferruginous spots. AIV tergite yellowish with a transverse dark macula. AIV sternite yellow-brownish. Head, mandibles, mesosoma, petiole, postpetiole and legs with appressed canaliculate hairs; frontal carinae with erect simple hairs. Gaster with appressed simple hairs; posterior portion of first sternite and edges of each tergite and sternite of gaster with erect simple hairs.

**Integument and sculpture.** As worker, except: Mandibles with reticulate sculpture; dorsum of head irregularly foveate. Dorsum of mesosoma foveate; lateral of mesosoma finely rugose with sparse foveae. Petiole and postpetiole with longitudinal striae. Gaster microalveolate.

Measurements (*n* = 2): HW: 1.82–1.98; HL: 1.78–1.94; EL: 0.24–0.33; PW: 1.63–1.76; WL: 1.32–1.62; PTL: 0.2–0.22; PTW: 0.75–0.76; PPL: 0.3–0.32; PPW: 0.76–0.79; GL: 1.3–1.41; GW: 1.45; HBL: 0.90; HBW: 0.35; Indices. CI: 102-102.2; OI: 18.1–12.1; PI: 26.3–29.3; HBI: 125; TL: 4.9–5.51.

**Comments**

Our first line of evidence that led us to determine the examined soldiers are *C. dentidorsum* is based on the comparison of a *C. dentidorsum* worker (MEPN28208) from the eastern Ecuadorian Amazon, previously reported by Pazmiño-Palomino & Troya (2022), which matches the morphology of the worker CASENT0922532, from the eastern Amazonian Peru, imaged in AntWeb. This specimen and a soldier were found by Phil Ward on the same plant of the genus *Tachigali* Aubl. (“Tachigalia” on Ward’s label), thus bearing the same collector’s code: P. S. Ward 8657. Based on Oliveira, Powell & Feitosa (2021) key to the Brazilian *Cephalotes*, the *C. dentidorsum* soldier (MEPNINV28455), from Orellana, Ecuador, keyed out as *C. adolphi*. This is not surprising because these two sister species are highly similar (see details below) but inhabit different biomes in terms of mostly plant composition. *Cephalotes adolphi* is restricted to mid-eastern Brazil, mainly the Cerrado biome (De Andrade & Baroni-Urbani, 1999, Oliveira, Powell & Feitosa, 2021), whereas *C. dentidorsum* have only been found in Amazonian habitats (Oliveira, Powell & Feitosa, 2021) did not examine *C. dentidorsum* specimens, it was not the scope of their work, so the characters they propose in their key, which lead to *C. adolphi*, might also lead to *C. dentidorsum* in absence of additional informative characters.

This rarely collected species is phylogenetically placed in the *C. angustus* group *sensu* De Andrade & Baroni-Urbani (1999), confirmed in Oliveira, Powell & Feitosa (2021), but *sensu* Price et al. (2022) is placed in the *angustus* + *fiebrigi* + *bruchi* group. Workers and soldiers of *C. dentidorsum* are highly similar to those of the also rarely collected *C. adolphi*. The workers differ only in the sculpture of the first gastral segment being opaque and with shallow punctae in *C. adolphi*, and smooth and shining in *C. dentidorsum*. However, the soldiers apparently differ in their cephalic index (longer head in *C. adolphi*, slightly wider in *C. dentidorsum*) and in the shape of the anapleural groove (well-impressed in *C. adolphi*, weakly impressed in *C. dentidorsum*). To us, these traits do not seem clear enough to distinguish between these two species. Their status as good taxa, in our opinion, is supported mainly by their differing geographic distribution, and not necessarily by their morphology. Although western Amazonia and the Cerrado differ indeed in their climatic regimes and floristic composition, there is no current evidence of barriers to gene flow between populations in this South American region. Since *C. dentidorsum* and *C. adolphi* have been so rarely collected, and thus far, only two specimens, one of each species, have been CO1 sequenced (see the group *angustus* + *fiebrigi* + *bruchi* in Fig. 2), both with the same BIN (Barcode Index Number), and a pairwise genetic distance of less than 2%, we stress on the need to obtain additional material from both biomes because it remains to be clarified whether they are different species and not populational variants of a single species.

## Discussion

### About the placement of the new species in their respective species-groups

Molecular data usually enables detection of genetic divergence that may not be apparent from morphology alone, and this is particularly relevant for species that exhibit certain phenotypic plasticity or have evolved in isolation (Vrijenhoek, 2009). From our phylogeny, *C. sacha* forms a well-supported group with its similar species *C. alfaroi* and *C. serraticeps* (Fig. 3A). Conversely, we could not reach a consensus as to the placement of *C. esthelae* in a species-group because our morphological observations and genetic analyses lead us to differing conclusions. However, *C. esthelae* exhibits a highly pronounced genetic divergence, >17%, from its known morphologically closest relatives, *C. persimilis*, *C. persimplex*, and *C. grandinosus* (Fig. 4B), which supports the status as a distinct species. Except for a few characters in the worker caste, notably the bigger head of *C. esthelae* as compared to the other three species, they are overall very similar. Thus, we expected these taxa to come out closely related in our *Cephalotes* tree, but they did not. This could be explained, in part, due to the lower phylogenetic resolution of a single mitochondrial locus at deeper nodes, compared to the multi-loci phylogenomic approach, based on Ultraconserved Elements (UCEs), used by Price et al. (2022).

Single mitochondrial loci have inherent limitations for inferring phylogenetic relationships at deep taxonomic levels, these limitations stem from the fact that a single gene captures only one genealogy, which do not necessarily reflect that of the taxon, missing the variance in tree structure across the genome (Main et al., 2024). In addition, mitochondrial genes exhibit a lower substitution rate, making them less phylogenetically informative (Xue et al., 2024). Although our CO1-based tree partially recovered the species composition of *Cephalotes* species-groups in 11 of the 15 clades recognized in Price et al. (2022), its resolution was insufficient to resolve the relationships among species in the genus, and this was particularly evident in the variable position of *C. esthelae* in the two trees depicted in Figs. 2 and 4.

A closer examination of the four sequences of *C. esthelae* in the alignment (File S4) shows various non-homology sites as compared to its known morphologically closest species. The resulting genetic distance may be explained by the long geographic distance, more than a thousand kilometers, separating the Chocoan populations of *C. esthelae* from those of *C. persimilis* from Minas Gerais (Cerrado and Atlantic Forest biomes, southeastern Brazil), and from the Amazonian *C. persimplex*. Both the Andes as dispersal barrier, as well as differing climatic, ecological and physiognomic conditions of the habitats where these species live, are potential explanatory variables. However, with respect to *C. grandinosus*, geographic distance may not be sufficient to explain the observed genetic divergence because the examined records of *C. esthelae* are relatively close to site records of *C. grandinosus*, for example, in Panama and Costa Rica. Also, these two species inhabit the same biome. Other factors like incomplete taxon sampling, structure of the habitats and microhabitats (Abad-Franch et al., 2021), changes in feeding strategies (Guevara-Andino et al., 2025), unidentified geographic-ecological barriers, and even the non-optimal field and museum preserving conditions of the *C. esthelae* specimens, could hold cues to explain such result.

In addition, the sequences of *C. esthelae* showed some ambiguous sites which we assume could be the result of a suboptimal preservation in the museum benches. Before sequencing, these specimens were stored in 75% ethanol for about twenty years, but it was never replaced with new, fresh ethanol (A. Troya, 2025, personal observation). This pattern of DNA fragmentation and degradation has been documented before in museum specimens preserved under such conditions (Ruppert et al., 2023).

Despite these limitations, mitochondrial barcoding remains a valuable tool for species delimitation and discovery of cryptic diversity in ants (Hebert et al., 2004; Schär et al., 2022). The molecular uncertainty associated with *C. esthelae* underscores the need to obtain further fresh material, and thus higher-quality DNA of that species, which may eventually confirm our proposal of its placement in the *pinelii* + *grandinosus* group.

#### Putative signals of divergence in a C. esthelae population

*Cephalotes esthelae and C. sacha are* currently endemics for Ecuador, and further sampling will likely unveil additional site records in both Chocó and in Amazonia. As to *C. esthelae*, we here consider both populations, from Chocó and Amazonia, as belonging to a single species. However, the Amazonian population, represented by a unique individual (Fig. 43, MEPNINV18178), differs from those of Chocó in the shape of the gaster, which is longer in such Amazonian specimen (see comments under *C. esthelae* description), and its gastric pilosity is smaller and less abundant as compared to that of the Chocoan populations. These differences in morphology possibly represent signatures of lineage divergence, which could be occurring due to a potential decline or complete breakdown of interbreeding between the Chocoan and Amazonian populations that are separated by the Andean mountain chain. We do not have DNA data of the Amazonian specimen to support this assumption, however, the Andes as a barrier restricting gene flow (Salgado-Roa et al., 2024; Salazar-Escobar & Villalobos-Moreno, 2024), and the distance separating these populations, approximately 200 km, are factors to consider in future analyses.

Evidence of speciation events promoted by vicariance or dispersion in the context of the Andes uplift in northwestern South America has been demonstrated before with birds (Hazzi et al., 2018), mammals (Vallejos-Garrido et al., 2023), frogs (Mendoza et al., 2015), among other groups, but also with ants, for example, *Atta cephalotes* (Muñoz-Valencia et al., 2022).

Because of the variation in morphology of the Amazonian population of *C. esthelae* as compared to the populations of Chocó, we excluded this specimen from the type material. This responds to the need to avoid taxonomic ambiguities by prioritizing stable diagnostic characters over geographic variants, a criterion applied in groups with morphological plasticity (Bickford et al., 2007).

### Additions to current knowledge in the distribution of other *Cephalotes* species

While examining the geographic distribution of species records in our dataset, we noted that some historical records of *C. alfaroi* originating in Brazil (Roraima and Amazonas), which are currently listed on AntWeb, are misidentifications. To our current knowledge, that species occurs only in Costa Rica and Panama (Basset et al., 2012; AntWeb.org). Besides locality, coordinates and collection date, no other information is provided on AntWeb, and more importantly, the four records are not validated by a taxonomist. Oliveira, Powell & Feitosa (2021) did not mention this species in their *Cephalotes* revision for Brazil. Through the examination of ant collections from Colombia, Ecuador, Peru, and Brazil, we did not find records of *C. alfaroi*. We suggest the Brazilian records could be either *C. sacha* or *C. serraticeps*.

### Implications for the conservation of the habitats home of *C. esthelae* and *C. sacha*

The regions where the new species originate from are highly anthropized and threatened (A. Troya, 2020, personal observation), thus both species may be considered potentially vulnerable. In the Chocó region, mining concessions have caused severe deforestation and fragmentation, while in the Amazonian Lumbaqui town, agriculture expansion and urbanization continue to degrade natural habitats (Morales Corozo, 2022). These impacts threaten the animals that depend on tree canopies for nesting and dispersal (Gindhart et al., 2024). Although the populations of *C. sacha* live in the overall well-preserved Parque Nacional Yasuní, it faces significant threats due to extractive activities, particularly oil exploitation, which compromise both ecosystem integrity and local indigenous communities (Bass et al., 2010). Deforestation remains a leading cause of biodiversity loss in Neotropical insects, disrupting ecological functions and reducing habitat availability (Sánchez-Bayo & Wyckhuys, 2019).

The known populations of *C. esthelae* are represented by only 12 individuals collected from four sites: three in the Chocó region and one in the Amazon. All specimens were collected more than 20 years ago, at a time when these habitats were already under threat from human-driven activities such as commercial deforestation, slash-and-burn agriculture, and illegal mining. Although the sampled Chocoan sites, Salto del Bravo, La Tabla, and Pajonal, were relatively pristine and showed little direct human impact, there was already concern about imminent disturbance, particularly from mining companies seeking to expand operations in the San Miguel River basin, the main access route to these areas. Local Afro-descendant and Indigenous Chachi communities have been increasingly targeted by such companies, often attracted by short-term economic incentives. These temporary benefits, offered in exchange for access to their territories, risk causing long-term damage to fragile ecosystems. Consequently, effective conservation in these areas requires policymakers to adopt frameworks that prioritize the enduring integrity of nature while ensuring sustainable local livelihoods.

## Conclusion

The exploration and documentation of new species is essential to filling gaps in taxonomic-biodiversity knowledge. The present new species hypotheses add to such aim. This study enhances our understanding of *Cephalotes* diversity in Ecuador and highlights the need to conserve endangered habitats and increase field research in understudied regions. To address these challenges, it is crucial to implement conservation strategies that promote habitat restoration, involving local communities in the protection and sustainable management of both degraded and well-preserved ecosystems. Further research will be required to confirm the presence of the current two new species in other Neotropical regions, thus improving our understanding of their distribution and biogeography.

## Supplemental Information

10.7717/peerj.21610/supp-1 Supplemental Information 1 Cephalotes Ecuador checklist. the complete set of museum preserved specimens and literature records we examined.

10.7717/peerj.21610/supp-2 Supplemental Information 2 Sequences metadata. the DNA alignment of the complete set of 295 sequences

10.7717/peerj.21610/supp-3 Supplemental Information 3 Aligned set of 295 CO1 sequences.

10.7717/peerj.21610/supp-4 Supplemental Information 4 Cepahlotes grandinosus + pinelii species-group alignment.

## Additional Information and Declarations

### Data Availability

The following information was supplied regarding data availability:

The data is available in the Supplemental Files.

The sequences of the CO1 gene used in this work are freely accesible through the Barcode of Life Data System—BOLD: https://boldsystems.org/.

- The full list of 294 sequence BIN, ProcessID, and SampleID numbers is available in the Supplemental Files.

### New Species Registration

The following information was supplied regarding the registration of a newly described species:

Publication LSID: urn:lsid:zoobank.org:pub:338E0683-AD9A-441A-80BF-6D38CC5474B1.

Cephalotes esthelae Ilguan & Troya sp. nov.

LSID: urn:lsid:zoobank.org:act:F29AD21C-7825-444C-A69A-DDF51C70D60E.

Cephalotes sacha Yumbay & Troya sp. nov.

LSID: urn:lsid:zoobank.org:act:894642B2-D783-4E44-ABA0-EC3C8809197B.
